# The genomes of pecan and Chinese hickory provide insights into *Carya* evolution and nut nutrition

**DOI:** 10.1093/gigascience/giz036

**Published:** 2019-05-02

**Authors:** Youjun Huang, Lihong Xiao, Zhongren Zhang, Rui Zhang, Zhengjia Wang, Chunying Huang, Ren Huang, Yumeng Luan, Tongqiang Fan, Jianhua Wang, Chen Shen, Shenmei Zhang, Xinwang Wang, Jennifer Randall, Bingsong Zheng, Jiasheng Wu, Qixiang Zhang, Guohua Xia, Chuanmei Xu, Ming Chen, Liangsheng Zhang, Wenkai Jiang, Lizhi Gao, Zhiduan Chen, Charles A Leslie, L J Grauke, Jianqin Huang

**Affiliations:** 1 State Key Laboratory of Subtropical Silviculture, Zhejiang A&F University, No. 666 Wusu St., Lin'an District, Hangzhou 311300, China; 2 Novogene Bioinformatics Institute, No. 38 Xueqing Rd., Haidian District, Beijing 100083, China; 3 Pecan Breeding and Genetics, Agricultural Research Service, United States Department of Agriculture, 10200 FM 50, Somerville, TX 77979, USA; 4 College of Agricultural, Consumer, and Environmental Sciences, New Mexico State University, 3BE Skeen Hall, Las Cruces, NM 88003, USA; 5 School of Life Science, Zhejiang University, No. 866 Yuhangtang Rd., Hangzhou 310058, China; 6 Haixia Institute of Science and Technology, Fujian Agriculture and Forestry University, No. 15 Shangxiadian Rd., Cangshan District, Fuzhou 350002, China; 7 Plant Germplasm and Genomics Center, Germplasm Bank of Wild Species in Southwestern China, Kunming Institute of Botany, Chinese Academy of Sciences, No. 132 Lanhei Rd., Kunming 650201, China; 8 State Key Laboratory of Systematic and Evolutionary Botany, Institute of Botany, Chinese Academy of Science, No. 20 Nanxincun, Xiangshan Rd., Beijing 100093, China; 9 Department of Plant Sciences, University of California, One Shields Avenue, Davis, CA 95616, USA

**Keywords:** Carya, pecan, Chinese hickory, whole-genome sequence, adaptive evolution, nutritional value, genetic improvement

## Abstract

**Background:**

Pecan (*Carya illinoinensis*) and Chinese hickory (*C. cathayensis*) are important commercially cultivated nut trees in the genus *Carya* (Juglandaceae), with high nutritional value and substantial health benefits.

**Results:**

We obtained >187.22 and 178.87 gigabases of sequence, and ∼288× and 248× genome coverage, to a pecan cultivar (“Pawnee”) and a domesticated Chinese hickory landrace (ZAFU-1), respectively. The total assembly size is 651.31 megabases (Mb) for pecan and 706.43 Mb for Chinese hickory. Two genome duplication events before the divergence from walnut were found in these species. Gene family analysis highlighted key genes in biotic and abiotic tolerance, oil, polyphenols, essential amino acids, and B vitamins. Further analyses of reduced-coverage genome sequences of 16 *Carya* and 2 *Juglans* species provide additional phylogenetic perspective on crop wild relatives.

**Conclusions:**

Cooperative characterization of these valuable resources provides a window to their evolutionary development and a valuable foundation for future crop improvement.

## Background

Juglandaceae contains ∼60 known species [1], including many internationally important nut crops such as Persian walnut (*Juglans regia*), pecan (*Carya illinoinensis*), and Chinese hickory (*Carya cathayensis*), as well as valuable hardwood species such as black walnut (*Juglans nigra*). The genus *Carya* consists of ∼20 species worldwide [2, 3] with an intercontinentally disjunctive distribution between East Asia (EA) and eastern North America (ENA) [3, 4]. Pecan and Chinese hickory are the representatives in ENA and EA, respectively, and the only 2 commercially cultivated nut trees of the genus [5, 6]. The nut consumption of pecan and Chinese hickory has been dramatically increasing in recent years, due to their high nutritional value and important health benefits. In comparison with most other nuts, pecan and Chinese hickory contain high quantities of healthful mono-unsaturated fatty acids and a high level of antioxidants with an array of phytochemicals such as phenolic compounds [7, 8]. The nuts are also a rich source of dietary fiber, protein, minerals, and B vitamins—especially thiamine [9]. Recent studies highlight the health benefits of consuming these nuts in conjunction with reduced incidence of multiple diseases such as tumor, edematogeny, hyperglycemia, and hyperlipidemia [10–12]. These beneficial properties have promoted wide cultivation of these species. In the USA, pecan annual yields exceed 130,000 tons with a value >$600 (USD) million annually [13]. In China, Chinese hickory provides annual production of close to 30,000 tons with a farm gate value >$125 million (USD) per year before the year 2010 [14]. Annual production exceeded 40,000 tons in China according to statistics of the Chinese State administration of Forestry and Grassland (unpublished data) in 2017.

In the USA and Mexico, wild pecans were native along the river bottomlands with a wide variance in climate between 30° and 42°N latitude [13]. The natural habitat of pecan ranges from mild to harsh winters and from humid to semi-arid climates with the preference for loamy, well-drained first-class river bottom land [15]. Although wild pecans were well known and considered a delicacy among native and colonial Americans, commercial production of pecans in the USA did not begin until the 1880s [16]. The pecan research activities of the US Department of Agriculture date to the same ;period [17]. The US Department of Agriculture National Collection of Genetic Resources for Pecans and Hickories (NCGR-*Carya*) has collected and maintains >400 pecan cultivars, from 25 US states and Mexico. Some of the cultivars are widely planted worldwide [5]. In 2016, global production of pecan was primarily from Mexico (47%) and the USA (46%), and the rest (7%) from elsewhere including Australia (4%), China (1%), South Africa (1%), and South America (1%) [18].

Chinese hickory is a specialty of the Hangzhou area of Zhejiang province in China, where it has been cultivated for consumption for >500 years since the Ming Dynasty. Both wild and domesticated Chinese hickories grow only in moist valleys at the foothills of the Tianmu Mountains at an elevation of 500–1200 m within the Zhejiang and Anhui provinces in China. In this climatic location they receive full sun in sheltered locations [19]. However, unlike pecan, Chinese hickory has naked terminal buds, making it less adaptable to colder climates [5, 20]. In addition, Chinese hickory has smaller nuts with harder shells and the trees lack tolerance to abiotic stresses such as heat, flooding, drought, and salinity [18], which substantially restrict its commercial cultivation worldwide. Breeding of Chinese hickory is far behind pecan and plateaued at domestication levels until the past decade. However, the species has nucellar embryony (apomixis) that would be valuable for passing down the disease-resistant trait in production [21].

In pecan and Chinese hickory breeding programs, the mission is to preserve, evaluate, and enhance genetic resources and to develop superior cultivars with high disease/insect/(a)biotic resistance and excellent nut quality [22, 23]. To date, several superior cultivars in pecan and Chinese hickory are available with desirable traits such as precocity, high yield, disease and stress resistance, and high nut and kernel quality. [23, 24]. On average, >20 years are required to release a new cultivar by conventional breeding due to extended periods of juvenility [25, 26]. The rapid development of modern biotechnologies, such as genome sequence–based whole-genome–associated analysis and gene editing, may make it possible to shorten the selection process [27].

To develop genetic tools for acceleration of nut tree improvement in *Carya*, whole-genome sequencing of reference genomes was initiated [28], identifying a popular pecan cultivar “Pawnee” [27, 29] for its international value as a base in breeding efforts [30]. Here, we report the completed sequence of 2 *Carya* genotypes: Pawnee and a widely planted representative (ZAFU-1) of Chinese hickory. Tissue samples were collected from a single tree of each. We also re-sequenced 16 *Carya* species (including pecan and Chinese hickory) from EA and ENA, and 2 *Juglans* species (out-group). A hybrid assembly strategy delivered high-quality draft genomes for the *Carya* species. Global analyses of genome features, along with the re-sequencing data in 16 *Carya* species and full assessments of expression changes during embryo development, provide valuable insight into the evolution of the 2 genomes, disjunctive distribution of the genus, their high degree of adaptation to biotic or abiotic stresses, and the accumulation of oils, polyphenols, essential amino acids, and B vitamins. These analyses provide a solid foundation for future studies on improvements of abiotic and biotic stress tolerance, yield, and nutrition in hickories and offer great potential for genome-based breeding of superior cultivars in the genus *Carya*, with the aid of the established explant regeneration techniques [31, 32], and advanced CRISPR-Cas9 gene-editing techniques [33].

## Data Description

To obtain the whole-genome sequences of pecan and Chinese hickory genomes, genomic DNA was extracted from leaf tissues of the pecan cultivar Pawnee (*C. illinoinensis*, NCBI:txid32201) and the domesticated Chinese hickory landrace ZAFU-1 (*C. cathayensis*, NCBI:txid139927) using the cetyltrimethylammonium bromide (CTAB) method. Paired-end (PE) libraries with insert sizes ranging from 250 to 500 bp and mate pair (MP) libraries with insert sizes of 2 and 20 kilobases (kb) were constructed according to the manufacturer's instructions (Illumina, San Diego, CA). All constructed libraries were sequenced on Illumina HiSeq X-ten. Single-molecule real-time (SMRT) sequencing of long reads on a Pacific Biosciences (PacBio) RS II platform (PacBio, Menlo Park, CA, USA) was used to assist the subsequent *de novo* genome assembly process. First, a 20-kb insert size SMRTbell library was prepared following the manufacturer's protocol (PacBio). Then, these libraries were sequenced on PacBio RS II platform using the P6 polymerase/C4 chemistry combination, based on the manufacturer's procedure. Sequencing statistics for all libraries are outlined in Additional file 1: Table S1. In total, ∼157 and 161 gigabase (Gb) reads were generated on Illumina platforms, and 21.72 and 25.80 Gb reads were generated on PacBio platforms of ZAFU-1 and Pawnee. Quality control involved the following steps: (i) removing reads with ≥10% unidentified nucleotides; (ii) removing reads with >20% bases having Phred quality <5; (iii) removing reads with >10 nucleotides aligned to the adapter, allowing ≤10% mismatches; and (iv) removing putative polymerase chain reaction (PCR) duplicates generated by PCR amplification during the library construction process (i.e., reads 1 and 2 of 2 PE reads that were completely identical). Finally, ∼135.29 and 123.31 Gb of Illumina clean data and 25.75 and 21.68 Gb PacBio clean data were obtained for the *de novo* assemblies of the Pawnee and ZAFU-1 genome, respectively.

The details about sample collection, library construction, sequencing, assembly, gene prediction, and annotation can be found in the Materials and Methods.

## Results

### Genome sequencing, assembly, and quality assessment

To obtain high-quality reference genome sequences, we sequenced the genomes of Pawnee and ZAFU-1 (Additional file 1: Table S2) using the HiSeq X-Ten sequencing platform from Illumina and single-molecule real-time (SMRT) sequencing technology from PacBio. In total, >187 and 178 Gb of sequence raw data, equivalent to ∼288× and ∼248× genome coverage of Pawnee and ZAFU-1, respectively, were used to assemble the genomes (Additional file 1: Table S3). The assemblies contain 3,860 (Pawnee) and 5,449 (ZAFU-1) scaffolds (≥2 kb), with scaffold N50 of 1.08 Mb (Pawnee) or 1.22 Mb (ZAFU-1), with 90% of the assembled genomes contained in 682 (Pawnee) or 732 (ZAFU-1) scaffolds (Table 1; Additional file 1: Table S4). The total assembly sizes of 651.31 Mb for pecan and 706.43 Mb for Chinese hickory are close to the size estimated by means of K-mer statistics (Table 1; Additional file 2: Fig. S1; Additional file 1: Table S5) and flow cytometry (Additional file 2: Fig. S2). The assembled sequences cover >97% of the genome size. The assembled sizes are slightly larger than the estimated size for Pawnee probably due to the relatively high heterozygosity.

**Table 1:** Assembly summary of pecan (Pawnee) and Chinese hickory (ZAFU-1) genomes

Parameter	ZAFU-1	Pawnee
Estimated genome size (Mb)*	721.33	649.75
Total assembly (Mb)	706.43	651.31
Longest scaffold (Mb)	4.95	4.92
No. of contigs**	15,789	17,542
N50 contig length (Kb)	101.58	77.23
N50 contig count	1,879	2,388
No. of scaffolds**	5,449	3,860
N50 scaffold length (Mb)	1.22	1.08
N50 scaffold count	174	188
N90 scaffold length (Kb)	137.39	210.68
N90 scaffold count	732	682
Missing bases (%)	1.61	0
Protein-coding genes	32,907	31,075
Repeat sequence (Mb/%***)	381.01/53.67	334.55/50.43
microRNAs	373	378
tRNAs	558	571
rRNAs	362	198

^*^Show the revised genome size estimation.

^**^Show the number of contig or scaffold ≥2 kb.

^***^Show percentage of assembled genomes.

Examination of the guanine-cytosine content distribution indicated that our data were sequenced randomly (Additional file 2: Fig. S3). Read coverage statistics showed that >96.8% of Illumina short-insert reads can be aligned back to the final assemblies for both species (Additional file 1: Table S6). Assessment of gene coverage by CEGMA [34] and BUSCO V [35] revealed that >94% of single-copy genes were assembled completely (Additional file 1: Tables S7 and S8), which is suggestive of complete assemblies and annotation. These metrics indicate that our assemblies are of high quality and have low error rates.

### Genome annotation

Comprehensive repeat sequences of the Pawnee and ZAFU-1 genomes revealed >50% repetitive sequences (50.43% for pecan and 53.67% for Chinese hickory), of which ∼85% are transposable elements (TEs) (Table 1; Additional file 1: Table S9). Long terminal repeats (LTRs) make up the majority of the TEs in both genomes, of which *Gypsy*-like and *Copia*-like elements make up 15.06% and 15.41% in Pawnee and 15.91% and 18.54% in ZAFU-1, respectively (Additional file 1: Table S10). In comparison, the total TE proportions in pecan and Chinese hickory were much higher than in the walnut genome (8.4% *Gypsy*-like and 6.57% *Copia*-like) [36]. However, the ratio of *Gypsy*-like LTRs to *Copia*-like LTRs is 0.98 to 1 in pecan and 0.86 to 1 in Chinese hickory, much lower than in walnut and grass species [36, 37].

Predicted protein-coding genes in the Pawnee and ZAFU-1 genomes (Table 1) were annotated using a combination of *ab initio* prediction, homology search, and *de novo* assembled transcripts gathered from RNA sequencing of multiple tissues. The hybrid gene prediction protocol delivered 31,075 gene models in the pecan genome and 32,907 in the Chinese hickory genome (Table 1; Additional file 1: Table S11-S12). Statistics on gene structure features showed that the arithmetic average transcript lengths of annotated genes (not including untranslated regions) were 4,223 bp (Pawnee) and 4,313 bp (ZAFU-1), much longer than in other reference genomes, except apple and grape (Additional file 1: Table S13). The arithmetic average number of exons per gene and the average CDS length were close to those of the selected species. Predicted genes were functionally annotated by a consensus approach, revealing up to 95.7% (Pawnee) and 94.7% (ZAFU-1) of the genes having homologs with known functions in 4 different public databases (Additional file 1: Table S14).

We also identified similar copies of microRNAs or transfer RNAs (tRNAs) between Pawnee and ZAFU-1 genomes (Table 1, Additional file 1: Table S15). Interestingly, the ZAFU-1 genome encodes a larger number of small nuclear RNAs (snRNAs) than does the Pawnee genome. The snRNAs primarily function for chemical modifications of other RNAs, and snRNAs U3 and U6 in the CD-box subclass were associated with methylation [38]. In the ZAFU-1 genome, ∼81% of the snRNAs belong to the CD-box subgroup and may contribute to the function of environmental stress tolerance.

### Evolution of *Carya* genus and the 2 nut tree genomes

#### Phylogenetic reconstruction

**Figure 1:**
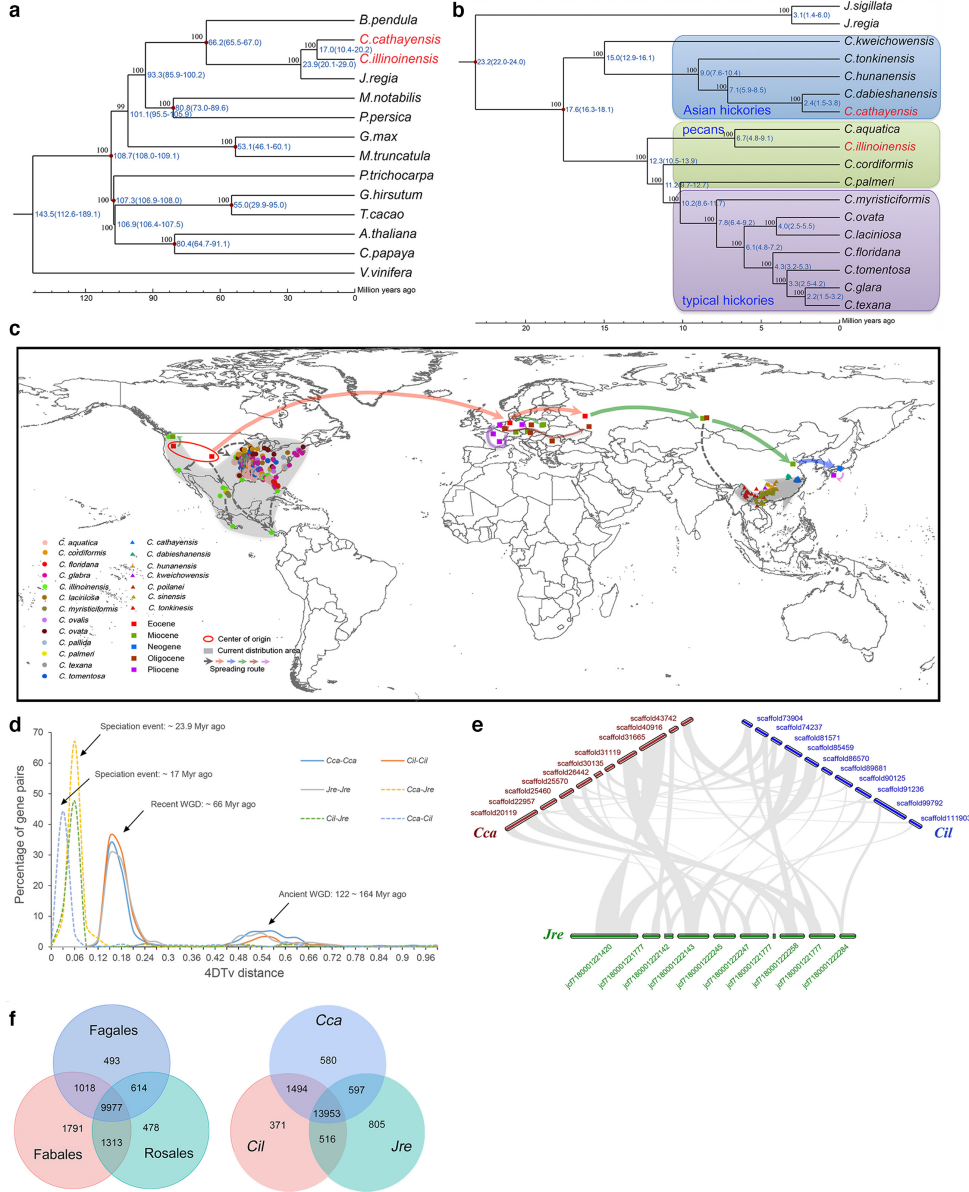
Evolutionary analyses of the *Carya* genus and the genomes of pecan and Chinese hickory. (a) Phylogeny of pecan and Chinese hickory and 12 other genome-sequenced species in Rosids. (b) Phylogeny of 16 *Carya* species (ML tree) with 2 *Juglans* species as out-group. (c) Geographical distribution of both fossil and extant *Carya* species. (d) WGD and speciation in genomes of pecan, Chinese hickories, and walnut based on 4DTv. (e) Syntenic analysis of pecan (Cil), Chinese hickory (Cca), and walnut (Jre). Only the scaffolds with syntenic relationship were shown (including 10 longest scaffold with syntenic blocks). (f) A Venn diagram illustrating shared and specific gene families in pecan, Chinese hickory, *J. regia*, and other representative species in Fagales, Fabales, and Rosales.

Phylogenetic reconstruction of 14 genome-sequenced species from Fabales, Fagales, and Rosales in Rosids, as well as grape, revealed a common ancestor of pecan, Chinese hickory, and *J. regia* ∼20–29 million years ago (MYA) (Fig. 1a). The split between pecan and Chinese hickory is estimated to have occurred 10.4–20.2 MYA. Re-sequencing data from 16 *Carya* species and 2 *Juglans* species were mapped to Chinese hickory genome sequences. Of the 16 *Carya* species (Additional file 1: Table S2), the mapping rate ranged from 72.32% to 96.95%, but the rates for the 2 *Juglans* species were only 34.90% and 40.58% (Additional file 1: Table S16), indicating a recent divergence time for *Carya* species. Furthermore, an interspecific phylogenetic topology of 16 *Carya* species was built with *J. regia* and *J. sigillata* as out-group (Fig. 1b). The phylogenetic tree revealed 2 major clades, in congruence with the intercontinental disjunctive distribution of our previous reports [3]. Integrating these results with our previous reports [3], we generated the most comprehensive geographical distribution map, to date, with all 20 putative hickory species (not all presented in this research) and the fossil record sites (Fig. 1c). The phylogenetic relationship of the examined species between and within morphological sections is well correlated with the geographic distribution, with clear distinction between EA and ENA *Carya*. Further inclusion of *Carya sinensis* (section Rhamphocarya) would be valuable and might clarify its taxonomic position. If *Carya poilanei* still exists, it would be very valuable to find, conserve, and evaluate it to determine whether it should be included in Sinocarya or Apocarya [39]. Clear genomic distinction between the ENA sections is arguable.

#### Genome evolution

On the basis of the accumulated transversion rate at 4-fold degenerate synonymous sites of the third codon position values (4DTv) of the duplicate gene pairs, 2 whole-genome duplication (WGD) events (at ∼0.15 and ∼0.51) were identified in the orthologous segments within the genomes of pecan, Chinese hickory, and walnut (Fig. 1d). This suggests that the 3 species shared a common ancestor that experienced both a recent duplication event (∼66 MYA) and an ancient gamma-triplication event (122–164 MYA) in an angiosperm ancestor. The speciation events occurred ∼23.9 MYA (between walnut and pecan or Chinese hickory) and ∼17.0 MYA (between the 2 *Carya* species), being consistent with the estimated divergence time of phylogenetic reconstruction. Syntenic analysis revealed that 343 and 342 syntenic gene blocks (≥5 genes per block) were found, which were involved in 10,530 and 7,682 paralogous gene pairs in the pecan and Chinese hickory genomes, respectively (Fig. 1e; Additional file 1: Table S17). A high proportion of paralogous gene pairs reside in these collinear blocks, providing strong support for the co-occurrence of WGD events.

Although LTR TEs contribute to most of the repetitive sequences in the pecan and Chinese hickory genomes (Additional file 1: Table S10), the relationship of LTRs and genome expansion is still unknown. The insertion time of all LTRs was dated by means of divergence analysis for further understanding of genome expansion events in both species. As a result, the LTR burst time (∼8 MYA) matches the speciation time of Chinese hickory (Fig. 1d; Additional file 2: Fig. S4). However, the number of LTRs reached its maximum 2–3 MYA and subsequently decreased in pecan. This reflects that the proliferation of LTRs contributed more to the expansion of the Chinese hickory genome than to pecan after the divergent event between them but did not directly contribute to the 2 major WGD events.

Comparative analyses showed that the 4 Fagales species—pecan, Chinese hickory, walnut [36], and silver birch [40], share 9,977 gene families with other genome-sequenced Rosids species and 13,953 were common to Fagales species (Fig. 1f). In total, 371 gene families were specific to pecan and 580 to Chinese hickory. Gene ontology (GO) terms and Kyoto Encyclopedia of Genes and Genomes (KEGG) pathway enrichment of the genes in the unique gene families highlighted the functions pertaining to organic biosynthetic processes and signal transduction (GO), and linoleic acid metabolism (KEGG) in the Chinese hickory genome (Additional file 2: Fig. S5; Additional file 1: Table S18). In the pecan genome, the significantly enriched GO term was the reactive oxygen species metabolic process, with no other significantly enriched pathway (Additional file 2: Fig. S5; Additional file 1: Table S18). Gene family expansion-contraction analyses among the 13 Rosids species and grape revealed only 16 expanded gene families in pecan and 59 in Chinese hickory (Additional file 2: Fig. S6). GO terms and KEGG pathway enrichment analyses of genes in the expanded gene families in pecan and Chinese hickory were also performed (Additional file 2: Fig. S7; Additional file 1: Table S19). Significantly enriched genes were involved in wide GO terms in both species; some of the genes function on pathways related to reactive oxygen species cleavage (peroxisome and glutathione metabolism), plant-pathogen interactions in pecan, and biosynthesis of secondary metabolites and flavonoid biosynthesis in Chinese hickory.

#### The genomes and stress adaptation

**Figure 2:**
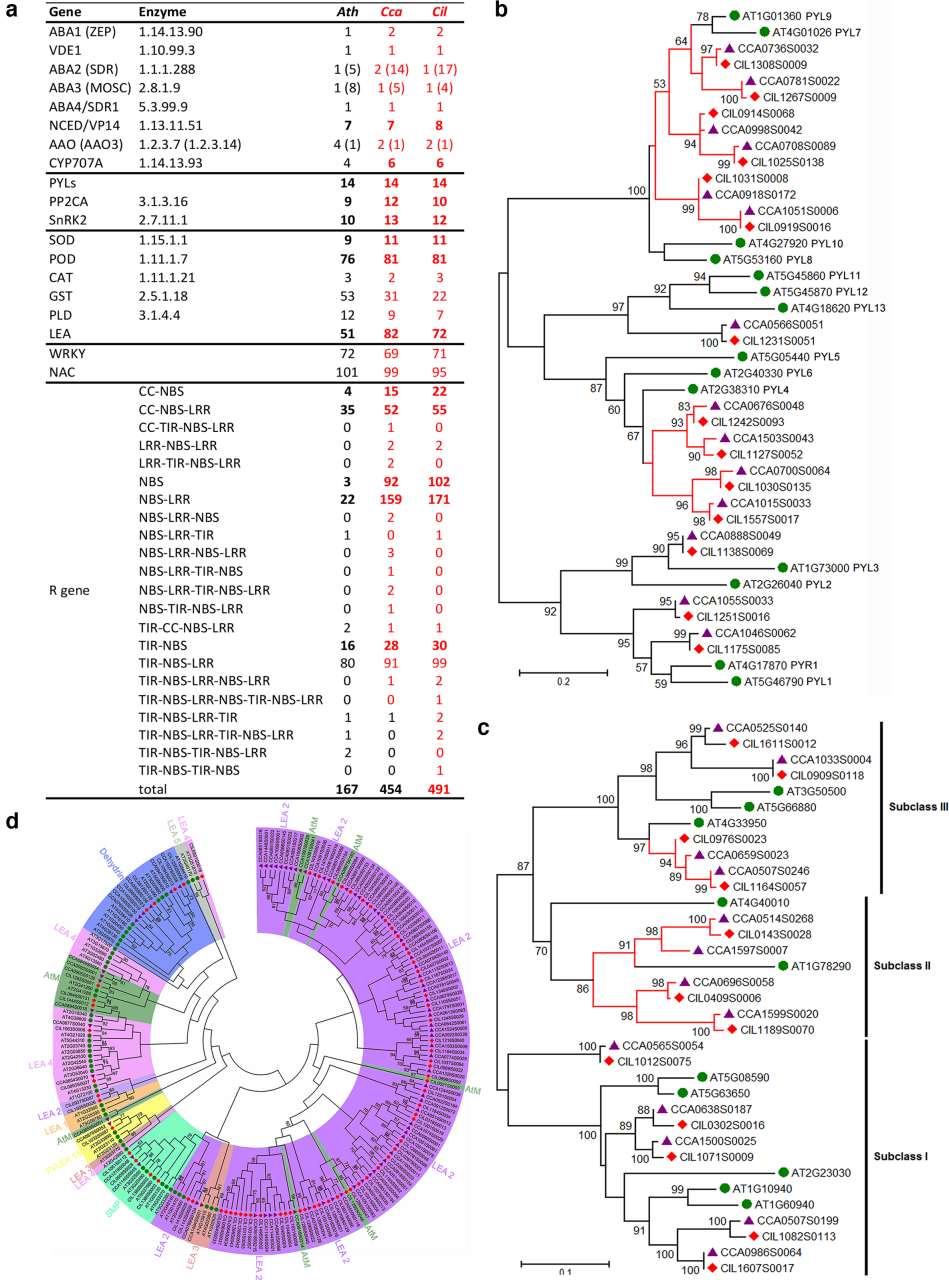
Selected stress-associated genes in pecan and Chinese hickory. (a) Statistics of important drought-associated genes in 6 sequenced grass genomes. (b) ML tree of PYLs in pecan, Chinese hickory, and *Arabidopsis*. (c) ML tree of SnRK2 genes in pecan, Chinese hickory, and *Arabidopsis*. (d) ML tree of LEA protein genes in pecan, Chinese hickory, and *Arabidopsis*.

To explain the molecular basis of abiotic stress adaptation in both species, we identified the genes related to abscisic acid (ABA) metabolism and signaling pathways in both genomes. Only the genes encoding ABA1 and late embryo abundant (LEA) proteins, and R genes had significantly expanded copies (Fig. 2a). Detailed phylogenetic analysis of the core components of ABA signaling (Additional file 2: Fig. S8) demonstrated remarkable duplication on clades of PYL7–PYL9-like and PYL4-like ABA receptor genes (Fig. 2b) and subclass II and subclass III SnRK2 genes (Fig. 2c). These might account for the enhanced abiotic or biotic stress resistance observed in both species. As marker genes in response to biotic stress, a large number of R genes were identified in both genomes (Fig. 2a) and there were more R genes in Chinese hickory than in pecan. More R genes might be a reflection of the higher disease resistance and the adaptation to subtropical climate of Chinese hickory. A maximum likelihood (ML) tree of LEA protein-encoding genes revealed an extreme expansion in Group 2 LEAs (Fig. 2d), probably the genetic basis of enhanced cellular structure protection under stresses and the high content of storage protein in the nuts of both species.

### Identifying differentially expressed genes

The kernels of pecan and Chinese hickory are nutritious and economically valuable. To reveal the mechanisms of the nutritional accumulation, we conducted transcriptomic analysis during embryo development and identified differentially expressed genes (DEGs) in pecan and Chinese hickory. The expression levels of DEGs vary significantly among 3 representative stages of embryonic development: i.e., the early stage (PEY1 and HEY1), the stage with fully extended cotyledons (PEY2 and HEY2), and the fully matured stage of the embryos (PEY3 and HEY3) in pecan and Chinese hickory. In total, we identified 1,043 DEGs (395 up- and 648 downregulated) in PEY2 vs PEY1, and 2,524 DEGs (1,215 up- and 1,309 downregulated) in PEY3 vs PEY2 in pecan. In Chinese hickory, 1,570 DEGs (407 up- and 1,163 downregulated) in HEY2 vs HEY1, and 554 DEGs (261 up- and 293 downregulated) of HEY3 vs HEY2 were identified (Additional file 3). We performed GO enrichment using the significantly up- and downregulated DEGs based on the hierarchical clustering of DEGs. In pecan, the upregulated DEGs from the developmental stage PEY1 to PEY2 centered in the carboxylic acid biosynthetic process, fatty acid metabolic process, lipid particle, acetyl coenzyme A carboxylase (ACCase) activity, and ligase activity; the downregulated DEGs, meanwhile, were related to nucleosome assembly, chromatin assembly or disassembly, and non–membrane-bounded organelles (Additional file 2: Fig. S9). From PEY3 to PEY2, the upregulated DEGs centered in peroxisomes, embryo development, etc.; and the downregulated DEGs were related to the fatty acid metabolic process, glycolysis, cytoskeletal part, etc. (Additional file 2: Fig. S10). Comparing HEY2 vs HEY1, the upregulated DEGs centered in the superoxide metabolic process, lipid particles, nutrient reservoir activity, etc.; and the downregulated DEGs were related to DNA packaging, microtubule-based process, non–membrane-bounded organelles, etc. (Additional file 2: Fig. S11). Comparing HEY3 vs HEY2, the upregulated DEGs centered in the oxidation-reduction process; and the downregulated DEGs were related to glycolysis, microtubules, potassium ion binding, etc. (Additional file 2: Fig. S12).

### Pecan and Chinese hickory oil-abundant tree nuts

**Figure 3:**
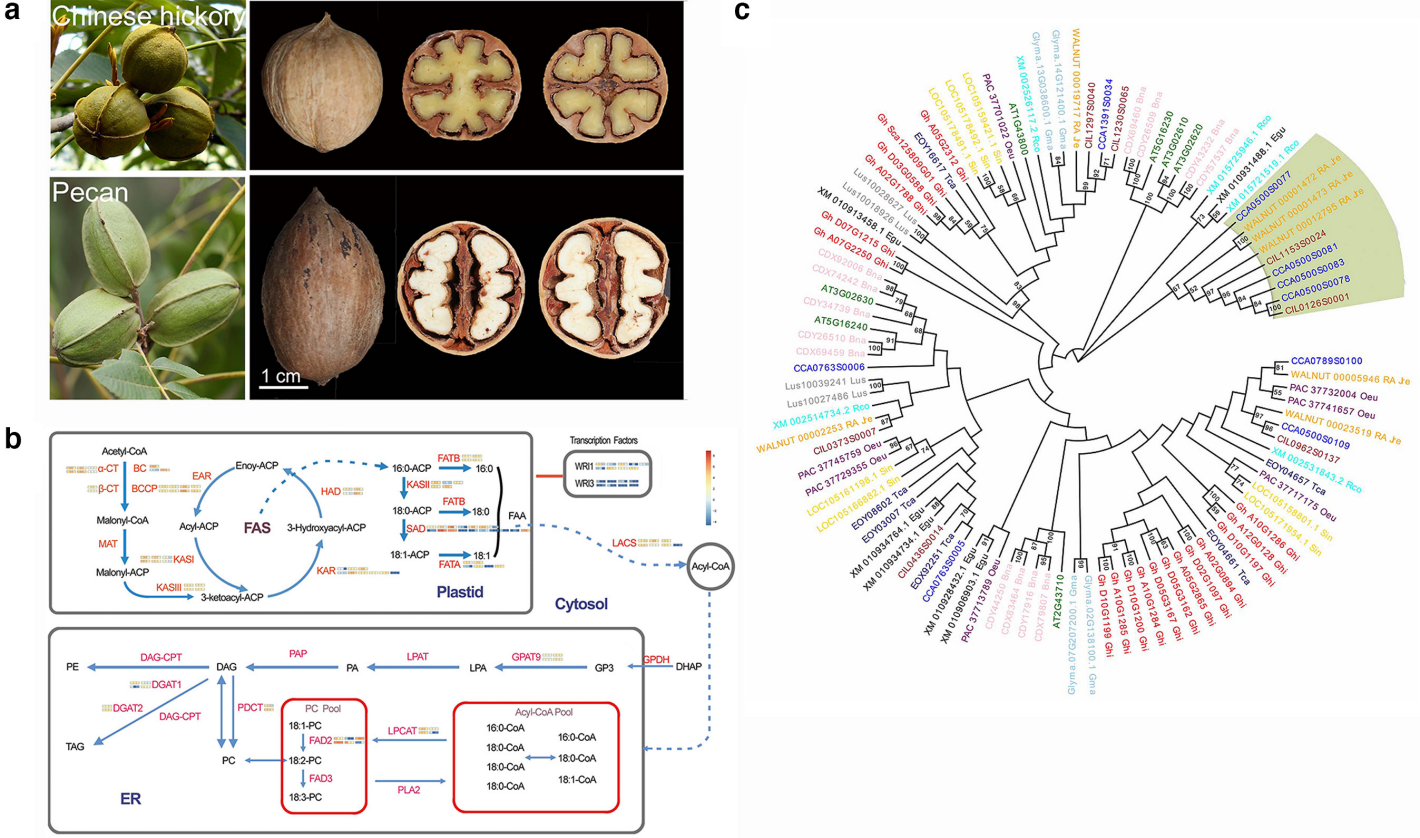
Fruits and seeds, oil metabolism overview, and the detailed analyses of expanded key gene families. (a) The fruits and seeds of pecan and Chinese hickory. (b) Oil biosynthesis pathway, combined with the gene copy number and transcription abundance shown by boxes in pecan (upper boxes) and Chinese hickory (lower boxes). (c) Comparative analyses on gene structure and evolution of SADs in pecan and Chinese hickory against those in *Arabidopsis*.

One of the key healthful traits of pecan and Chinese hickory nuts is an abundance in oil (>70% of fresh weight) (Fig. 3) [41]. To reveal the underlying genetic mechanism, we identified all genes involved in fatty acid metabolism in the pecan and Chinese hickory genomes by using *Arabidopsis* homolog protein sequences as query (Additional file 1: Table S20). Compared with other diploid oil plants [42, 43], the Chinese hickory genome harbored more genes involved in oil accumulation, for both fatty acid *de novo* synthesis and triacylglyceride (TAG) assembly pathways, but less than that of soybean due to an additional WGD event ∼13 MYA [44]. The pecan genome ranked third in the total number of genes related to oil synthesis (Additional file 1: Table S20). Most of the oil synthesis–related gene homologs in pecan and Chinese hickory are abundant in transcripts during embryo development, suggesting an important role in the synthesis of unsaturated fatty acids.

Further analyses revealed remarkable expansion of gene families encoding key enzymes and important transcription factors in pecan and Chinese hickory and other selected oil plants (Fig.   3b; Additional file 1: Table S20). One of the expanded gene families encodes ACCases, which convert acetyl coenzyme A to malonyl coenzyme A as a rate-limiting enzyme in fatty acid *de novo* synthesis [45]. The plastidic heteromeric ACCase includes 4 subunits, namely, α-CT, β-CT, BC, and BCCP. Pecan and Chinese hickory genomes harbor more copies (9 and 10, respectively) than most other oil plants but are similar to soybean (Additional file 1: Table S20). Transcriptomic analysis showed significant transcript accumulation of the homologs of *ACCase* and *DGAT* at oil accumulation stages during embryo development in both species. The expanded gene copies and their high expression levels likely impact the high oil level in the nuts of pecan and Chinese hickory (Fig. 3b; Supplementary Tables S21 and S22) [46]. ∆-9-stearoyl-ACP desaturase (SAD) is a crucial enzyme for *de novo* synthesis of unsaturated fatty acids in oil plants [47], and the transcript abundance of encoding genes was enriched only in the Chinese hickory genome (Fig. 3b; Additional file 1: Table S22). Phylogenetic analysis of the family revealed a unique clade to species in Juglandaceae (Fig. 3c). Furthermore, these homologs were abundant in transcripts during embryo development, indicating an important role in the synthesis of unsaturated fatty acids (Fig. 3b; Additional file 1: Tables S21 and S22).

Pecan and Chinese hickory have more copies of 2 kinds of key transcription factors, WRIs and PIIs, than other diploid oil plants, except for soybean (Additional file 1: Table S20). Of them, *WRI1* and *PII* are significant accumulated transcripts during embryo development in both species (Fig. 3b). Expression of fatty acid desaturase (FAD) family members regulates different fatty acid components and ratios [48]. FAD3 catalyzes the critical step of converting linoleic acid (18:2) to linolenic acid (18:3) while FAD5 plays a major role in the transformation of palmitic acid (C16:0) to palmitoleic acid (C16:1) [49]. In contrast to other oil plants, no FAD3 or FAD5 homolog is encoded by the oil palm genome, whose seeds are rich in saturated fatty acids. This suggests that the high levels of unsaturated fatty acid in Chinese hickory, pecan, and other oil plants are probably due to the additional FAD members. The expansion and high expression levels of genes related to unsaturated fatty acid biosynthesis provide genomic evidence and genetic basis for the high proportion of unsaturated fatty acids in *Carya* nuts.

### Pecan and Chinese hickory as polyphenol-, arginine- and B vitamin–rich tree nuts

**Figure 4:**
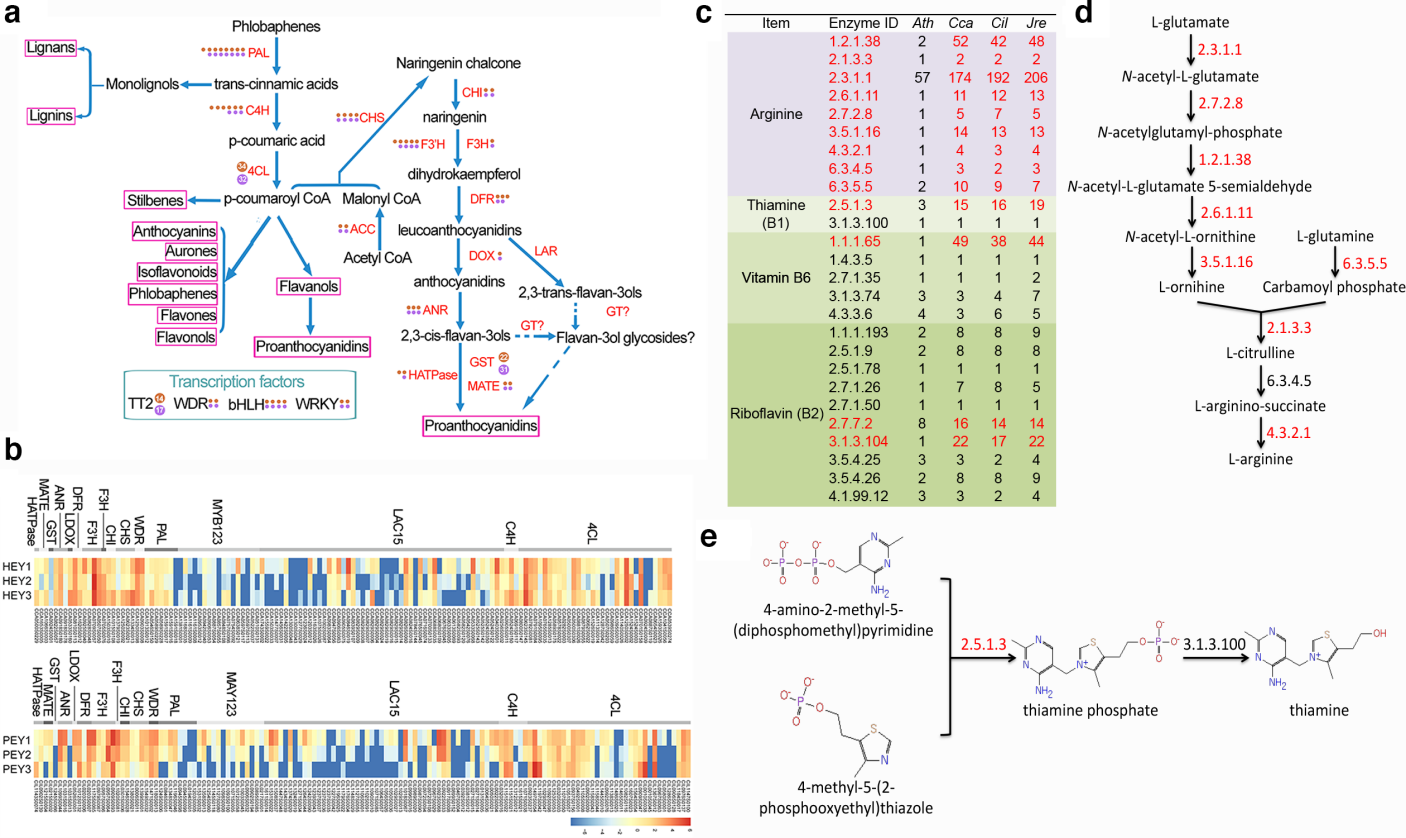
Key genes involved in polyphenol, arginine and vitamin B metabolism in pecan and Chinese hickory. (a) Polyphenol biosynthesis pathway shows the gene copy number encoding key enzymes and transcription factors by solid dots or number in pecan (red) and Chinese hickory (purple). (b) Heat map of gene expression profiles of key genes in polyphenol biosynthesis pathway during embryo development in pecan and Chinese hickory. Gray blocks indicate missing data. (c) Gene copy number of enzymes involved in the biosynthesis of arginine and vitamin B1 in *Arabidopsis* and 3 Juglandaceae species. (d) Diagrams showing the key steps in the biosynthesis of arginine. (e) Diagrams showing the key steps in the biosynthesis of thiamine.

Polyphenols, as secondary metabolites and potential antioxidative compounds, are involved in multiple aspects of plant development and defense [50] and have additional value for human health [47, 51]. Phenolic compounds are primarily derived from flavonoid biosynthesis, which includes the anthocyanin, proanthocyanidin, and flavonol pathways [52] (Fig. 4a). We identified the genes that are related to anthocyanin biosynthesis and regulation among 11 selected species including *Arabidopsis*, pecan, and Chinese hickory (Additional file 1: Table S23). We found that most of the gene families had no apparent significant expansion among the selected species beyond that seen in *Arabidopsis*. Chalcone synthase (CHS), the first enzyme triggering the pathway, has 1 or 2 additional copies in pecan, Chinese hickory, and walnut (Additional file 1: Table S23). Leucoanthocyanidin reductase (LAR), a key enzyme in proanthocyanidin biosynthesis, also showed substantial expansion and was not encoded by the *Arabidopsis* and tomato genomes (Additional file 1: Table S23). Both the CHS and LAR gene families, together with WRKY transcription factors, exhibited a Juglandaceae-specific expansion (Additional file 2: Figs S13 and S14; Additional file 1: Table S23). Expression profile analysis showed that the majority of genes involved in the proanthocyanidin biosynthesis pathway had a relatively high expression level during embryo development in both pecan and Chinese hickory (Fig. 4b). These results provide genomic support for pathways leading to the high polyphenol content in the nuts.

Pecan and Chinese hickory nuts are valued not only for their high oil content but also for their high protein content and richness in essential amino acids [10]. We examined the key genes involved in biosynthesis of 10 amino acids, including 8 essential, 1 semi-essential (arginine), and 1 essential only for children (histidine). Of them, arginine is the most abundant and there are 9 enzymes involved in its biosynthesis. All of the encoding genes of the arginine enzymes have remarkably expanded copy number compared with *Arabidopsis* and have medium copies among diploid oil plants (Fig. 4 c; Additional file 1: Table S24). Most genes encoding enzymes involved in other 9 amino acid biosynthesis have similar trends in copy numbers in both pecan and Chinese hickory genomes (Additional file 1: Table S24).

Pecan and Chinese hickory nuts also contain high levels of vitamin B, especially thiamine (vitamin B1) [10]. Thus, we examined the key enzymes involved in vitamin B biosynthesis of the *Carya*, and walnut genomes, and all other sequenced oil associated plant genomes and *Arabidopsis* as well as rice (Fig. 4c and 4e; Additional file 1: Table S25). We found that gene copies encoding 1 of the enzymes involved in vitamin B1 biosynthesis (EC 2.5.1.3), 1 enzyme catalyzing vitamin B6 biosynthesis (EC 1.1.1.65), and 2 enzymes generating vitamin B2 (EC 2.7.7.2 and EC 3.1.3.104) are remarkably higher than those in *Arabidopsis* but in the middle of the range among diploid oil plants (Fig. 4c and 4e; Additional file 1: Table S25).

## Discussion

### Evolutionary history of *Carya* species

The genus *Carya* exhibits a remarkable disjunctive distribution between EA and ENA, which offers a model for understanding the phylogenetic relationship between EA and ENA species. By combining the analysis of 8 plastid and 2 nuclear loci in 16 *Carya* species with fossil and morphological data, we investigated the phylogenetic relationships between EA and ENA species and reconstructed the historical biogeography of *Carya* [3]. The results clarified the boreotropical flora hypothesis and North Atlantic land bridge as a crucial route for the spread of *Carya* species from North America to Europe to EA. Although the results from Zhang et al. [3] strongly supported the intercontinental disjunctions in *Carya*, use of only 10 loci is still not sufficient for fully exploring the phylogenetic pattern of intracontinental species in EA and ENA.

By comparison, our high-quality genome sequences of pecan and Chinese hickory, together with the re-sequencing data from 14 other *Carya* species, offer a much greater number of molecular loci throughout the genome. These attributes are important for enhancing phylogenetic accuracy and the reliability of phylogenetic relationships among *Carya* species. The phylogenetic tree (Fig. 1b) offers strong support for the intercontinental disjunctions in *Carya* and the inferences regarding origin and distribution during *Carya* evolution, as suggested by Zhang et al. [3]. The phylogenetic relationship among species within morphological sections is well correlated with the geographic distribution, at least for the Asian hickories evaluated. Distinction between ENA sections Apocarya and Carya is clear on the basis of morphology but is not evident by a clear phylogenetic division of the species evaluated here.

Integrating these analyses with the previous and recent studies as well as the fossil record [3], we generated the most comprehensive geographical distribution map to date, containing 20 extant hickory species and the fossil record sites (Fig. 1c). In accordance with our previous discussion [3], the extant *Carya* species formed 2 distribution centers in EA and ENA. All of the results allow us to speculate that the present disjunctive distribution of *Carya* species between EA and ENA might be the result of extinctions in large parts of its former ranges. *Carya* was more broadly distributed across North America and dispersed to western Europe via the North Atlantic land bridge in the Miocene, and continually spread to central Europe and Asia in the Miocene, and to Japan in the Neogene (Fig. 1c). Subsequently, climatic cooling resulted in the original extinction events that caused the range fragmentation in *Carya* and ultimately led to speciation. As the representatives of EA and ENA *Carya* species, pecan and Chinese hickory depict independent evolution of discontinuously distributed species originating in EA and North America. After diverging from their common ancestor, they have been evolving independently, representing clades that share distinctive biological and physiological characteristics and ecological adaptation.

### Adaptive evolution of pecan and Chinese hickory

The Asian *Carya* species, including Chinese hickory, have restricted geographical distributions with specific ecological requirements [51]. Chinese hickory is restricted to a narrow area of subtropical climate in eastern China [53] but has resistance to scab [54]. In contrast, *Carya* species in North America, including the native pecan, are adapted to a wide range of climate types from mild to harsh conditions and exhibit high resistance to multiple abiotic stresses [55]. This wide adaptability has resulted in worldwide commercial cultivation and numerous cultivars and hybrid lines of pecan [26, 56]. Pecan is adapted across climatic regions with a wide variety of precipitation, temperature, and soils. It is a riparian species that thrives on soil moisture but occurs on alkaline calcareous soils of west Texas as well as more neutral soils of the Mississippi River and its tributaries in the east. Chinese hickory requires the moist conditions of rainy subtropical or tropical areas. Morphologically, the buds of North American *Carya* species are covered by bud scales [21], which provide protection for young apical meristems and contribute to the adaptation to wider latitudes. In contrast, the naked buds of Asian *Carya* species have restricted their distribution to subtropical and tropical areas. Comparative analysis shows that the expanded gene families in pecan are significantly enriched in functions associated with response to oxidative stress, biotic defense response, stimulus, wounding, metal ion exposure, etc. However, the expanded gene families in Chinese hickory are mainly associated with plant-pathogen interaction. This analysis suggests the genetic basis for adaptation to climates and stress resistance between pecan and Chinese hickory. The presence or absence of bud scales are results, not causes.

The phytohormone ABA, protective LEA proteins, antioxidative enzymes (such as superoxide dismutases, peroxidases, and phospholipase Ds), detoxifiers (such as glutathione S-transferases), and R genes are often considered to be key components of response to abiotic and/or biotic stress [50, 57]. Similar copies of the key genes for enzymes in ABA metabolism and signaling were identified in the pecan and Chinese hickory genomes. However, the detailed analysis of core components of ABA signaling, PYL receptors and SnRK2 kinases, revealed a large expansion of subclasses of the PYL7-PLY9 clade, the PYL4 clade, and SnRK2 subclass II–III, compared with *Arabidopsis*. The extreme expansion of the R genes and LEA proteins, specifically expanded group 2 LEAs in both genomes, enhanced the protective roles under stress conditions. These probably reflect increased resistance to abiotic and biotic stresses in woody plants. Meanwhile, the increase in copies of R genes in pecan relative to Chinese hickory provides the genetic basis for the ability to cope with biotic stress in this species.

Moreover, biogeographic studies suggest that the extent of fatty acid unsaturation in oil seeds played an important role in temperature adaptation on both a micro- and macro-evolutionary scale [58, 59]. It is worth mentioning that stearoyl-acyl carrier protein D9/desaturase 6 (SAD9/DES6), a fatty acid desaturase, plays vital roles in drought and hypoxia stress in *Arabidopsis* [60]. Here, the existence of 2 DES6 genes in the pecan genome versus a single copy in Chinese hickory is consistent with the stronger resistance to drought and hypoxia found in pecan.

### Genetic basis of nut nutritional value

As delicious and nutritional foods, pecan and Chinese hickory nuts are valued for not only their high unsaturated fatty acid content and anti-oxidative polyphenols but also richness in proteins, fiber, minerals, and vitamins. LEA proteins, as the major seed storage proteins, not only play protective roles in the responses to stresses but also provide resources of nutritional value. The substantial expansion of LEA encoding genes in both genomes may provide an explanation of the observed high protein content in the nuts of pecan and Chinese hickory. Although our transcriptomic analyses of biosynthesis pathways of oil and proanthocyanidins during the development of embryos of pecan and Chinese hickory have provided valuable clues [46, 61], the molecular mechanisms are still unknown. The specific expansion on genes encoding several key enzymes in oil biosynthesis, combining with the expression profiles, provided the fundamental basis to further investigate the underlying mechanism.

The nutritional value and health benefits of pecan and Chinese hickory offer potential for enhanced food security. The high-quality reference genome assemblies presented here will accelerate improvements in these 2 nuts. Major breeding objectives for their improvement include the development of shorter plants with more branches and more and larger fruits, increased water and biotic stress resistance, and the introgression of the sweet phenotype into commercial varieties. The expansion of certain gene families pertaining to stress resistance, oil accumulation, and polyphenol biosynthesis provides a foundational basis and will also help direct future breeding strategies. The genome sequences presented here also make *Carya* species useful models for studying the EA-ENA disjunctive distribution, and mechanisms of adaptive evolution and nutritional component accumulation in nut plants.

## Materials and Methods

### Genome sequencing and assembly

#### Plant materials

Pecan and Chinese hickory represent the only 2 commercially cultivated nut species in *Carya* (Juglandaceae). To generate high-quality reference genomes, a pecan cultivar—*C. illinoinensis cv*. Pawnee from a controlled cross “Mohawk” × “Starking Hardy Giant,” which is widely distributed across Asia and North America [29], and a Chinese hickory landrace (ZAFU-1) from the Tianmu Mountains of the Lin'an area of Hangzhou city, in Zhejiang province, China, were selected for whole-genome sequencing. Leaves of pecan and Chinese hickory, 14 other *Carya* species (10 species from the USA and 4 from Asia), and 2 *Juglans* species were also selected for whole-genome re-sequencing. Young expanding leaves from all species were harvested and stored at −80°C prior to DNA extraction. To aid protein-coding gene annotation, young leaves, epicarps, embryos, and vegetative shoots were collected from both pecan and Chinese hickory, and pistillate and staminate buds and staminate inflorescences were collected only from ZAFU-1. RNA samples were isolated using Trizol reagent (Cat. 15596-026, Invitrogen, Carlsbad, CA, USA).

#### DNA extraction and whole-genome sequencing

High molecular weight genomic DNA from Pawnee and ZAFU-1 was extracted using the CTAB method [62]. Genome sequencing was performed on Illumina Hiseq X-ten and PacBio RS II platforms for both species. For the Illumina HiSeq X-ten platform, the genomic DNA was sheared with a Bioruptor sonication device (Diagenode SA, Liège, Belgium) and a Hydroshear DNA Shearing Device (Genomic Solutions, Ann Arbor, MI, USA) for short-insert PE and large-insert MP library construction, respectively. DNA libraries of PE (250 and 500 bp) and MP (2, 5, 10, and 20 kb) were prepared and then sequenced for both species, respectively, according to the manufacturer's instructions (Illumina, San Diego, CA). SMRTbell libraries with an insert size of 20 kb were constructed after 2 iterations of DNA purification with Beckman Coulter Genomics AMPure XP magnetic beads (Danvers, MA, USA) . The genomes were sequenced on the PacBio RS II platform (PacBio) using the P6 polymerase/C4 chemistry combination, based on the manufacturer's procedure. The reads of mitochondria or chloroplasts were filtered out before we assembled the genome sequences of the 2 *Carya* species using 2 steps: (i) Illumina reads were mapped to the *J. regia* chloroplast reference genome (GenBank: MF167464.1) using BWA (v0.5.9-r16) with default parameters, and (ii) PacBio reads were mapped to the *J. regia* chloroplast reference genome using BLASR with default parameters [63].

#### Genome size estimation

The genome size of pecan and Chinese hickory was estimated using 2 methods: flow cytometry and K-mer analysis. The DNA content of DAPI (4′,6-diamidino-2-phenylindole) stained nuclei from Pawnee and ZAFU-1 was measured on a flow cytometer (Cyflow Ploidy Analyser, Partec, Munster, Germany), using *Prunus mume* as internal standard. Genome size was calculated on the basis of the following formula: (mean DNA content at G1 peak of pecan or Chinese hickory/mean DNA content at G1 peak of *P. mume*) × *P. mume* genome size (280 Mb). The 17-mer frequencies were generated using 57.80 Gb (pecan) and 47.91 Gb (Chinese hickory) high-quality PE reads (250 bp) and the genome size was estimated as described by Li et al. [64].

#### Genome assembly

##### Illumina raw data processing

PCR duplicates were removed using in-house scripts that have been posted to GigaDB [65]. Quality control involved the following steps using our scripts: (i) The PE reads were discarded when either read contained adapter sequence, >10% uncertain nucleotides, or >20% low-quality bases (Phred quality <5). (ii) MP reads that did not hit the linker were used only in support of links found with the filtered MPs but were not used to create links independently. (iii) For the TrueSeq MP data, reads were filtered out for those with low-quality bases (>50% bases with Q-value ≤8), with nucleotides >10% of the read length, and with adapter sequence. Then a total of 135.29 and 123.31 Gb (208.21-fold and 170.93-fold coverage of the estimated genomes) Illumina HiSeq X-ten clean data were used for the assembly of pecan and Chinese hickory, respectively (Additional file 1: Table S3).

##### *De novo* genome assembly using Illumina HiSeq X-ten data

Due to the high heterozygosity of the genomes (∼1.46% of pecan and ∼0.77% of Chinese hickory), we assembled the filtered clean data using Platanus (Platform for Assembling Nucleotide Sequences) (Platanus, RRID:SCR_015531) [66], a novel *de novo* sequence assembler that can reconstruct genomic sequences of highly heterozygous diploids from massively parallel shotgun sequencing data. We obtained the initial assemblies (v1.0) for both species with the following parameters: “contig (–u 0.2 –a 15 –c 26), scaffold (–u 0.2)” for pecan and “contig (–u 0.2 –a 15 –c 20), scaffold (–u 0.2)” for Chinese hickory.

##### Improving the *de novo* assemblies using PacBio data

To get the final assemblies, PBJelly v12.19.14 (PBJelly, RRID:SCR_012091) [67] and GapCloser v1.12 (GapCloser, RRID:SCR_015026) [68] were used to fill gaps in v1.0 assemblies using PacBio RSII data, ∼40× (pecan) and 30× (Chinese hickory) the estimated genomes, respectively. In brief, PBJelly began with a “Setup” process that automatically identified gaps. Any stretch of ≥25 nucleotides within a scaffold defines a gap. SMRT reads were aligned to v1.0 assemblies using BLASR (Basic Local Alignment and Serial Refinement) [63] (v5.0), which was specifically designed with the PacBio data error model in mind. The BLASR alignment information is parsed to identify gap-supporting reads. After the gap-supporting sequencing reads were identified, PBJelly assembled the reads for each gap to generate a high-quality gap-filling consensus sequence.

#### Quality evaluation of the final genome assemblies

We used 2 different datasets to evaluate the quality of the final assemblies of both species. First, the high-quality Illumina reads that were generated from short insert size PE libraries were mapped to the scaffolds using BWA mem [69]. To assess completeness of the genome assembly, the distribution of the sequencing depth at each position was calculated using SAMtools v1.6 (SAMTOOLS, RRID:SCR_002105) [70]. The guanine-cytosine content distribution was examined to analyze nucleotide distribution and assess the randomness of sequencing.

To evaluate the quality of the genome assemblies, RNA samples from young leaf tissues of both species were respectively sequenced using 250-bp libraries with PE150 on the Illumina HiSeq X-ten platform. A total of 3.17 and 2.99 Gb of transcriptomic data were assembled using Trinity v2.1.1 (Trinity, RRID:SCR_013048) [71], and generated 73,093 and 39,583 unigenes for pecan and Chinese hickory, respectively. These unigenes were then mapped to the scaffolds using BLAT (BLAT, RRID:SCR_011919) [72].

Additionally, CEGMA (CEGMA, RRID:SCR_015055) [34] (Core Eukaryotic Genes Mapping Approach) pipeline and BUSCO v3 (BUSCO, RRID:SCR_015008) [35] (Benchmarking Universal Single-Copy Orthologs) were also used to assess the completeness of the genome assemblies or annotations.

### Transcriptome sequencing

To aid the protein-coding gene annotation in both species, 4 sequencing libraries from 4 tissues (young leaves, epicarps, embryos, and vegetative shoots) of pecan and 7 tissues (young leaves, pistillate and staminate buds, staminate inflorescences, vegetative shoots, pericarps, and embryos) of Chinese hickory were constructed using the VAHTS standard mRNA-Seq Prep Kit (Vazyme Biotech, Nanjing, China) for Illumina. A total of 30.94 Gb raw data were generated for pecan and 52.06 Gb for Chinese hickory.

### Genome re-sequencing and data analysis of 16 *Carya* species and 2 *Juglans* species

Pecan and Chinese hickory, 14 other *Carya* species (10 species from the USA and 4 from Asia), and 2 *Juglans* species were also selected for whole-genome re-sequencing. Young expanding leaves from all species were harvested and stored at −80°C prior to DNA extraction. DNA from single plants was extracted using the CTAB method [63]. The 125-bp PE libraries were sequenced using Illumina NextSeq 500 technology. The data were processed for base calling, quality evaluation, removing the adapter sequence, and filtering low-quality sequences using CASAVA v1.82 (CASAVA, RRID:SCR_001802) [73] and FastQC software (FastQC, RRID:SCR_014583) [74]. The remaining clean reads were mapped to the Chinese hickory reference genome using BWA (BWA, RRID:SCR_010910) [66] (v0.5.9-r16) with the command “mem –t 4 –k 32 –M.” To reduce mismatch generated by PCR amplification before sequencing, duplicated reads were removed by the aid of SAMtools [70]. After alignment, we performed single-nucleotide polymorphism (SNP) calling on a population scale using a Bayesian approach as implemented in the package SAMtools. We then calculated genotype likelihoods from reads of each individual at each genomic location, and the allele frequencies in the sample with a Bayesian approach. To exclude SNP calling errors caused by incorrect mapping, only high-quality SNPs (coverage depth ≥3, root mean square mapping quality ≥20, maf ≥0.05, miss ≤0.1) were kept for subsequent analysis.

### Genome annotation

#### Repetitive sequences annotation

We predicted TEs in the pecan and Chinese hickory genomes by combining the *de novo*–based and the homology-based approaches. The *de novo* repeat libraries were built by using RepeatModeler v1.0.9 (RepeatModeler, RRID:SCR_015027) [75], a *de novo* repeat family identification and modeling package, for both species, separately. For the homology-based approach, we used RepeatMasker v3.3.0 (RepeatMasker, RRID:SCR_012954) [76] against the Repbase TE library, and RepeatProteinMask against the TE protein database. Tandem repeats were detected in the genomes using the software Tandem Repeats Finder (TRF) [77].

#### Identification of protein-coding genes

To predict protein-coding genes in the pecan and Chinese hickory genomes, we integrated 3 approaches—homolog-based, *de novo*, and transcriptomic aiding predictions. Homolog proteins from 10 plant genomes (*Cucumis sativus, Citrullus lanatus, Prunus persica, Malus domestica, Vitis vinifera, Glycine max, Eucalyptus grandis, Arabidopsis thaliana, Populus trichocarpa*,and *Oryza sativa*) were downloaded from Ensemble [78] and Joint Genome Institute [79]. Protein sequences from these genomes were aligned to the pecan and Chinese hickory genome assembly using TblastN, respectively, with an E-value cutoff of 1e–5. The BLAST hits were conjoined by Solar software [80]. GeneWise (GeneWise, RRID:SCR_015054) [81] was used to predict the exact gene structure of the corresponding genomic regions on each BLAST hit (Homo-set). For transcriptome-based prediction methods, RNA-sequencing reads were mapped to the assembly using TopHat v2.0.8 (TopHat, RRID:SCR_013035) [82], and Cufflinks v2.1.1 (Cufflinks, RRID:SCR_014597) [83] and then used to assemble the transcripts into gene models (Cufflinks-set). In addition, RNA-sequencing reads were assembled by Trinity v2.1.1 [71] and were also mapped to the assembly and gene models were predicted by PASA [84]. This gene set was denoted as PASA-T-set (PASA Trinity set) and was used to train *ab initio* gene prediction programs. Five *ab initio* gene prediction programs, AUGUSTUS v2.5.5 (Augustus, RRID:SCR_008417) [85], GenScan v1.0 [86], GlimmerHMM v3.0.1 (GlimmerHMM, RRID:SCR_002654) [87], Geneid (v1.3) [88], and SNAP [89], were used to predict coding regions in the repeat-masked genome. Gene model evidence from Homo-set, Cufflinks-set, PASA-T-set, and *ab initio* programs were combined by EvidenceModeler (EVM) [90] into a non-redundant set of gene structures.

#### Functional annotation of protein-coding genes

Functional annotation of protein-coding genes was achieved using BLASTP (E-value 1e–05) [91] against 2 integrated protein sequence databases: SwissProt and NCBI-nr. Protein domains were annotated by searching against the InterPro v32.0 (InterPro, RRID:SCR_006695) [92] and Pfam (v27.0) [93] databases, using InterProScan v4.8 (InterProScan, RRID:SCR_005829) [94] and HMM (v3.1) [95], respectively. The GO terms for each gene were obtained from the corresponding InterPro or Pfam entry. The pathways in which the genes might be involved were assigned by BLAST against the KEGG databases (release 53), with an E-value cutoff of 1e–05.

#### Annotating non-coding RNAs

Noncoding RNA genes, including rRNAs, tRNAs, and snRNAs, were predicted in the assemblies. The tRNA genes were identified by tRNAscan-SE [96] software with the eukaryote parameters. The rRNA fragments were predicted by aligning to *Arabidopsis* and rice template rRNA sequences using BLASTn at an E-value of 1e–10. The microRNA and snRNA genes were predicted by searching against the Rfam database (release 9.1) [97] using INFERNAL software (Infernal, RRID:SCR_011809) [98].

### Evolutionary analyses of the genomes and *Carya*

#### Phylogenetic analysis

Except for pecan and Chinese hickory, *V. vinifera* and 10 other genome-sequenced representatives from the Rosids (*J. regia, G. max, Medicago truncatula, P. persica, Morus notabilis, Carica papaya, Gossypium hirsutum, Theobroma cacao, Betula pendula*, and *Populus trichocarpa*), along with *A. thaliana*, were selected for constructing the phylogenetic tree (*J. regia* genome data were downloaded from [99]; others were downloaded from Phytozome [100] [v12]). The protein set of each species was obtained and filtered as follows: (i) only the longest isoform was considered for further analysis if a gene encoded several isoforms; (ii) proteins of <30 amino acids were filtered out. The similarity relation between homologous proteins in all species was obtained through BLASTp with the E-value 1e–5. All the protein datasets of the 14 species were clustered into paralogous and orthologous using the program OrthoMCL [101] with the inflation parameter 1.5. Finally, 170 single-copy-gene–encoded proteins were used for the phylogenetic analysis. The protein sequences from all species were then aligned by MUSCLE (MUSCLE, RRID:SCR_011812) [102] and a super alignment matrix was generated by combining all the alignment results. A phylogenetic tree containing 14 species was constructed using RAxML (RAxML, RRID:SCR_006086) [103] with the ML method and 1,000 bootstraps. Finally, the MCMCtree program implemented in phylogenetic analysis by maximum likelihood (PAML) (PAML, RRID:SCR_014932) [104] was applied to infer the divergence time on the basis of the phylogenetic tree. The MCMCtree running parameters were as follows: burn-in, 5,000,000; sample-number, 1,000,000; sample-frequency, 50. The calibration times of divergence between *A. thaliana* and *C. papaya* (54–90 MYA), *G. hirsutum* and *T. cacao* (32–99 MYA), *A. thaliana* and *P. trichocarpa* (107–109 MYA), *G. max* and *M. truncatula* (46–60 MYA), *M. notabilis* and *P. persica* (73–90 MYA), and *A. thaliana* and *G. max* (107–111 MYA) were obtained from the TimeTree database [105].

#### Comparative genomes

The χ^2^ test, as one of the widely used hypothesis test methods, was used to test the expansion and contraction of gene families in both pecan and Chinese hickory. The gene number of gene families was compared among Rosales (*P. persica* and *M. notabilis*), Fabales (*G. max* and *M. truncatula*), and Fagales (English walnut, pecan, and Chinese hickory). Pecan, Chinese hickory, and English walnut (*J. regia*) were further compared by gene number of gene families. Gene families were considered expanded if the number of genes in 1 species was significantly (*P*< 0.05) more than that in other species by χ^2^ test.

#### Distribution of hickories and phylogenetic reconstruction of *Carya*

The geographical distribution (e.g., longitude, latitude, altitude, habitat) for extant *Carya* species in EA was obtained from Chinese Virtual Herbarium [106], which provides online access to herbarium specimens and botanical information chiefly constructed by the Institute of Botany, Chinese Academy of Sciences, and partially from National Specimen information infrastructure [107], another online sharing platform of teaching samples. Similarly, the information of extant species in North America was obtained from the Natural Resources Conservation Service [108]. The distribution of the extinct hickories was retrieved from the literature [3]. Finally, a distribution map of all the extant and extinct species was generated by means of on-screen digitization and visual interpretation techniques using ArcGIS 10.2 software [109].

To estimate the phylogenetic relationship between species in *Carya*, the 125-bp PE reads in pecan and Chinese hickory, as well as the re-sequencing data of another 14 *Carya* species and 2 *Juglans* out-group species were re-sequenced using Illumina NextSeq 500. The raw data were processed for base calling, quality evaluation, removing the adapter sequence, and low-quality sequence using CASAVA (v1.82) and FastQC software, with the following steps: (i) removing reads with ≥10% unidentified nucleotides; (ii) removing reads with >20% bases having Phred quality <5; (iii) removing reads with >10 nucleotides aligned to the adapter, allowing ≤10% mismatches; and (iv) removing putative PCR duplicates generated by PCR amplification during the library construction process (i.e., read 1 and 2 of 2 PE reads that were completely identical).

The remaining high-quality PE reads were mapped to the ZAFU-1 genome using BWA (Burrows-Wheeler Aligner) (v0.7.8) with the command “mem –t 4 –k 32 –M.” After alignment, SNP calling on a population scale was performed using a Bayesian approach as implemented in the package SAMtools (v1.4). Genotype likelihoods were calculated with a Bayesian approach from reads for each individual at each genomic location and the allele frequencies in the sample. To exclude SNP calling errors caused by incorrect mapping, only high-quality SNPs (coverage depth ≥3, root mean square mapping quality ≥20, maf ≥0.05, miss ≤0.1) were used for further analysis.

To clarify the phylogenetic relationship from a genome-wide perspective, an individual-based neighbor-joining tree was constructed with 1,000 bootstraps using the software TreeBestv1.9.2 [110]. The MCMCtree program implemented in PAML was applied to infer the divergence time on the basis of the phylogenetic tree. The MCMCtree running parameters were as follows: model: JC69, burnin: 10,000, nsample: 100,000, sampfreq: 2.

#### Whole-genome duplication

To identify syntenic blocks, the protein sequences from pecan, Chinese hickory, and English walnut [36] were searched against themselves using BLASTp (E < 1e–5). The results were subjected to MCScan [111] (–a, –e:1e–5, –u:1, –s:5) to determine syntenic blocks. At least 5 genes were required to define a synteny. We calculated the 4DTv distribution for each gene pair from the aligned blocks to estimate the speciation or WGD event that occurred during the evolutionary history of the 2 hickories. The 4DTv analysis and WGD divergence time were estimated as described by The Potato Genome Sequencing Consortium [112].

#### Insertion time estimate of LTRs

LTRharvest [113] and LTRfinder were used to predict LTR-RTs with the following parameters: LTR length of 100–5,000 base pairs (bp), LTR interspace length of 1,000–20,000 bp. The tRNAscan-SE was used for predicting tRNA sequences and LTRdigest [114] was used for structure annotation (e.g., PBS, PPT, protein) of LTR-RTs with optimal annotation. LTR-RTs were clustered by USEARCH software with the similarity parameter of 70%. The LTR-RTs with copy number >2 or single copy containing protein domains were recruited. After that, the nucleotide variations (λ) in the 5′ and 3′ terminals of intact LTR-RTs were estimated by MUSCLE [102]. If λ was >0.75, the intact LTR-RT would be considered invalid. For those valid intact LTR-RTs, the genetic distances (*K*) were calculated by *K* = −0.75 ln(1 – 4λ/3). Finally, the insertion time of LTR-RTs was calculated based on the following formula: *T* = *K*/2*r* (*r* = 1.3 × 10^–8^ per site and per year), and distributions were further plotted.

### Identification and phylogenetic analysis of selected genes

The protein sequences related to stress response, oil accumulation, and antioxidants in *A. thaliana* were downloaded from NCBI. Then using *Arabidopsis* homologs as query, we identified the candidates in pecan and Chinese hickory by BLASTp with best hit. If these genes were in a common family in OrthoMCL or the E-value <1e–20, candidate genes were predicted by Pfam [93]. Only candidate genes with the same protein domain were selected. All the amino acid sequences were aligned using ClustalW implemented in the MEGA v7.0 software [115]^.^ The phylogenetic trees were generated with MEGA, using the ML method based on the Jones-Taylor-Thornton matrix model, with 1,000 bootstrap replications each. The genes in a phylogenetic tree were further classified to several subfamilies according to intrinsic domains or referral to the phylogenetic tree in *Arabidopsis*. Gene structure was plotted according to its CDS and domain using GSDS software [116].

### Transcriptome analysis during embryo development

Raw transcriptomic data representing 3 key stages (i.e., the early and fully extended stages of cotyledon development, and the fully matured stage of the embryos) during embryo development in 2 pecan trees as biological replicates were deposited in the NCBI database (SRR6793957, SRR6793955, and SRR6793961 for replicate 1; SRR6793958, SRR6793956, and SRR6793962 for replicate 2). Raw transcriptomic data representing 3 key stages during embryo development in Chinese hickory were deposited or downloaded from the NCBI database. For each stage, samples that were collected in the same season of years 2012 (SRR6785066, SRR2006624, and SRR2006626) and 2013 (SRR6785065, SRR2006629, and SRR2006631) were treated as 2 biological replicates in Chinese hickory. To link the genome features and the transcriptomic responses, we analyzed or reanalyzed the data based on our assemblies and annotations. Briefly, high-quality filtered reads were mapped to the draft reference genomes with SOAP aligner (Soap2.21) [117] (mismatches > 2 bases). The expression level (FPKM value) for each protein-coding gene was calculated by Cufflinks [83] using default parameters. Genes with FPKM > 0.5 were defined as expressed. For those genes with >1 transcript, the longest was used to calculate expression level and coverage for each gene. DESeq2 [118] were used for normalizing gene expression (BaseMean) in each sample, and identified DEGs for each compared group by using “*P*-adj (adjusted *P*-value) < 0.05 and the |log_2_Ratio| > 1” as the threshold. The DEGs were further grouped into 8 clusters on the basis of their temporal expression patterns.

To obtain the significantly enriched GO term for DEGs, all DEGs were mapped to GO terms in the GO database [119]. GO enrichment analysis of differentially expressed genes was implemented by the GOseq R package [120], in which gene length bias was corrected. GO terms with adjusted *P*-value < 0.05 were considered significantly enriched by differentially expressed genes, and labeled with asterisks. The significantly enriched GO terms were selected using a hyper-geometric test to develop hierarchical clusters of a sample tree by Euclidean distance. To further clarify the biological functions of DEGs, a pathway-based analysis was conducted using the public KEGG pathway-related database [121]. We used KOBAS software to test the statistical enrichment of differential expression genes in KEGG Pathways [122]. Pathways with Q-value < 0.05 were considered to be significantly enriched. We drew the heat map of expression levels using pheatmap [123] for the selected genes that we were interested in.

## Availability of supporting data and materials

The raw data of genomes, RNA-seq, and re-sequencing (Bioproject ID PRJNA435846) are available in GenBank of the NCBI. Assemblies, annotations, and other supporting data are also available in the *GigaScience* database, GigaDB [65].

## Additional files

**Additional file 1:** A Word file with Tables S1-S25.

**Additional file 2:** A Word file with Fig. S1–S14.

**Additional file 3:** An Excel file with DEGs.

GIGA-D-18-00185_Original_Submission.pdf

GIGA-D-18-00185_Revision_1.pdf

GIGA-D-18-00185_Revision_2.pdf

Response_to_Reviewer_Comments_Original_Submission.pdf

Response_to_Reviewer_Comments_Revision_1.pdf

Reviewer_1_Report_Original_Submission -- Meg Staton 6/19/2018 Reviewed

Reviewer_1_Report_Revision_1 -- Meg Staton 11/21/2018 Reviewed

Reviewer_2_Report_Original_Submission -- Jarkko Salojarvi, DSc (tech) 6/23/2018 Reviewed

Supplemental Files

## Abbreviations

4DTv: 4-fold degenerate synonymous sites of the third codon position values; ABA: abscisic acid; ACCase: acetyl coenzyme A carboxylase; BLASR: Basic Local Alignment and Serial Refinement; bp: base pairs; CHS: chalcone synthase; CTAB: cetyltrimethylammonium bromide; DEG: differentially expressed gene; EA: East Asia; ENA: eastern North America; FAD: fatty acid desaturase; Gb: gigabase; GO: gene ontology; kb: kilobases; KEGG: Kyoto Encyclopedia of Genes and Genomes; LEA: late embryo abundant; LAR: leucoanthocyanidin reductase; LTR: long terminal repeat; ML: maximum likelihood; MP: mate pair; MYA: million years ago; NCBI: National Center for Biotechnology Information; PacBio: Pacific Biosciences; PAML: phylogenetic analysis by maximum likelihood; PCR: polymerase chain reaction; PE: paired end; SAD: ∆-9-stearoyl-ACP desaturase; snRNA: small nuclear RNA; SMRT: single-molecule real-time; SNP: single-nucleotide polymorphism; TAG: triacylglyceride; TE: transposable element; TRF: Tandem Repeats Finder; tRNA: transfer RNA; WGD: whole-genome duplication.

## Competing interests

The authors declare that they have no competing interests.

## Funding

This research was mainly supported by a grant from the National Key R&D Program of China (2018YFD1000604), and partially supported by a grant of the 863 Program from the Chinese Ministry of Science and Technology (2013AA102605), a grant from National Science Foundation of China (31470682, 31670682, and 31570666), the Zhejiang Agriculture (fruit) New Variety Breeding Major Science and Technology Special (2016C02052-13), and the Teacher Professional Development Project of Domestic Visiting Scholar in Zhejiang Province (FX2015040).

## Authors’ contributions

J.H. and L.X. designed the experiments and managed the project. L.X. conceived the paper and Y.H and L.X. wrote the manuscript. Z.W., L.J.G., R.Z., Q.Z., X.W., G.X., C.X., C.H., R.H., T.F., J.W., C.S., and S.Z. collected samples. Y.H. and Z.W. prepared and purified DNA and RNA samples for whole-genome sequencing and RNA sequencing. C.X. estimated genome sizes by flow cytometry analysis. Y.H., L.X., Z.Z., R.Z., and S.Z performed data analyses. L.X., Y.H., and Z.Z. prepared and edited all the figures and tables. J.H., L.X., Y.H, L.J.G., X.W., C.L., and J.R. revised the manuscript and L.J.G., C.L., X.W., J.R., and L.X. polished the language. M.C., Z.C., L.G., and W.J. provided valuable suggestions on the beginning of the project initiation. J.H., Z.W., B.Z., and J.W. initiated and coordinated the project. All authors discussed the results and commented on the manuscript.
