# Supplementary material for: The genomes of pecan and Chinese hickory provide insights into Carya evolution and nut nutrition
Source: Gigascience. 2019 May 2;8(5):giz036. doi: 10.1093/gigascience/giz036 (PMC6497033; doi:10.1093/gigascience/giz036)
Supplement: GIGA-D-18-00185_Revision_2.pdf [file giz036_giga-d-18-00185_revision_2.pdf]

GigaScience

The genomes of pecan and Chinese hickory provide insights into Carya evolution and nut nutrition

--Manuscript Draft--

**Manuscript Number:**	GIGA-D-18-00185R2
**Full Title:**	The genomes of pecan and Chinese hickory provide insights into Carya evolution and nut nutrition
**Article Type:**	Research
**Funding Information:**	
**Abstract:**	Background: Pecan (Carya illinoinensis) and Chinese hickory (C. cathayensis) are important commercially cultivated nut trees in genus Carya (Juglandaceae), with high nutritional value and significant health benefits.<br>Results: We obtained more than 187.22 Gb and 178.87 Gb sequences, ~288x and 248x genome coverage, to a pecan cultivar ('Pawnee') and a domesticated Chinese hickory landrace (ZAFU-1). The total assembly is 651.31 Mb for pecan and 706.43 Mb for Chinese hickory. Two genome duplication events before the divergence from walnut were found in these species. Gene family analysis highlighted key genes in biotic and abiotic tolerance, oil, polyphenols, essential amino acids and B vitamins. Further analyses of reduced-coverage genome sequences of 16 Carya and 2 Juglans species provide additional phylogenetic perspective on crop wild relatives.<br>Conclusions: Cooperative characterization of these valuable resources provides a window to their evolutionary development, and a valuable foundation for future crop improvement.
**Corresponding Author:**	Lihong Xiao, Ph.D.<br>Zhejiang A&F University<br>CHINA
**Corresponding Author Secondary Information:**	
**Corresponding Author's Institution:**	Zhejiang A&F University
**Corresponding Author's Secondary Institution:**	
**First Author:**	Youjun Huang, Ph.D.
**First Author Secondary Information:**	
**Order of Authors:**	Youjun Huang, Ph.D.
	Lihong Xiao
	Rui Zhang
	Zhengjia Wang
	Zhongren Zhang
	Chunying Huang
	Ren Huang
	Yumeng Luan
	Tongqiang Fan
	Jianhua Wang
	Chen Shen
	Shenmei Zhang
	Xinwang Wang

Powered by Editorial Manager® and ProduXion Manager® from Aries Systems Corporation

	Jennifer Randall
	Bingsong Zheng
	Jiasheng Wu
	Qixiang Zhang
	Guohua Xia
	Chuanmei Xu
	Ming Chen
	Liangsheng Zhang
	Wenkai Jiang
	Lizhi Gao
	Zhiduan Chen
	Charles A. Leslie
	L. J. Grauke
	Jianqin Huang
**Order of Authors Secondary Information:**	
**Response to Reviewers:**	Dear Professor, Dr. Laurie Goodman, Editor-in-Chief of GigaScience:<br>Thank you for accepting our manuscript (GIGA-D-18-00185) for publication in GigaScience, contingent on approval of minor revisions. We also thank the reviewers for their valuable comments and suggestions for improving our manuscript. We have revised the document fully based on reviewers' comments and suggestions (shown as red text in the main document and in additional supplementary files). In the cover letter, we provide responses to the reviewers' comments and suggestions point by point.<br>In response to reviewer #1's comments, we also checked the manuscripts and additional files carefully and made additional revisions on some typos. The language has been polished by collaborators, who are native English speakers.<br>We hope the revisions incorporated here allow the manuscript to be accepted for publication in GigaScience.<br><br>With Best Regards<br><br>Yours Sincerely<br><br>Corresponding authors<br>Drs. Jianqin Huang, Lihong Xiao and L.J. Grauke<br><br>The following pages are the responses to the reviewers' comments and suggestions.<br><br>Responses to the reviewers' comments and suggestions

Powered by Editorial Manager® and ProduXion Manager® from Aries Systems Corporation

Responses to Reviewer #1

We are grateful to reviewer #1 for valuable comments and suggestions and have given full consideration to all of them.

Our responses to the reviewer's numbered comments are in blue text following the symbol ●.

#3. The authors did not correctly interpret my original comment for the rRNA gene counts. I am not questioning their method of identifying the genes, I am questioning if the assembly is correct and able to represent the true number of rRNA genes. Large tandem arrays, similar to other types of repetitive elements, are difficult to assemble. Likely many copies of the genes are collapsed into a single consensus sequence, which you can detect by looking at sequence coverage in the region. This makes it difficult or impossible to compare rRNA gene copy number between the two species. However, the text in the results highlighting this difference is gone, so I think it is now fine. I do not request a revision, just want to clarify my original comments.

● We thank reviewer #1 for raising the issue of accuracy in rRNA gene counts and are pleased that our last response was satisfactory.

#4. The response to reviewers indicates "mitochondria and chloroplast genomes… we have filtered out the reads and obtained the chloroplast genome sequences and annotations before scaffolding". This filtering needs to be included in the methods. The authors also state that "we didn't include the chloroplast genomes in this manuscript" but the manuscript additional file list includes "Additional file 4: An excel file with chloroplast sequences of pecan and Chinese hickory". However, I can't download that file, so I'm not sure it was submitted. This is an important a decision - if the chloroplast is a part of this paper, it needs to be included in the methods and the results and it needs to be submitted to a public repo, preferably with the assembly in NCBI. If you do not want to include it in this manuscript, then it needs to be removed from the supplementary materials, but the methods should still mention these reads were filtered out.

● As mentioned in our last responses: 'We have filtered out the reads and obtained the chloroplast genome sequences and annotations before scaffolding. But unfortunately, these assemblies and annotations showed significant difference with our assemblies from other two varieties of pecan (unpublished data). To confirm if or not the differences are true will need extensive experiments and more time. Therefore, we didn't include the chloroplast genomes in this manuscript.' Therefore, we don't intend to include the organelle genomes in the current manuscript and they have been removed from supplementary materials.

Chloroplast reads were filtered out before we assembled the genome sequences of the two Carya species using two steps as follows: (1) Illumina reads were mapped to Juglans regia chloroplast reference genome (GenBank: MF167464.1) using BWA (v0.5.9-r16) with default parameters; (2) PacBio reads were mapped to J. regia chloroplast reference genome using BLASR with default parameters. The methods were added in Section '1.2 DNA extraction and whole genome sequencing' (Please see Page 25, Line 6-10).

#9. Expression analysis. The reviewers did add this to the results section, which is great. However, I still find it unclear. The text refers to up and down regulated genes, but I'm not clear on if they were up regulated in various individual time points in comparison to other time points or in one species versus the other or both. Scripts that were used to run DESeq2 or at least the statistical model used would be nice to include in the methods. For example, if the model was ~time within each species, that would report any transcript responding to time. With three time points, that is a bit more complicated than up and down (ie, could very high in time point 1, low in time point 2, moderate in time point 3). Or if contrasts were performed between individual time points (upregulated in time point 2 in comparison to time point 1). This doesn't need to be a focus point of the paper, but I do think its imporant to be clear about what was done and how it was done.

● We thank reviewer #1 for raising this question. We conducted transcriptomic analysis during embryo development and identified differentially expressed genes (DEGs) in pecan and Chinese hickory, respectively. Levels of DEGs significantly vary among three developmental stages (i.e., the early and fully extended stages of cotyledon development, and the fully matured stage of the embryos, designated as PEY1-3 in pecan and HEY1-3 in Chinese hickory). In detail, the DEGs were identified from PEY2

	vs PEY1, and PEY3 vs PEY2 in pecan and from HEY2 vs HEY1, and HEY3 vs HEY2 in Chinese hickory. The number and the result of GO enrichment of these DEGs were added in this revised manuscript (see Page 15, Line 3 to Line 22) and in additional file 2 (see Fig. S9-S12).<br><br>#8 page 12 lines 14-15. "The WGD time of walnut was previously estimate at 60Mya and that the estimates diverge a bit" needs to be reworded. Yes, I put that latter phrase in my original review, but it was a comment that I didn't expect the authors to use in the paper. I do not believe it makes sense as used in the paper or has enough precision to appear in the results of a scientific paper. This sentence also has some grammatical errors.<br>● We apologized for misunderstood your question. We carefully read your original review and the article of walnut genome and related references therein (Martinez-Garcia et al., 2016). We found that the WGD of Juglans lineage (~66 Myr ago) in the walnut genome paper that published in the Plant Journal (Martinez-Garcia et al 2016) were estimated based on fossils record. Moreover, Zhang et al. (2013) used fossils of Carya that are clearly distinguished from those of Juglans based on morphology, that were recovered from particular geological strata that are dated using internationally recognized chronology, to estimate dates of origin for the genus. Their time estimate is based on fossils of the two genera that are distinct and found in strata that are dated with internationally recognized chronology. The 4DTv distance in our manuscript (~38.9 Myr ago) is calibrated using methods reported for potato. These results lead us to consider that estimate of 66 Ma for separation of Carya from Juglans is more reliable. Therefore, we re-calibrated the WGD events in Carya species (Fig. 1d) by using 66 Myr as a comparator, and corrected the date in the phylogenetic trees of species in Fig. 1a and Fig. 1b. Meanwhile, we also revised the description in the manuscript accordingly, see Page 11, Line 17-18; Page 12, Line 16 and Line 18.<br>Additional revisions:<br>● To present our results more accurately, we revised several typos in the last version of our manuscript, which were remarked in the main text and several were listed as below:<br>1. In additional file 1: Table S1, “insertsieze” corrected as “Insert size”<br>2. In additional file 1: Table S2, “sampling ate” corrected as “Sampling date”<br>3. In additional file 1: Table S3, we added two rows “Total clean data (Gb)” and “Sequence coverage of clean data (X) ”. Correspondingly, we revised the number in abstract of page 4, lines 16-17 and line 22 in page 8, lines 1 in page 9, line 17 in page 25, line 6 in page 26 and line 3-4 in page 28.<br>4. We deleted a redundant Bioproect ID PRJNA427736 in “Availability of data and materials” in Page 36.<br>5. We corrected a typo “clariefy” to “clarify” in page 12.<br>6. We added a grant in “Funding” section in page 37, which we missed in the original manuscript.
**Additional Information:**	
**Question**	**Response**
Are you submitting this manuscript to a special series or article collection?	No
**Experimental design and statistics**<br><br>Full details of the experimental design and statistical methods used should be given in the Methods section, as detailed in our Minimum Standards Reporting Checklist. Information essential to interpreting the data presented should be made available in the figure legends.	Yes

Have you included all the information requested in your manuscript?	
**Resources**<br><br>A description of all resources used, including antibodies, cell lines, animals and software tools, with enough information to allow them to be uniquely identified, should be included in the Methods section. Authors are strongly encouraged to cite Research Resource Identifiers (RRIDs) for antibodies, model organisms and tools, where possible.<br><br>Have you included the information requested as detailed in our Minimum Standards Reporting Checklist?	Yes
**Availability of data and materials**<br><br>All datasets and code on which the conclusions of the paper rely must be either included in your submission or deposited in publicly available repositories (where available and ethically appropriate), referencing such data using a unique identifier in the references and in the “Availability of Data and Materials” section of your manuscript.<br><br>Have you have met the above requirement as detailed in our Minimum Standards Reporting Checklist?	Yes

Manuscript

Click here to access/download;Manuscript;Manuscript-20190130_SCEcomments-LX.docx

Click here to view linked References

Title: The genomes of pecan and Chinese hickory provide insights into *Carya* evolution and nut nutrition

Authors: Youjun Huang[1†], Lihong Xiao[1†*], Zhongren Zhang[2†], Rui Zhang[1], Zhengjia Wang[1], Chunying Huang[1], Ren Huang[1], Yumeng Luan[1], Tongqiang Fan[1], Jianhua Wang[1], Chen Shen[1], Shenmei Zhang[1], Xinwang Wang[3], Jennifer Randall[4], Bingsong Zheng[1], Jiasheng Wu[1], Qixiang Zhang[1], Guohua Xia[1], Chuanmei Xu[1], Ming Chen[5], Liangsheng Zhang[6], Wenkai Jiang[2], Lizhi Gao[7], Zhiduan Chen[8], Charles A. Leslie[9], L. J. Grauke[3], Jianqin Huang[1*]

Affiliations:

[1] State Key Laboratory of Subtropical Silviculture, Zhejiang A&F University, Hangzhou 311300, China

[2] Novogene Bioinformatics Institute, Beijing 100083, China

[3] Pecan Breeding and Genetics, Agricultural Research Service, United States Department of Agriculture, Somerville, TX 77979, USA

[4] College of Agricultural, Consumer, and Environmental Sciences, New Mexico State University, Las Cruces, NM 88003, USA

[5] School of Life Science, Zhejiang University, Hangzhou 310058, China

[6] Haixia Institute of Science and Technology, Fujian Agriculture and Forestry University, Fuzhou 350002, China

[7] Plant Germplasm and Genomics Center, Germplasm Bank of Wild Species in Southwestern China, Kunming Institute of Botany, Chinese Academy of Sciences, Kunming 650201, China

[8] State Key Laboratory of Systematic and Evolutionary Botany, Institute of Botany, Chinese Academy of Science, Beijing 100093, China

[9] Department of Plant Sciences, University of California, Davis, CA 95616, USA

[†] Contribution equally in manuscript preparation.

[*] To whom correspondence should be addressed:

Jianqin Huang, Ph.D.

Tel: +86 571 63740859

E-mail: huangjq@zafu.edu.cn

ORCID: 0000-0002-3901-0447

Lihong Xiao, Ph.D.

Tel: +86 0 17826874256

Email: xiaolh@zafu.edu.cn

ORCID: 0000-0003-4864-6874

Youjun Huang: hyj@zafu.edu.cn

Lihong Xiao: xiaolh@zafu.edu.cn

Rui Zhang: rui.zhang@zafu.edu.cn

Zhengjia Wang: wzhj21@163.com

Zhongren Zhang: zhangzhongren08@126.com

Chunying Huang: 307970537@qq.com

Ren Huang: 457544962@qq.com

Yumeng Luan: 351677410@qq.com

Tongqiang Fan: blackeyesftq@gmail.com

Jianhua Wang: 690042053@qq.com

Chen Shen: 893194219@qq.com

Shenmei Zhang: 250422958@qq.com

Xinwang Wang: Xinwang.Wang@ars.usda.gov

Jennifer Randall: jrandall@nmsu.edu

Bingsong Zheng: bszheng@zafu.edu.cn

Jiasheng Wu: wujs@zafu.edu.cn

Qixiang Zhang: qxzhang@zafu.edu.cn

Guohua Xia: zjfc_ghxia@126.com

Chuanmei Xu: Xuchuanmei1979@126.com

Ming Chen: mchen@zju.edu.cn

Liangsheng Zhang: fafuzhang@163.com

Wenkai Jiang: jiangwenkai@novogene.com

Lizhi Gao: lgao@mail.kib.ac.cn

Zhiduan Chen: zhiduan@ibcas.ac.cn

Charles A. Leslie: caleslie@ucdavis.edu

L. J. Grauke: lj.grauke@ars.usda.gov

Jianqin Huang: huangjq@zafu.edu.cn

Abstract

Background: Pecan (*Carya illinoinensis*) and Chinese hickory (*C. cathayensis*) are important commercially cultivated nut trees in the genus *Carya* (Juglandaceae), with high nutritional value and significant health benefits.

Results: We obtained more than 187.22 Gb and 178.87 Gb of sequence, and ~288x and 248x genome coverage, to a pecan cultivar ('Pawnee') and a domesticated Chinese hickory landrace (ZAFU-1). The total assembly size is 651.31 Mb for pecan and 706.43 Mb for Chinese hickory. Two genome duplication events before the divergence from walnut were found in these species. Gene family analysis highlighted key genes in biotic and abiotic tolerance, oil, polyphenols, essential amino acids and B vitamins. Further analyses of reduced-coverage genome sequences of 16 *Carya* and 2 *Juglans* species provide additional phylogenetic perspective on crop wild relatives.

Conclusions: Cooperative characterization of these valuable resources provides a window to their evolutionary development, and a valuable foundation for future crop improvement.

Keywords: *Carya*, pecan, Chinese hickory, whole genome sequence, adaptive evolution, nutritional value, genetic improvement

Background

Juglandaceae contains ca. 60 known species [1], including many internationally important nut crops such as Persian walnut (*Juglans regia*), pecan (*Carya illinoinensis*) and Chinese hickory (*C. cathayensis*) as well as valuable hardwood species such as Black walnut (*J. nigra*). The genus *Carya* consists of ca. 20 species worldwide [2, 3] with an intercontinentally disjunctive distribution between East Asia (EA) and eastern North America (ENA) [3, 4]. Pecan and Chinese hickory are the representatives in ENA and EA, respectively, and the only two commercially cultivated nut trees of the genus [5, 6]. The nut consumption of pecan and Chinese hickory is dramatically increasing in recent years, due to their high nutritional value and important health benefits. In comparison to most other nuts, pecan and Chinese hickory contain high quantities of healthy mono-unsaturated fatty acids and a high level of antioxidants with an array of phytochemicals such as phenolic compounds [7, 8]. The nuts are also a rich source of dietary fiber, protein, minerals and B vitamins – especially thiamine [9]. Recent studies highlight the health benefits of these nuts in conjunction with reduction of multiple diseases such as tumor, edematogeny, hyperglycemia and hyperlipidemia [10-12]. These beneficial properties have promoted wide cultivation of these species. In the U.S., pecan annual yields exceed 130,000 tons with a value over $600 (USD) million annually [13]. In China, Chinese hickory provides annual production of close to 30,000 tons with a farm gate value over $ 125 million (USD) per year before the year 2010 [14]. Annual production exceeded 40,000 tons according to statistics of State administration of Forestry and Grassland (unpublished data) in 2017.

In the United States and Mexico, wild pecans were native along the river bottomlands with a wide variance in climate between 30 and 42°N latitude [13]. The natural habitat of pecan ranges from mild to harsh winters and from humid to semi-arid climates with the preference for loamy, well-drained

first-class river bottom land [15]. Although wild pecans were well known and considered a delicacy among native and colonial Americans, commercial production of pecans in the United States did not begin until the 1880s [16]. The pecan research activities of the United States Department of Agriculture (USDA), date to the same time period [17]. The USDA National Collection of Genetic Resources for Pecans and Hickories (NCGR-*Carya*) has collected and maintains over 400 pecan cultivars, from 25 US states and Mexico. Some of the cultivars are widely planted worldwide [5]. In 2016, global production of pecan was primarily from Mexico (47%) and the United States (46%), and the rest (7%) from other countries including Australia (4%), China (1%), South Africa (1%) and South America (1%) [18].

Chinese hickory is a specialty of the Hangzhou area Zhejiang province in China, where it has been cultivated for consumption for over 500 years since the Ming Dynasty. Both wild and domesticated Chinese hickories grow only in moist valleys at the foothill of the Tianmu Mountains at an elevation of 500-1200 meters within the Zhejiang and Anhui provinces in China. In this climatic location they receive full sun in sheltered locations [19]. However, unlike pecan, Chinese hickory has naked terminal buds, making it less adaptable to colder climates [5, 20]. In addition, Chinese hickory has smaller nuts with harder shells and the trees lack tolerance to abiotic stresses such as heat, flooding, drought and salinity [18], which significantly restrict its commercial cultivation worldwide. Breeding of Chinese hickory is far behind pecan and plateaued at domestication levels until the past decade. However, the species has nucellar embryony (apomixis) that would be valuable for passing down the disease-resistant trait in production [21].

In pecan and Chinese hickory breeding programs, the mission is to preserve, evaluate and enhance genetic resources and to develop superior cultivars with high disease/insect/(a)biotic resistance and

excellent nut quality [22, 23]. To date, several superior cultivars in pecan and Chinese hickory are available with desirable traits such as precocity, high-yield, disease and stress resistance, high nut and kernel quality, etc. [23, 24]. On average, over 20 years are required to release a new cultivar by conventional breeding due to extended periods of juvenility [25, 26]. The rapid development of modern biotechnologies, such as genome sequence-based whole genome associated analysis and gene editing, may make it possible to shorten the selection process [27].

To develop genetic tools for acceleration of nut tree improvement in *Carya*, whole genome sequencing of reference genomes was initiated [28], identifying a popular pecan cultivar ‘Pawnee’ [27, 29] for its international value as a base in breeding efforts [30]. Here, we report the completed sequence of two *Carya* genotypes: ‘Pawnee’ and a widely planted representative (ZAFU-1) of Chinese hickory. Tissue samples were collected from a single tree of each. We also re-sequenced 16 *Carya* species (including pecan and Chinese hickory) from EA and ENA, and two *Juglans* species (out-group). A hybrid assembly strategy delivered high-quality draft genomes for the *Carya* species. Global analyses of genome features, along with the re-sequencing data in 16 *Carya* species and full assessments of expression changes during embryo development provide valuable insight into the evolution of the two genomes, disjunctive distribution of the genus, their high degree of adaptation to biotic or abiotic stresses, and the accumulation of oils, polyphenols, essential amino acids and B vitamins. These analyses provide a solid foundation for future studies on improvements of abiotic and biotic stress tolerance, yield and nutrition in hickories, and offers great potential for genome-based breeding of superior cultivars in the genus *Carya*, with the aid of the established explant regeneration techniques [31, 32], and the advanced CRISPR-Cas9 gene editing techniques [33].

Data Description

To obtain the whole genome sequences of pecan and Chinese hickory genomes, genomic DNA was extracted from leaf tissues of the pecan cultivar 'Pawnee' (*Carya illinoinensis*, NCBI:txid32201) and the domesticated Chinese hickory landrace ZAFU-1 (*C. cathayensis*, NCBI:txid139927) using the cetyltrimethylammonium bromide (CTAB) method. Paired-end libraries with insert sizes ranging from 250 bp to 500 bp and mate pair libraries with insert sizes of 2 kb and 20 kb were constructed according to the manufacturer's instructions (Illumina, San Diego, CA). All constructed libraries were sequenced on Illumina HiSeq X-ten. Single-molecule real-time (SMRT) sequencing of long reads on a PacBio RS II platform (Pacific Biosciences, USA) was used to assist the subsequent *de novo* genome assembly process. First, a 20-kb insert size SMRTbell library was prepared following the manufacturer's protocol (PacBio, CA, USA). Then, these libraries were sequenced on PacBio RS II platform (Pacific Biosciences, USA) using the P6 polymerase/C4 chemistry combination, based on the manufacturer's procedure (Pacific Biosciences, CA, USA). Sequencing statistics for all libraries are outlined in Additional file 1: Table S1. In total, about 157 Gb and 161 Gb reads were generated on Illumina platforms, and 21.72 Gb and 25.80 Gb reads were generated on PacBio platforms of ZAFU-1 and 'Pawnee'. Quality control involved the following steps: (1) removing reads with >= 10% unidentified nucleotides (N); (2) removing reads with > 20% bases having Phred quality < 5; (3) removing reads with >10 nt aligned to the adapter, allowing <= 10% mismatches; (4) removing putative PCR duplicates generated by PCR amplification during the library construction process (i.e. read 1 and 2 of two paired-end reads that were completely identical). Finally, about 135.29 Gb and 123.31 Gb of Illumina clean data and 25.75 Gb and 21.68 Gb PacBio clean data were obtained for

the *de novo* assemblies of the 'Pawnee' and ZAFU-1 genome, respectively.

The details about sample collection, library construction, sequencing, assembly, gene prediction, and annotation can be found in the Materials and Methods.

Results

Genome sequencing, assembly and quality assessment

To obtain high-quality reference genome sequences, we sequenced the genomes of 'Pawnee' and ZAFU-1 (Additional file 1: Table S2) using HiSeq X-Ten sequencing platform from Illumina and single-molecule real-time (SMRT) sequencing technology from Pacific Biosciences (PacBio). In total, more than 187 Gb and 178 Gb of sequence raw data, equivalent to ~288x and ~248x genome coverage of 'Pawnee' and ZAFU-1, respectively, were used to assemble the genomes (Additional file 1: Table S3). The assemblies contain 3,860 ('Pawnee') and 5,449 (ZAFU-1) scaffolds (≥2 Kb), with scaffold N50 of 1.08 Mb ('Pawnee') or 1.22 Mb (ZAFU-1), with 90% of the assembled genomes contained in 682 ('Pawnee') or 732 (ZAFU-1) scaffolds (Table 1; Additional file 1: Table S4). The total assembly size of 651.31 Mb for pecan and 706.43 Mb for Chinese hickory are close to the size estimated by of K-mer statistics (Table 1; Additional file 2: Fig. S1; Additional file 1: Table S5) and flow cytometry (Additional file 2: Fig. S2). The assembled sequences cover over 97% of the genome size. The assembled sizes are slightly larger than the estimated size for 'Pawnee', probably due to the relatively high heterozygosity.

Examination of the GC content distribution indicated that our data were sequenced randomly (Additional file 2: Fig. S3). Read coverage statistics showed that more than 96.8% of Illumina short-insert reads can be aligned back to the final assemblies for both species (Additional file 1:

Table S6). Assessment of gene coverage by CEGMA [34] and BUSCO V [35] revealed that greater than 94% of single-copy genes were assembled completely (Additional file 1: Table S7-S8), which is suggestive of complete assemblies and annotation. These metrics indicate that our assemblies are of high quality and have low error rates.

Genome annotation

Comprehensive repeat sequences of the ‘Pawnee’ and ZAFU-1 genomes revealed over 50% repetitive sequences (50.43% for pecan and 53.67% for Chinese hickory), of which ~85% are transposon elements (TEs) (Table 1; Additional file 1: Table S9). Long terminal repeats (LTRs) comprise the majority of the TEs in both genomes, of which *Gypsy*-like and *Copia*-like elements comprise 15.06% and 15.41% in ‘Pawnee’, and 15.91% and 18.54% in ZAFU-1, respectively (Additional file 1: Table S10). In comparison, the total TE proportions in pecan and Chinese hickory were significantly higher than in the walnut genome (8.4% *Gypsy*-like and 6.57% *Copia*-like) [36]. However, the ratio of *Gypsy*-like LTRs to *Copia*-like LTRs is 0.98 to 1 in pecan and 0.86 to 1 in Chinese hickory, much lower than in walnut and grass species [36, 37].

Predicted protein-coding genes in the ‘Pawnee’ and ZAFU-1 genomes (Table 1) were annotated using a combination of *ab initio* prediction, homology search, and *de novo* assembled transcripts gathered from RNA sequencing of multiple tissues. The hybrid gene-prediction protocol delivered 31,075 gene models in the pecan genome and 32,907 in Chinese hickory genome (Table 1; Additional file 1: Table S11-S12). Statistics on gene structure features showed that the average transcript lengths of annotated genes (not including UTRs) were 4,223 bp (‘Pawnee’) and 4,313 bp (ZAFU-1), significantly longer than in other reference genomes, except apple and grape (Additional

file 1: Table S13). The average number of exons per gene and the average CDS length were close to those of the selected species. Predicted genes were functionally annotated by a consensus approach, revealing up to 95.7% ('Pawnee') and 94.7% (ZAFU-1) of the genes having homologs with known functions in four different public databases (Additional file 1: Table S14).

We also identified similar copies of micro RNAs (miRNAs) or transfer RNAs (tRNAs) between 'Pawnee' and ZAFU-1 genomes (Table 1, Additional file 1: Table S15). Interestingly, the ZAFU-1 genome encodes a larger number of small nuclear RNAs (snRNAs) than does the 'Pawnee' genome. Small nuclear RNAs (snRNAs) primarily function for chemical modifications of other RNAs, and snRNA U3 and U6 in the CD-box subclass were associated with methylation [39]. In the ZAFU-1 genome, about 81% of the snRNAs belongs to CD-box subgroup and may contribute to the function of environmental stress tolerance.

Evolution of *Carya* genus and the two nut-tree genomes

Phylogenetic reconstruction. Phylogenetic reconstruction of 14 genome-sequenced species from Fabales, Fagales and Rosales in Rosids as well as grape revealed a common ancestor of pecan, Chinese hickory and *J. regia* about 20 – 29 million years ago (MYA) (Fig. 1a). The split between pecan and Chinese hickory is estimated to be 10.4 – 20.2 MYA. Re-sequencing data from 16 *Carya* species and two *Juglans* species were mapped to Chinese hickory genome sequences. Of the 16 *Carya* species (Additional file 1: Table S2), the mapping rate ranged from 72.32% to 96.95%, but the two *Juglans* species were only 34.90% and 40.58% (Additional file 1: Table S16), indicating a recent divergence time for *Carya* species. Further, an interspecific phylogenetic topology of 16 *Carya* species was built with *J. regia* and *J. sigillata* as out-group (Fig. 1b). The phylogenetic tree revealed

two major clades, in congruence with the intercontinental disjunctive distribution of our previous reports [3]. Integrating these results with our previous reports [3], we generated the most comprehensive geographical distribution map, to date, with all 20 putative hickory species (not all presented in this research) and the fossil record sites (Fig. 1c). The phylogenetic relationship of the examined species between and within morphological sections is well correlated with the geographic distribution, with clear distinction between EA and ENA *Carya*. Further inclusion of *C. sinensis* (section Rhamphocarya) would be valuable and might clarify its taxonomic position. If *C. poilanei* still exists, it would be very valuable to find, conserve, and evaluate it to determine whether it should be included in Sinocarya or Apocarya [38]. Clear genomic distinction between the ENA sections is arguable.

Genome evolution. Based on the accumulated transversion rate at fourfold degenerate synonymous sites of the third codon position values (4DTv) of the duplicate gene pairs, two whole genome duplication (WGD) events (at ~0.15 and ~0.51) were identified in the orthologous segments within the genomes of pecan, Chinese hickory and walnut (Fig. 1d). This suggests that the three species shared a common ancestor that experienced both a recent duplication event (~66 MYA) and an ancient gamma-triplication event (122 – 164 MYA) in an angiosperm ancestor. The speciation events occurred ~23.9 MYA (between walnut and pecan or Chinese hickory) and ~17.0 MYA (between the two *Carya* species), being consistent with the estimated divergence time of phylogenetic reconstruction. Syntenic analysis revealed that 343 and 342 syntenic gene blocks (five or more genes per block) were found, which were involved in 10,530 and 7,682 paralogous gene pairs in pecan and Chinese hickory genomes, respectively (Additional file 1: Table S17). A high proportion of paralogous gene pairs reside in these collinear blocks, providing strong support for the

co-occurrence of WGD events.

Although LTR TEs contribute to most of the repetitive sequences in pecan and Chinese hickory genomes (Additional file 1: Table S10), the relationship of LTRs and genome expansion is still unknown. The insertion time of all LTRs was dated by divergence analysis for further understanding of genome expansion events in both species. As a result, the LTRs burst time (around 8 MYA) matches speciation time of Chinese hickory (Fig. 1d; Additional file 2: Fig. S4). However, the number of LTRs reached the maximum 2-3 MYA and subsequently fell in pecan. This reflects that the proliferation of LTRs contributed more to the expansion of the Chinese hickory genome than to pecan after the divergent event between them but did not directly contribute to the two major WGD events.

Comparative analyses showed that the four Fagales species – pecan, Chinese hickory, walnut [36] and silver birch [40], share 9,977 gene families with other genome-sequenced Rosids species and 13,953 were common to Fagales species (Fig. 1f). In total, 371 gene families were specific to pecan and 580 to Chinese hickory, respectively. Gene ontology (GO) terms and KEGG pathway enrichment of the genes in the unique gene families highlighted the functions on organic biosynthetic processes and signal transduction (GO), and linoleic acid metabolism (KEGG) in Chinese hickory genome (Additional file 2: Fig. S5; Additional file 1: Table S18). In the pecan genome, the significantly enriched GO term was the reactive oxygen species metabolic process, with no other significantly enriched pathway (Additional file 2: Fig. S5; Additional file 1: Table S18). Gene family expansion-contraction analyses among the 13 Rosids species and grape revealed only 16 expanded gene families in pecan and 59 in Chinese hickory (Additional file 2: Fig. S6). GO terms and KEGG pathway enrichment analyses of genes in the expanded gene families in pecan and Chinese hickory

were also performed (Additional file 2: Fig. S7; Additional file 1: Table S19). Significantly enriched genes were involved in wide GO terms in both species, some of the genes function on pathways related to ROS cleavage (peroxisome and glutathione metabolism), plant-pathogen interactions in pecan, and biosynthesis of secondary metabolites and flavonoid biosynthesis in Chinese hickory.

The genomes and stress adaptation. To explain the molecular basis of abiotic stress adaptation in both species, we identified the genes related to abscisic acid (ABA) metabolism and signaling pathways in both genomes. Only the genes encoding ABA1 and late embryo abundant (LEA) proteins, and R genes had significantly expanded copies (Fig. 2a). Detailed phylogenetic analysis of the core components of ABA signaling (Additional file 2: Fig. S8) demonstrated remarkable duplication on clades of PYL7 – PYL9-like and PYL4-like ABA receptor genes (Fig. 2b) and subclass II and subclass III SnRK2 genes (Fig. 2c). These might account for the enhanced abiotic or biotic stress resistance observed in both species. As marker genes in response to biotic stress, a large number of R genes were identified in both genomes (Fig. 2a) and there were more R genes in Chinese Hickory than in pecan. More R genes might be a reflection of the higher disease resistance and the adaptation of subtropical climate of Chinese hickory. A maximum likelihood tree of LEA protein-encoding genes revealed an extreme expansion in Group 2 LEAs (Fig. 2d), probably the genetic basis of enhanced cellular structure protection under stresses and the high content of storage protein in the nuts of both species.

Identifying differentially expressed genes

Kernels of pecan and Chinese hickory are nutritious and economically valuable. To reveal the mechanisms of the nutritional accumulation, we conducted transcriptomic analysis during embryo development and identified differentially expressed genes (DEGs) in pecan and Chinese hickory. In

comparison, expression levels of DEGs vary significantly among three representative stages of embryonic development: i.e., the early stage (PEY1 and HEY1), the stage with fully extended cotyledons (PEY2 and HEY2) and the fully matured stage of the embryos (PEY3 and HEY3) in pecan and Chinese hickory. In total, we identified 1,043 DEGs (395 up- and 648 down-regulated) in PEY2 *vs* PEY1, and 2,524 DEGs (1,215 up- and 1,309 down-regulated) in PEY3 *vs* PEY2 in pecan. In Chinese hickory, 1,570 DEGs (407 up- and 1,163 down-regulated) in HEY2 *vs* HEY1, and 554 DEGs (261 up- and 293 down-regulated) of HEY3 *vs* HEY2 were identified (Additional file 3). We performed GO enrichment using the significantly up- and down-regulated DEGs based on the hierarchical clustering of DEGs. In pecan, the up-regulated DEGs from the developmental stage PEY1 to PEY2 centered in carboxylic acid biosynthetic process, fatty acid metabolic process, lipid particle, acetyl-CoA carboxylase activity and ligase activity; and the down-regulated DEGs were related to nucleosome assembly, chromatin assembly or disassembly, non-membrane-bounded organelle (Additional file 2: Fig. S9). From PEY3 to PEY2, the up-regulated DEGs centered in peroxisome, embryo development, etc.; and the down-regulated DEGs were related to fatty acid metabolic process, glycolysis, cytoskeletal part, etc. (Additional file 2: Fig. S10). Comparing HEY2 *vs* HEY1, the up-regulated DEGs centered in superoxide metabolic process, lipid particle, nutrient reservoir activity, etc.; and the down-regulated DEGs were related to DNA packaging, microtubule-based process, non-membrane-bounded organelle, etc. (Additional file 2: Fig. S11). Comparing HEY3 vs HEY2, the up-regulated DEGs centered in oxidation-reduction process; and the down-regulated DEGs were related to glycolysis, microtubule, potassium ion binding, etc. (Additional file 2: Fig. S12).

Pecan and Chinese hickory oil-abundant tree nuts

One of the key healthy traits of pecan and Chinese hickory nuts is an abundance in oil (over 70% of fresh weight) (Fig. 3) [41]. To reveal the underlying genetic mechanism, we identified all genes involved in fatty acid metabolism in pecan and Chinese hickory genomes by using *Arabidopsis* homolog protein sequences as query (Additional file 1: Table S20). Compared to other diploid oil plants [42, 43], the Chinese hickory genome harbored more genes involved in oil accumulation, for both fatty acid *de novo* synthesis and triacyl-glyceride (TAG) assembly pathways, but less than that of soybean due to an additional WGD event about 13 MYA [44]. The pecan genome ranked third in the total number of genes related to oil synthesis (Additional file 1: Table S20). Most of the oil synthesis-related gene homologs in pecan and Chinese hickory are abundant in transcripts during embryo development, suggesting an important role in the synthesis of unsaturated fatty acids.

Further analyses revealed significant expansion of gene families encoding key enzymes and important transcription factors in pecan and Chinese hickory and other selected oil plants (Fig. 3b; Additional file 1: Table S20). One of the expanded gene families encodes acetyl-CoA carboxylases (ACCases), which converts acetyl-CoA to malonyl-CoA as a rate-limiting enzyme in fatty acid *de novo* synthesis [45]. The plastidic heteromeric ACCase includes four subunits, i.e. α-CT, β-CT, BC, and BCCP. Pecan and Chinese hickory genomes harbor more copies (9 and 10, respectively) than most other oil plants but are similar to soybean (Additional file 1: Table S20). Transcriptomic analysis showed significant transcript accumulation of the homologs of *ACCase* and *DGAT* at oil accumulation stages during embryo development in both species. The expanded gene copies and their high expression levels likely impact high oil level in the nuts of pecan and Chinese hickory (Fig. 3b; Supplementary Tables S21-S22) [46]. Δ-9-stearoyl-ACP desaturase (SAD) is a crucial enzyme

for *de novo* synthesis of unsaturated fatty acids in oil plants [47], and the transcript abundance of encoding genes was enriched only in the Chinese hickory genome (Fig. 3b; Additional file 1: Table S22). Phylogenetic analysis of the family revealed a unique clade to species in Juglandaceae (Fig. 3c). Furthermore, these homologs were abundant in transcripts during the embryo development, indicating an important role in the synthesis of unsaturated fatty acids (Fig. 3b; Additional file 1: Tables S21-S22).

Pecan and Chinese hickory have more copies of two kinds of key transcription factors, *WRIs* and *PIIs*, than other diploid oil plants, except for soybean (Additional file 1: Table S20). Of them, *WRI1* and *PII* are significant accumulated transcripts during embryo development in both species (Fig. 3b). Expression of fatty acid desaturase (FAD) family members regulates different fatty acids components and ratios [48]. FAD3 catalyzes the critical step of converting linoleic acid (18:2) to linolenic acid (18:3) while FAD5 plays a major role in the transformation of palmitic acid (C16:0) to palmitoleic acid (C16:1) [49]. In contrast to other oil plants, no FAD3 or FAD5 homolog is encoded by the oil palm genome, whose seeds are rich in saturated fatty acids. This suggests that the high levels of unsaturated fatty acid in Chinese hickory, pecan and other oil plants are probably due to the additional FAD members. The expansion and high expression levels of genes related to unsaturated fatty acids biosynthesis provides genomic evidence and genetic basis for the high proportion of unsaturated fatty acid level in *Carya* nuts.

Pecan and Chinese hickory as polyphenol-, arginine- and B vitamins-rich tree nuts

Polyphenols, as secondary metabolites and potential antioxidative compounds, are involved in multiple aspects of plant development and defense [50], and have additional value for human health

[47, 52]. Phenolic compounds are primarily derived from flavonoid biosynthesis, which includes the pathways of anthocyanin, the proanthocyanidin (PA), and flavonol pathways [51] (Fig. 4a). We identified the genes that are related to anthocyanins biosynthesis and regulation among 11 selected species including *Arabidopsis*, pecan and Chinese hickory (Additional file 1: Table S23). We found that most of the gene families had no significant expansion among the selected species beyond that seen in *Arabidopsis*. Chalcone synthase (CHS), the first enzyme triggering the pathway, has one or two additional copies in pecan, Chinese hickory and walnut (Additional file 1: Table S23). Leucoanthocyanidin reductase (LAR), a key enzyme in PA biosynthesis, also showed significant expansion and was not encoded by *Arabidopsis* and tomato genomes (Additional file 1: Table S23). Both *CHS* and *LAR* gene families, together with WRKY transcription factors exhibited a Juglandaceae-specific expansion (Additional file 2: Fig. S13-S14; Additional file 1: Table S23). Expression profile analysis showed that the majority of genes involved in the PA biosynthesis pathway had a relatively high expression level during embryo development in both pecan and Chinese hickory (Fig. 4b). These results provide genomic support for pathways leading to the high polyphenol content in the nuts.

Pecan and Chinese hickory nuts are valued not only for their high oil content but also for their high protein content and richness in essential amino acids [10]. We examined the key genes involved in biosynthesis of 10 amino acids, including 8 essential, 1 semi-essential (arginine) and 1 essential only for children (histidine). Of them, arginine is the most abundant and there are nine enzymes involved in its biosynthesis. All of the encoding genes of the arginine enzymes have significantly expanded copy number compared to *Arabidopsis* and have medium copies among diploid oil plants (Fig. 4d and 4e; Additional file 1: Table S24). Most genes encoding enzymes involved in other 9

amino acid biosynthesis have similar trends in copy numbers in both pecan and Chinese hickory genomes (Additional file 1: Table S24).

Pecan and Chinese hickory nuts also contain high levels of vitamin B, especially thiamine (vitamin B1) [10]. Thus, we examined the key enzymes involved in vitamin B biosynthesis of the *Carya*, and walnut genomes, and all other sequenced oil associated plant genomes and *Arabidopsis* as well as rice (Fig. 4d and 4e; Additional file 1: Table S25). We found that gene copies encoding one of the enzymes involved in vitamin B1 biosynthesis (EC 2.5.1.3), one enzyme catalyzing vitamin B6 biosynthesis (EC 1.1.1.65) and two enzymes generating vitamin B2 (EC 2.7.7.2 and EC 3.1.3.104) are significantly higher than those in *Arabidopsis* but in the middle of diploid oil plants (Fig. 4d and 4e; Additional file 1: Table S25).

Discussion

Evolutionary history of *Carya* species

The genus *Carya* exhibits a remarkable disjunctive distribution between EA and ENA, which offers a model for understanding the phylogenetic relationship between EA and ENA species. By combining the analysis of 8 plastid and 2 nuclear loci in 16 *Carya* species with fossil and morphological data, we investigated the phylogenetic relationships between EA and ENA species and reconstructed the historical biogeography of *Carya* [3]. The results clarified the boreotropical flora hypothesis and North Atlantic land bridge (NALB) as a crucial route for the spread of *Carya* species from North America to Europe to EA. Although the results from Zhang et al. [3] strongly supported the intercontinental disjunctions in *Carya*, use of only ten loci is still not sufficient for fully exploring the phylogenetic pattern of intra-continental species in EA and ENA.

By comparison, our high-quality genome sequences of pecan and Chinese hickory together with the re-sequencing data from 14 other *Carya* species, offers a much greater number of molecular loci genome-wide. These attributes are important for enhancing phylogenetic accuracy and the reliability of phylogenetic relationships among *Carya* species. The phylogenetic tree (Fig. 1b) offers strong support for the intercontinental disjunctions in *Carya* and the inferences regarding origin and distribution during *Carya* evolution, as suggested by Zhang et al. [3]. The phylogenetic relationship among species within morphological sections is well correlated with the geographic distribution, at least for the Asian hickories evaluated. Distinction between ENA sections Apocarya and Carya is clear based on morphology, but is not evident by a clear phylogenetic division of the species evaluated here.

Integrating these analyses with the previous and recent studies as well as the fossil record [3], we generated the most comprehensive geographical distribution map containing 20-extant hickory species and the fossil record sites (Fig. 1c). As our previous discussion [3], the extant *Carya* species formed two distribution centers in EA and ENA. All of the results allow us to speculate that the present disjunctive distribution of *Carya* species between EA and ENA might be the result of extinctions in large parts of its former ranges. *Carya* was more broadly distributed across North America and dispersed to West Europe by North Atlantic Bridge in the Miocene, and continually spread to central Europe and Asia in Miocene, and to Japan in Neogene (Fig. 1c). The present distribution of *Carya* species might be the result of extinctions in large parts of its former ranges. Subsequently, climatic cooling resulted in the original extinction events that caused the range fragmentation in *Carya* and ultimately lead to speciation. As the representatives of EA and ENA *Carya* species, pecan and Chinese hickory depict independent evolution of discontinuously

distributed species originating in East Asia and North America. After diverging from their common ancestor, they have been evolving independently, representing clades that share distinctive biological and physiological characters and ecological adaptation.

Adaptive evolution of pecan and Chinese hickory

The Asian *Carya* species, including Chinese hickory, have restricted geographical distributions with specific ecological requirements [52]. Chinese hickory is restricted to a narrow area of subtropical climate in East China [53] but has resistance to scab [54]. In contrast, *Carya* species in North America, including the native pecan, are adapted to a wide range of climate types from mild to harsh condition and exhibit high resistance to multiple abiotic stresses [55]. This wide adaptability has resulted in worldwide commercial cultivation and numerous cultivars and hybrid lines of pecan [26, 56]. Pecan is adapted across climatic regions with a wide variety of precipitation, temperature and soils. It is a riparian species that thrives on soil moisture, but occurs on alkaline calcareous soils of west Texas as well as more neutral soils of the Mississippi River and its tributaries in the east. Chinese hickory requires the moist conditions of rainy subtropical or tropical areas. Morphologically, the buds of North America *Carya* species are covered by bud scales [21], which provide protection for young apical meristems and contributes to the adaptation to wider latitudes. In contrast, the naked buds of Asian *Carya* species have restricted their distribution to only in subtropical and tropical areas. Comparative analysis shows the expanded gene families in pecan are significantly enriched in functions associated with response to oxidative stress, biotic defense response, stimulus, wounding, metal ion exposure, etc. However, the expanded gene families in Chinese hickory are mainly associated with plant-pathogen interaction. This analysis suggests the genetic basis for adaptation to

climates and stress resistance between pecan and Chinese hickory. Presence or absence of bud scales are results, but not causes.

The phytohormone ABA, protective LEA proteins, antioxidative enzymes (such as SODs, PODs and PLDs), detoxifiers (such as GSTs), and R genes, are often considered to be key components of response to abiotic and/or biotic stress [50, 57]. Similar copies of the key genes for enzymes in ABA metabolism and signaling were identified in pecan and Chinese hickory genomes. However, the detailed analysis of core components of ABA signaling, PYL receptors and SnRK2 kinases, revealed a large expansion of subclasses of the PYL7-PLY9 clade, the PYL4 clade and SnRK2 subclass II – III, compared with *Arabidopsis*. The extreme expansion of the R genes and LEA proteins, specifically expanded group 2 LEAs in both genomes enhanced the protective roles under stress condition. These probably reflect increased resistance to abiotic and biotic stresses in woody plants. Meanwhile, the increase in copies of R genes in pecan more than in Chinese hickory provides the genetic basis for the ability to cope with biotic stress in this species.

Moreover, biogeographic studies suggest that the extent of fatty acid unsaturation in oil seeds played an important role in temperature adaptation on both a micro- and macro-evolutionary scale [58, 59]. It is worth mentioning that stearoyl-acyl carrier protein D9/desaturase 6 (SAD9/DES6), a fatty acid desaturase, plays vital roles in drought and hypoxia stress in *Arabidopsis* [60]. Here, the existence of two DES6 genes in pecan genome versus a single copy in Chinese hickory is consistent with the stronger resistance to drought and hypoxia found in pecan.

Genetic basis of nut nutritional value

As delicious and nutritional foods, pecan and Chinese hickory nuts are valued for not only their

high-unsaturated fatty acids and anti-oxidative polyphenols, but also richness in proteins, fiber, minerals and vitamins. LEA proteins, as the major seed storage proteins, not only play protective roles in the responses to stresses, but also provide resources of nutritional value. The significant expansion of LEA encoding genes in both genomes may provide an explanation of the observed high protein content in the nuts of pecan and Chinese hickory. Although our transcriptomic analyses of biosynthesis pathways of oil and proanthocyanidins during development of embryos of pecan and Chinese hickory provided valuable clues [46, 61], the molecular mechanisms are still unknown. The specific expansion on genes encoding several key enzymes in oil biosynthesis, combing with the expression profiles, provided fundamental basis to further investigate the underlying mechanism.

The nutritional value and health benefits of pecan and Chinese hickory offer potential for enhanced food security. The high-quality reference genome assemblies presented here will accelerate improvements of pecan and Chinese hickory. Major breeding objectives for their improvement include the development of shorter plants with more branches and more and larger fruits, increased water and biotic stress resistance, and the introgression of the sweet phenotype into commercial varieties. The expansion of certain gene families on stress resistance, oil accumulation and polyphenol biosynthesis, provide foundational basis and will also help direct future breeding strategies. The genome sequences presented here also make *Carya* species useful models for studying the EA-ENA disjunctive distribution, and mechanisms of adaptive evolution and nutritional component accumulation in nut plants.

Materials and Methods

1 Genome sequencing and assembly

1.1 Plant materials

Pecan and Chinese hickory represent the only two commercially cultivated nut species in *Carya* (Juglandaceae). To generate high-quality reference genomes, a pecan cultivar – *C. illinoinensis cv.* 'Pawnee' from a controlled cross 'Mohawk' X 'Starking Hardy Giant' which is widely distributed across Asia and North America [29], and a Chinese hickory landrace (ZAFU-1) from the Tianmu Mountains of the Lin'an area of Hangzhou city, in Zhejiang province, China were selected for whole genome sequencing (WGS). Leaves of pecan and Chinese hickory, 14 other *Carya* species (10 species from the US and 4 from Asia) and two *Juglans* species were also selected for whole genome re-sequencing. Young expanding leaves from all species were harvested and stored at -80°C prior to DNA extraction. To aid protein-coding gene annotation, young leaves, epicarps, embryos and vegetative shoots were collected from both pecan and Chinese hickory, and pistillate and staminate buds, staminate inflorescences were only from ZAFU-1. RNAs were isolated using Trizol Reagent (Cat. 15596-026, Invitrogen, USA).

1.2 DNA extraction and whole genome sequencing

High molecular weight genomic DNA from 'Pawnee' and ZAFU-1 was extracted using the CTAB method [62]. Genome sequencing was performed on Illumina Hiseq X-ten and PacBio RS II platforms for both species. For Illumina HiSeq X-ten platform, the genomic DNA was sheared with a Bioruptor sonication device (Diagenode SA, Liege, Belgium) and a Hydroshear DNA Shearing Device (Genomic Solutions Inc., Ann Arbor, MI, USA) for short insert paired-end (PE) and large-insert mate-pair (MP) library construction, respectively. DNA libraries of PE (250 and 500 bp) and MP (2, 5, 10 and 20 kb) were prepared and then sequenced for both species, respectively, according to the manufacturer's instructions (Illumina, San Diego, CA). SMRTbell libraries with an

insert size of 20 kb were constructed after twice DNA purification with Beckman Coulter Genomics AMPure XP magnetic beads. The genomes were sequenced on PacBio RS II platform (Pacific Biosciences, USA) using the P6 polymerase/C4 chemistry combination, based on the manufacturer's procedure. The reads of mitochondria or chloroplast were filtered out before we assembled the genome sequences of the two *Carya* species using two steps: (1) Illumina reads were mapped to *Juglans regia* chloroplast reference genome (GenBank: MF167464.1) using BWA (v0.5.9-r16) with default parameters; (2) PacBio reads were mapped to the *J. regia* chloroplast reference genome using BLASR with default parameters [63].

1.3 Genome size estimation

The genome size of pecan and Chinese hickory was estimated using two methods: flow cytometry and K-mer analysis. The DNA content of DAPI stained nuclei from 'Pawnee' and ZAFU-1 was measured on a flow cytometer (Cyflow Ploidy Analyser, Partec), using *Prunus mume* as internal standard. Genome size was calculated based on the formula: mean DNA content at G1 peak of pecan or Chinese hickory / mean DNA content at G1 peak of *P. mume*) × *P. mume* genome size (280Mb). The 17-mer frequencies were generated using 57.80 Gb (pecan) and 47.91 Gb (Chinese hickory) high-quality PE reads (250 bp) and the genome size was estimated as described by Li et al. [64].

1.4 Genome assembly

1.4.1 Illumina raw data processing

PCR duplicates were removed using in house scripts that have been posted to Github[65]. Quality control involved the following steps using our scripts: (1) The PE reads were discarded when either read contains adapter sequence, more than 10% uncertain nucleotides (N), or more than 20% low quality bases (Phred quality < 5). (2) MP reads that did not hit the linker were used only in support of

links found with the filtered MPs, but were not used to create links independently. (3) For the TrueSeq MP data, reads were filtered out for those with low quality bases (>50% bases with Q-value ≤ 8), with Ns > 10% of the read length and with adaptor sequence. Then a total of 135.29 Gb and 123.31 Gb (208.21-fold and 170.93-fold coverage of the estimated genomes) Illumina HigeSeq X-ten clean data were used for the assembly of pecan and Chinese hickory, respectively (Additional file 1: Table S3).

1.4.2 *De novo* genome assembly using Illumina Hiseq X-ten data

Due to the high heterozygosity of the genomes (~ 1.46% of pecan and ~ 0.77% of Chinese hickory), we assembled the filtered clean data using Platanus (PLATform for Assembling NUcleotide Sequences) (Platanus, RRID:SCR_015531) [66], a novel *de novo* sequence assembler that can reconstruct genomic sequences of highly heterozygous diploids from massively parallel shotgun sequencing data. We obtained the initial assemblies (V1.0) for both species with the following parameters: “contig (-u 0.2 -a 15 -c 26), scaffold (-u 0.2)” for pecan, and “contig (-u 0.2 -a 15 -c 20), scaffold (-u 0.2)” for Chinese hickory.

1.4.3 Improving the *de novo* assemblies using PacBio data

To get the final assemblies, PBJelly v12.19.14 (PBJelly, RRID:SCR_012091) [67] and GapCloser v1.12(GapCloser, RRID:SCR_015026) [68] were used to fill gaps in V1.0 assemblies using PacBio RSII data, approximately 40X (pecan) and 30X (Chinese hickory) of the estimated genomes, respectively. In brief, PBJelly began with a “Setup” process that automatically identified gaps. Any stretch of 25 or more N’s within a scaffold defines a gap. SMRT reads were aligned to V1.0 assemblies using BLASR (Basic Local Alignment and Serial Refinement) [63] (V5.0), which was specifically designed with the PacBio data error model in mind. The BLASR alignment information

is parsed to identify gap-supporting reads. After the gap-supporting sequencing reads were identified, PBJelly assembled the reads for each gap to generate a high-quality gap-filling consensus sequence.

1.5 Quality evaluation of the final genome assemblies

We used two different data sets to evaluate the quality of the final assemblies of both species. First, the high-quality Illumina reads that were generated from short insert size PE libraries were mapped to the scaffolds using BWA mem [69]. To assess completeness of the genome assembly, the distribution of the sequencing depth at each position was calculated using SAMtools v1.6 (SAMTOOLS , RRID:SCR_002105) [70]. The GC content distribution was examined to analyze nucleotide distribution and assess the randomness of sequencing.

In order to evaluate the quality of the genome assemblies, RNAs from young leaf tissues of both species were respectively sequenced using 250 bp libraries with PE150 on Illumina HiSeq X-ten platform. A total of 3.17 Gb and 2.99 Gb of transcriptomic data were assembled using Trinity v2.1.1 (Trinity , RRID:SCR_013048) [71], and generated 73,093 and 39,583 unigenes for pecan and Chinese hickory, respectively. These unigenes were then mapped to the scaffolds using BLAT (BLAT, RRID:SCR_011919) [72].

Additionally, CEGMA (CEGMA, RRID:SCR_015055) [34] (Core Eukaryotic Genes Mapping Approach) pipeline and BUSCO v3(BUSCO , RRID:SCR_015008) [35] (Benchmarking Universal Single-Copy Orthologs) were also used to assess the completeness of the genome assemblies or annotations.

2 Transcriptome sequencing

To aid the protein-coding gene annotation in the both species, 4 sequencing libraries from four

tissues (young leaves, epicarps, embryos, and vegetative shoots) of pecan, and 7 tissues (young leaves, pistillate and staminate buds, staminate inflorescences, vegetative shoots, pericarps and embryos) of Chinese hickory were constructed using VAHTS standard mRNA-Seq Prep Kit (Vazyme Biotech Co., Ltd) for Illumina. A total of 30.94 Gb raw data was generated for pecan and 52.06 Gb for Chinese hickory.

3 Genome resequencing and data analysis of 16 *Carya* species and 2 *Juglans* species

Pecan and Chinese hickory, 14 other *Carya* species (10 species from the US and 4 from Asia) and two *Juglans* species were also selected for whole genome re-sequencing. Young expanding leaves from all species were harvested and stored at -80°C prior to DNA extraction. DNA from single plants was extracted using the CTAB method [63]. The 125-bp PE libraries were sequenced using Illumina NextSeq 500 technology. The data was processed for base calling, quality evaluation, removing the adaptor sequence, and filtering low-quality sequences using CASAVA v1.82(CASAVA, RRID:SCR_001802) [73] and FastQC software (FastQC , RRID:SCR_014583) [74]. The remaining clean reads were mapped to Chinese hickory reference genome using BWA (BWA, RRID:SCR_010910) [66] (v0.5.9-r16) with the command 'mem -t 4 -k 32 -M'. In order to reduce mismatch generated by PCR amplification before sequencing, duplicated reads were removed by the aid of SAMtools [70]. After alignment, we performed SNP calling on a population scale using a Bayesian approach as implemented in the package SAMtools. We then calculated genotype likelihoods from reads of each individual at each genomic location, and the allele frequencies in the sample with a Bayesian approach. To exclude SNP calling errors caused by incorrect mapping, only high quality SNPs (coverage depth ⩾3, RMS mapping quality ⩾20, maf ⩾0.05, miss⩽0.1) were

kept for subsequent analysis.

4 Genome annotation

4.1 Repetitive sequences annotation

We predicted TEs in the pecan and Chinese hickory genomes by combining the *de novo*-based and the homology-based approaches. The *de novo* repeat libraries were built by using RepeatModeler v1.0.9(RepeatModeler, RRID:SCR_015027) [75], a *de novo* repeats family identification and modeling package, for both species, separately. For the homology-based approach, we used RepeatMasker v3.3.0(RepeatMasker , RRID:SCR_012954) [76] against the Repbase TE library, and RepeatProteinMask against the TE protein database, respectively. Tandem repeats were detected in the genomes using the software Tandem Repeats Finder (TRF) [77].

4.2 Identification of protein coding genes

To predict protein-coding genes in the pecan and Chinese hickory genomes, we integrated three approaches – homolog-based, *de novo* and transcriptomic aiding predictions. Homolog proteins from ten plant genomes (*Cucumis sativus, Citrullus lanatus, Prunus persica, Malus domestica, Vitis vinifera, Glycine max, Eucalyptus grandis, Arabidopsis thaliana, Populus trichocarpa and Oryza sativa*) were downloaded from Ensemble [78] and JGI [79]. Protein sequences from these genomes were aligned to the pecan and Chinese hickory genome assembly using TblastN, respectively, with an E-value cutoff of 1e-5. The BLAST hits were conjoined by Solar software [80]. GeneWise (GeneWise, RRID:SCR_015054) [81] was used to predict the exact gene structure of the corresponding genomic regions on each BLAST hit (Homo-set). For transcriptome-based prediction methods, RNA-seq reads were mapped to the assembly using TopHat v2.0.8 (TopHat ,

RRID:SCR_013035) [82], and Cufflinks v2.1.1 (Cufflinks, RRID:SCR_014597) [83] and then used to assemble the transcripts into gene models (Cufflinks-set). In addition, RNA-seq reads were assembled by Trinity v2.1.1 [71] and were also mapped to the assembly and gene models were predicted by PASA [84]. This gene set was denoted as PASA-T-set (PASA Trinity set) and was used to train *ab initio* gene prediction programs. Five *ab initio* gene prediction programs, AUGUSTUS v2.5.5 (Augustus , RRID:SCR_008417) [85], GenScan v1.0 [86], GlimmerHMM v3.0.1 (GlimmerHMM , RRID:SCR_002654) [87], Geneid (V1.3) [88], and SNAP [89], were used to predict coding regions in the repeat-masked genome. Gene model evidence from Homo-set, Cufflinks-set, PASA-T-set and *ab initio* programs were combined by EvidenceModeler (EVM) [90] into a non-redundant set of gene structures.

4.3 Functional annotation protein-coding genes

Functional annotation of protein-coding genes was achieved using BLASTP (E-value 1e-05) [91] against two integrated protein sequence databases: SwissProt and NCBI-nr. Protein domains were annotated by searching against the InterPro v32.0 (InterPro , RRID:SCR_006695) [92] and Pfam (V27.0) [93] databases, using InterProScan v4.8 (InterProScan , RRID:SCR_005829) [94] and HMMER

(V3.1) [95], respectively. The GO terms for each gene were obtained from the corresponding InterPro or Pfam entry. The pathways in which the genes might be involved were assigned by BLAST against the KEGG databases (release 53), with an E-value cutoff of 1e-05.

4.4 Annotating non-coding RNAs

Noncoding RNA genes, including rRNAs, tRNAs and snRNAs were predicted in the assemblies. The tRNA genes were identified by tRNAscan-SE [96] software with the eukaryote parameters. The

rRNA fragments were predicted by aligning to *Arabidopsis* and rice template rRNA sequences using BLASTn at an E-value of 1e-10. The miRNA and snRNA genes were predicted by searching against the Rfam database (release 9.1) [97] using INFERNAL software (Infernal, RRID:SCR_011809) [98].

5 Evolutionary analyses of the genomes and *Carya*

5.1 Phylogenetic analysis

Except for pecan and Chinese hickory, *Vitis vinifera* and 10 other genome-sequenced representatives from the Rosids (*Juglans regia*, *Glycine max*, *Medicago truncatula*, *Prunus persica*, *Morus notabilis*, *Carica papaya*, *Gossypium hirsutum*, *Theobroma cacao*, *Betula pendula* and *Populus trichocarpa*), along with *Arabidopsis thaliana*, were selected for constructing phylogenetic tree (*Juglans regia* genome data were downloaded from [99]; others were downloaded from Phytozome [100] (v12)). The protein set of each species was obtained and filtered as follows: (1) only the longest isoform was considered for further analysis if a gene encoded several isoforms; (2) proteins less than 30 amino acids were filtered out. The similarity relation between homologous proteins in all species was obtained through BLASTp with the e-value 1e-5. All the protein datasets of 14 species were clustered into paralogous and orthologous using the program OrthoMCL [101] with the inflation parameter 1.5. Finally, 170 single-copy-gene encoded proteins were used for the phylogenetic analysis. The protein sequences from all species were then aligned by MUSCLE (MUSCLE, RRID:SCR_011812) [102] and generated a super alignment matrix by combining all the alignment results. A phylogenetic tree containing 14 species was constructed using RAxML (RAxML, RRID:SCR_006086) [103] with the maximum likelihood method and 1000 bootstraps. Finally, the MCMCtree program implemented in the Phylogenetic Analysis by Maximum Likelihood (PAML)

(PAML , RRID:SCR_014932) [104] was applied to infer the divergence time based on the phylogenetic tree. The MCMCtree running parameters were: burn-in: 5,000,000, sample-number: 1,000,000, sample-frequency: 50. The calibration times of divergence between *A. thaliana* and *C. papaya* (54-90 Mya), *G. hirsutum* and *T. cacao* (32-99 Mya), *A. thaliana* and *P. trichocarpa* (107-109 Mya), *G. max* and *M. truncatula* (46-60 Mya), *M. notabilis* and *P. persica* (73-90 Mya), *A. thaliana* and *G. max* (107-111 Mya) were obtained from the TimeTree database [105].

5.2 Comparative genomes

Chi-square test, as one of the widely used hypothesis test methods, was used to test the expansion and contraction of gene families in both pecan and Chinese hickory. The gene number of gene families was compared among Rosales (*P. persica* and *M. notabilis*), Fabales (*G. max* and *M. truncatula*) and Fagales (English walnut, pecan and Chinese hickory). Pecan, Chinese hickory and English walnut (*J. regia*) were further compared by gene number of gene families. Gene families were considered expanded if the number of genes in one species was significantly ($P<0.05$) more than that in other species by chi-square test.

5.3 Distribution of hickories and phylogenetic reconstruction of *Carya*

The geographical distribution (longitude, latitude, altitude, habitat, etc.) for extant *Carya* species in East Asia was achieved from Chinese Virtual Herbarium [106], which is an online access to herbarium specimens and botanical information chiefly constructed by Institute of Botany, Chinese Academy of Sciences and partially from National Specimen information infrastructure [107], another online sharing platform of teaching samples. Similarly, the information of extant species in North America was obtained from Natural Resources Conservation Service [108]. The distribution of the extinct hickories was retrieved from a literature [3]. Finally, a distribution map of all the extant and

extinct species was generated by the on-screen digitization and visual interpretation techniques using ArcGIS 10.2 software [109].

To estimate the phylogenic relationship between species in *Carya*, the 125-bp paired-end reads in pecan and Chinese hickory, as well as the re-sequencing data of other 14 *Carya* species and two *Juglans* out-group species were re-sequenced using Illumina NextSeq 500. The raw data were processed for base calling, quality evaluation, removing the adaptor sequence, and low-quality sequence using CASAVA (v1.82) and FastQC software, with the following steps: (1) removing reads with ≥ 10% unidentified nucleotides (N); (2) removing reads with > 20% bases having Phred quality < 5; (3) removing reads with >10 nt aligned to the adapter, allowing ≤ 10% mismatches; (4) removing putative PCR duplicates generated by PCR amplification during the library construction process (i.e. read 1 and 2 of two paired-end reads that were completely identical).

The remaining high quality paired-end reads were mapped to the ZAFU-1 genome using BWA (Burrows-Wheeler Aligner) (Version 0.7.8) with the command 'mem -t 4 -k 32 –M'. After alignment, SNP calling on a population scale was performed using a Bayesian approach as implemented in the package SAMtools (Version 1.4). Genotype likelihoods were calculated with a Bayesian approach from reads for each individual at each genomic location and the allele frequencies in the sample. To exclude SNP calling errors caused by incorrect mapping, only high quality SNPs (coverage depth ⩾ 3 , RMS mapping quality ⩾20, maf ⩾0.05, miss⩽0.1) were used for further analysis.

To clarify the phylogenetic relationship from a genome-wide perspective, an individual-based neighbor-joining (NJ) tree was constructed with 1000 bootstraps using the software TreeBestv1.9.2 [110]. The MCMCtree program implemented in the Phylogenetic Analysis by Maximum Likelihood (PAML) was applied to infer the divergence time based on the phylogenetic tree. The MCMCtree

running parameters were: burn-in:model:JC69, burnin:10,000, nsample:100,000,sampfreq:2.

5.4 Whole Genome Duplication

To identity syntenic blocks, the protein sequences from pecan, Chinese hickory and English walnut [36] were searched against themselves using BLASTp (E<1e-5). The results were subjected to MCScan [111] (-a, -e:1e-5, -u:1, -s:5) to determine syntenic blocks. At least 5 genes were required to define a synteny. We calculated the 4DTv distribution for each gene pair from the aligned blocks to estimate the speciation or WGD event that occurred during the evolutionary history of the two hickories. The 4DTv analysis and WGD divergence time were estimated as described by The Potato Genome Sequencing Consortium [112].

5.5 Insertion time estimate of LTRs

LTRharvest [113] and LTRfinder were used to predict LTR-RTs with the parameters: LTR length of 100-5000bp, LTRs interspace length of 1000-20000bp. The tRNAscan-SE was used for predicting tRNA sequences and LTRdigest [114] was used for structure annotation (e.g., PBS, PPT, protein, etc.) of LTR-RTs with optimal annotation. LTR-RTs were clustered by USEARCH software with the similarity parameter of 70%. The LTR-RTs with copy number more than two or single copy containing protein domains were recruited. After that, the nucleotide variations (λ) in 5' and 3' terminals of intact LTR-RTs were estimated by MUSCLE [102]. If λ was greater than 0.75, the intact LTR-RT would be considered invalid. For those valid intact LTR-RTs, the genetic distances (K) were calculated by $K=-0.75\ln(1-4\lambda/3)$. Finally, the insertion time of LTR-RTs was calculated based on the formula: $T=K/2r$ ($r=1.3\times10\text{-}8$ per site and per year), and distributions were further plotted.

6 Identification and phylogenetic analysis of selected genes

The protein sequences related to stress response, oil accumulation and antioxidants in *Arabidopsis*

thaliana were downloaded from NCBI. Then using *Arabidopsis* homologs as query, we identified the candidates in pecan and Chinese hickory by BLASTp with best hit. If these genes were in a common family in OrthoMCL or the E-value < 1e-20, candidate genes were predicted by Pfam [93]. Only candidate genes with the same protein domain were selected. All the amino acid sequences were aligned using ClustalW implemented in the MEGA v7.0 software [115]. The phylogenetic trees were generated with MEGA, using the Maximum likelihood method based on the Jones-Taylor-Thornton (JTT) matrix model, with 1,000 bootstrap replications each. The genes in a phylogenetic tree were further classified to several subfamilies according to intrinsic domains or referring to the phylogenetic tree in *Arabidopsis*. Gene structure was plotted according to its CDS and domain using GSDS software [116].

7 Transcriptome analysis during embryo development

Raw transcriptomic data representing three key stages (i.e., the early and fully extended stages of cotyledon development, and the fully matured stage of the embryos) during embryo development in two pecan trees as biological replicates were deposited in NCBI database (SRR6793957, SRR6793955 and SRR6793961 for replicate 1; SRR6793958, SRR6793956 and SRR6793962 for replicate 2). Raw transcriptomic data representing three key stages during embryo development in Chinese hickory were deposited or downloaded from NCBI database. For each stage, samples that were collected in the same season of years 2012 (SRR6785066, SRR2006624 and SRR2006626) and 2013 (SRR6785065, SRR2006629 and SRR2006631) were treated as two biological replicates in Chinese hickory. To link the genome features and the transcriptomic responses, we analyzed or reanalyzed the data based on our assemblies and annotations. Briefly, high quality filtered reads were mapped to the draft reference genomes with SOAP aligner (Soap2.21) [117] (mismatches > 2 bases).

The expression level (FPKM value) for each protein-coding gene was calculated by Cufflinks [83] using default parameters. Genes with FPKM > 0.5 were defined as expressed. For those genes with more than one transcript, the longest was used to calculate expression level and coverage for each gene. DESeq2 [118] were used for normalizing gene expression (BaseMean) in each sample, and identified DEGs for each compared group by using “P-adj (adjusted p value) < 0.05 and the |log2Ratio|> 1” as the threshold. The DEGs were further grouped into 8 clusters based on their temporal expression patterns.

To obtain the significantly enriched GO term for DEGs, all DEGs were mapped to GO terms in the GO database [119]. GO enrichment analysis of differentially expressed genes was implemented by the GOseq R package [120], in which gene length bias was corrected. GO terms with adjusted P-value less than 0.05 were considered significantly enriched by differentially expressed genes, and labeled with asterisks (*). The significantly enriched GO terms were selected using a hyper-geometric test to develop hierarchical clusters of a sample tree by Euclidean Distance. To further clarify the biological functions of DEGs, a pathway-based analysis was conducted using the public KEGG pathway-related database [121]. We used KOBAS software to test the statistical enrichment of differential expression genes in KEGG Pathways [122]. Pathways with Q-value < 0.05 were considered as significantly enriched. We drew the heatmap of expression levels using pheatmap [123] for the selected genes that we were interested in.

Abbreviations

4DTv: fourfold degenerate synonymous sites of the third codon position values; ABA: abscisic acid; ACCase: acetyl-CoA carboxylase; CHS: Chalcone synthase; CTAB: cetyltrimethylammonium

bromide; DEG: differentially expressed gene; EA: East Asia; ENA: eastern North America; FAD: fatty acid desaturase; GO: Gene Ontology; LEA: late embryo abundant; LAR: Leucoanthocyanidin reductase ; LTR: Long Terminal Repeat; MP: mate-pair; MYA: Million Years Ago; NALB: North Atlantic land bridge; PA: proanthocyanidin; PE: Paired End; SAD: Δ-9-stearoyl-ACP desaturase; TAG: triacyl-glyceride; TE: Transposable Element; TRF: Tandem Repeats Finder; WGD: Whole Genome Duplication; WGS: whole genome sequencing.

Availability of data and materials

The genome sequences, annotation data and resequencing data (Bioproject ID PRJNA435846) are available in GenBank of National Center for Biotechnology Information (NCBI). Assemblies, annotations, and other supporting data are also available in the *GigaScience* database, GigaDB [65].

Acknowlegements

We thank Dr. Yuxian Zhu at Wuhan University, Wuhan, China, for comments on the manuscript. We also thank Dr. Dong Pei at Chinese Academy of Forestry, Beijing, China, for providing DNA samples of *J. regia* and *J. sigillata*. We would like to specially thank one of our co-author Dr. L. J. Grauke for his efforts on providing materials, polishing language and valuable suggestions of manuscript preparation.

Additional files

Additional file 1: A word file with Tables S1-S25.

Additional file 2: A word file with Fig. S1–S14.

Additional file 3: An excel file with DEGs.

Funding

The research was mainly supported by the grant from the National Key R&D Program of China (2018YFD1000604), and partially supported by the grant of the 863 Program from the Chinese Ministry of Science and Technology (2013AA102605), the grant from National Science Foundation of China (31470682, 31670682 and 31570666), the Zhejiang Agriculture (fruit) New Variety Breeding Major Science and Technology Special (2016C02052-13), and the Teacher Professional Development Project of Domestic Visiting Scholar in Zhejiang Province (FX2015040).

Authors' contributions

J.H. and L.X. designed the experiments and managed the project. L.X. conceived the paper and Y.H and L.X. wrote the manuscript. Z.W., L.J.G., R.Z., Q.Z., X.W., G.X., C.X., C.H., R.H., T.F., J.W., C.S. and S.Z. collected samples. Y.H. and Z.W. prepared and purified DNA and RNA samples for whole genome sequencing and RNA-Seq. C.X. estimated genome sizes by flow cytometry analysis. Y.H., L.X., Z.Z., R.Z. and S.Z performed data analyses. L.X., Y.H. and Z.Z. prepared and edited all the figures and tables. J.H., L.X., Y.H, L.J.G., X.W., C.L. and J.R. revised the manuscript and L.J.G., C.L., X.W., J.R. and L.X. polished the language. M.C., Z.C., L.G. and W.J. provided valuable suggestions on the beginning of the project initiation. J.H., Z.W., B.Z. and J.W. initiated and coordinated the project. All authors discuss the results and comment on the manuscript.

Competing financial interests

The authors declare no competing financial interests.

References

1. Manos PE, Stone DE. Evolution, phylogeny, and systematics of the *Juglandaceae*. Annals of the Missouri Botanical Garden. 2001;88:231-269.

2. Lu A, Stone DE, Grauke LJ. Juglandaceae. Flora of China. 1999;4:277-285.

3. Zhang J, Li R, Xiang X, Manchester SR, Li L, Wei W, et. al. Integrated Fossil and Molecular Data Reveal the Biogeographic Diversification of the Eastern Asian-Eastern North American Disjunct Hickory Genus (*Carya* Nutt.). Plos One. 2013;8:e70449.

4. Wen J. Evolution of eastern Asian and eastern North American disjunct distributions of flowering plants. Annu Rev Ecol Syst. 1999;30:421-455.

5. Grauke LJ, Wood BW, Harris MK. Crop vulnerability: *Carya*. HortScience. 2016;51:653-663.

6. Sun Z. and He S. The history, present, and prospect of pecan, in China (*Carya illinoensis*, *Carya cathayensis*, cultivation and breeding). Pecan South. 1982;9:5.

7. Bolling BW, Chen CY, McKay DL, Blumberg JB. Tree nut phytochemicals: composition, antioxidant capacity, bioactivity, impact factors. A systematic review of almonds, Brazils, cashews, hazelnuts, macadamias, pecans, pine nuts, pistachios and walnuts. Nutr Res Rev. 2011;24:244-275.

8. Miraliakbari H, Shahidi F. Antioxidant activity of minor components of tree nut oils. Food Chemistry. 2008;111:421-427.

9. Zhu C, Deng X, Shi F. Evaluation of the antioxidant activity of Chinese Hickory (*Carya cathayensis*) kernel ethanol extraction. Afr J Biotechnol. 2008;7:44-45.

10. USDA Food Composition Databases. https://ndb.nal.usda.gov/. Accessed 2018

11. Venkatachalam M, Kshirsagar HH, Seeram NP, Heber D, Thompson TE, Roux KH, et al. Biochemical composition and immunological comparison of select pecan (Carya illinoinensis (Wangenh.) K. Koch) cultivars. J Agric Food Chem. 2007;55:9899-9907.

12. Hilbig J, Policarpi PB, Grinevicius VMAS, Mota NSRS, Toaldo IM, Luiz MTB, Pedrosa, RC, Block JM. Aqueous extract from Pecan nut [*Carya illinoinensis* (Wangenh) C. Koch] shell show activity against breast cancer cell line MCF-7 and Ehrlich ascites tumor in Balb-C mice. J Ethnopharmacol. 2018;211: 256 -266.

13. National Agricultural Statistics Service. Noncitrus Fruits and Nuts. 2015 Summary. July 2016.

14. Lv Q, Shen Y, Gao,Y, Huang J. Development process, agents and prospect of hickory industry. J Zhejiang A F Univ. 2012;29:97-103.

15. Thompson TE, Grauke LJ. Pecans and other hickories (*Carya*). Acta Hortic. 1991;290:839-906.

16. Wood BW, Payne JA, Grauke LJ. The rise of the U.S. pecan industry. HortScience. 1990;25:721-723.

17. Corsa WP. Nut culture in the United States: embracing native and introduced species. USDA Pomology Div. Govt. Printing Office, Washington D.C. 1896. For archived document, see https://archive.org/details/CAT10504948/page/n1.

18. USDA. World pecan production. Pecan Report: http://www.pecanreport.com/2017-world-pecan-production(2017).

19. Yang J, Zhou F, Xiong L, Mao S, Hu Y, Lu B. Comparison of phenolic compounds, tocopherols, phytosterols and antioxidant potential in Zhejiang pecan (*Carya cathayensis*) at different stir-frying steps. LWT--Food Sci Technol. 2015;62:541-548.

20. Grauke LJ. Hickories. In: Fulbright, Dennis (ed.) Nut Tree Culture in North America. Vol. 1. Northern Nut Growers Assoc., Inc. Pub. 2003. p. 117-166.

21. Zhang B, Wang Z, Jin S, Xia G, Huang Y, Huang J. A pattern of unique embryogenesis occurring via apomixis in *Carya cathayensis*. Biologia Plantarum. 2012;56:620-627.

22. Grauke LJ, Thompson TE. Pecans and hickories. In: Janick J , Moore JN (eds.), Fruit Breeding. Nuts, vol. 3. Wiley, New York. 1996. P. 185-239.

23. Thompson TE. The USDA pecan breeding program. Annual Report of the Northern Nut Growers Association (USA). 1983; 63-66.

24. Gardea AA, Martínez-Téllez MA, Yahia EM. 8–Pecan (*Carya illinoiensis*, (Wangenh.) K. Koch.). In: Yahia E, editors. Postharvest Biology and Technology of Tropical and Subtropical Fruits. mangosteen to White Sapote. 2011. p. 143-165.

25. Liu G, Zhu H, Zang X, Sheng J, Zhou B. Maoshan 1, a new pecan cultivar. J Fruit Sci. 2011;28:1132-1133.

26. Conner PJ. Pecan breeding review. Pecan South. 2012;45:34-44.

27. Grauke LJ, Klein RR, Grusak MA, Klein PE. The forest and the trees: applications for molecular markers in the Repository and Pecan Breeding Programs. Acta Hort. 2015;1070:109-126.

28. Jenkins J, Wilson B, Grimwood J, Schmutz J, Grauke LJ. Towards a reference pecan genome sequence. Acta Hort. 2015;1070:101-108.

29. Thompson TE, Grauke LJ. 'Pawnee' pecan. Journal of American Pomological Society. 2000;20:110-113.

30. Zhang R, Peng F, Li Y. Pecan production in China. Sci Hortic. 2015;197:719-727.

31. Corteolivares J, Phillips GC,Butlernance SA. Somatic embryogenesis from pecan zygotic

embryo explants. Hortscience. 1990;25:983-983.

32. Zhang Q, Hu H, Huang Y, Han K, Xv H, Shen Y, et al. The relationship between developmental stages of zygotic embryos at explanting and embryogenic frequency on hickory (Carya cathayensis Sarg.). Sci Hortic. 2012;139:66-70.

33. Doudna JA, Charpentier E. Genome editing. The new frontier of genome engineering with CRISPR-Cas9. Science. 2014;346:1258096.

34. Parra G, Bradnam K, Korf I. CEGMA: a pipeline to accurately annotate core genes in eukaryotic genomes. Bioinformatics. 2007;23:1061-1067.

35. Simão FA, Waterhouse RM, Ioannidis P, Kriventseva EV, Zdobnov EM. BUSCO: assessing genome assembly and annotation completeness with single-copy orthologs. Bioinformatics. 2015;31:3210.

36. Martínez-García PJ, Crepeau MW, Puiu D, Gonzalez-Ibeas D, Whalen J, Stevens KA, et al. The walnut (Juglans regia) genome sequence reveals diversity in genes coding for the biosynthesis of non-structural polyphenols. Plant J. 2016;87:507-532.

37. Zhang G, Liu X, Quan Z, Cheng S, Xu X, Pan S, et al. Genome sequence of foxtail millet (*Setaria italica*) provides insights into grass evolution and biofuel potential. Nat Biotechnol. 2012;30:549.

38. Mai HD. Die Vietnam-Nuß in Europa. Feddes Repertorium. 1981; 92(5-6):339-385.

39. Takehiko K. Regulation of ribosomal RNA gene copy number and its role in modulating genome integrity and evolutionary adaptability in yeast. Cell Mol Life Sci. 2011;68:1395-1403.

40. Salojärvi J, Smolander OP, Nieminen K, Rajaraman S, Safronov O, Safdari P, et al. Genome sequencing and population genomic analyses provide insights into the adaptive landscape of

silver birch. Nat Genet. 2017;49:904-912.

41. Gong Y, Pegg RB, Carr EC, Parrish DR, Kellett ME, Kerrihard AL. Chemical and nutritive characteristics of tree nut oils available in the U.S. market. Eur. J. Lipid Sci. Technol. 2017;119:1600520.

42. Bourgis F, Kilaru A, Cao X, Ngando-Ebongue GF, Drira N, Ohlrogge JB, et al. Comparative transcriptome and metabolite analysis of oil palm and date palm mesocarp that differ dramatically in carbon partitioning. Proc Natl Acad Sci USA. 2011;108:12527–32.

43. Troncoso-Ponce MA, Kilaru A, Cao X, Durrett TP, Fan J, Jensen JK, et al. Comparative deep transcriptional profiling of four developing oilseeds. Plant J. 2011;68:1014–27.

44. Schmutz J, Cannon SB, Schlueter J, Ma JX, Mitros T, Nelson W, et al. Genome sequence of the palaeopolyploid soybean. Nature. 2010;463:178-183.

45. Huang S, Sirikhachornkit A, Su X, Faris J, Gill B, Haselkorn R, et al. Genes encoding plastid acetyl-CoA carboxylase and 3-phosphoglycerate kinase of the Triticum/Aegilops complex and the evolutionary history of polyploid wheat. Proc Natl Acad Sci USA. 2002;99:8133.

46. Huang J, Zhang T, Zhang Q, Chen M, Wang Z, Zheng B, et al. The mechanism of high contents of oil and oleic acid revealed by transcriptomic and lipidomic analysis during embryogenesis in *Carya cathayensis* Sarg. BMC Genomics. 2016;17:113.

47. Fofana B, Cloutier S, Duguid S, Ching J, Rampitsch C. Gene expression of stearoyl-ACP desaturase and delta 12 fatty acid desaturase 2 is modulated during seed development of flax (*Linum usitatissimum*). Lipids. 2006;41:705-712.

48. Upchurch RG. Fatty acid unsaturation, mobilization, and regulation in the response of plants to stress. Biotechnol Lett. 2008;30:967-977.

49. Wallis JG, Browse J. Mutants of *Arabidopsis* reveal many roles for membrane lipids. Prog Lipid Res. 2002;41:254-278.

50. Haslam E. Plant polyphenols (syn. vegetable tannins) and chemical defense-A reappraisal. J. Chem. Ecol. 1988;14:1789-1805.

51. Lima GPP, Vianello F, Corrêa CR, Campos RADS, Borguini MG. Polyphenols in Fruits and Vegetables and Its Effect on Human Health. Food Nutr Sci. 2014;5:1065-1082.

52. Orain R, Lebreton V, Ermolli ER, Combourieu-Nebout N, Sémah AM. *Carya* as marker for tree refuges in southern Italy (Boiano basin) at the Middle Pleistocene. Palaeogeogr Palaeoclimatol Palaeoecol. 2013;369:295-302.

53. Yang J, Zhou F, Xiong L, Mao S, Hu Y, Lu B. Comparison of phenolic compounds, tocopherols, phytosterols and antioxidant potential in Zhejiang pecan (*Carya cathayensis*) at different stir-frying steps. LWT--Food Sci Technol. 2015;62:541-548.

54. Liu Y, Studies on identification and pollution-free control technique of main diseases and pests on Chinese hickory (*Carya cathayensis*), Anhui Agriculture University. Anhui Agriculture University, Hefei, China. 2014.

55. Sparks D. Adaptability of pecan as a species. HortScience. 2005;40:1175-1189.

56. Van ZJ. Cultivation of pecan nuts in South Africa. Vegetables & Fruit. 2000.

57. Kumar RMS, Ji G, Guo H, Zhao L, Zheng B. Over-expression of a grafting-responsive gene from hickory increases abiotic stress tolerance in arabidopsis. Plant Cell Rep. 2018;37: 541-552.

58. Sanyal A, Linder CR. Plasticity and constraints on fatty acid composition in the phospholipids and triacylglycerols of *Arabidopsis* accessions grown at different temperatures. BMC Plant Biol. 2013;13:63.

59. Linder CR. Adaptive Evolution of Seed Oils in Plants: Accounting for the Biogeographic Distribution of Saturated and Unsaturated Fatty Acids in Seed Oils. Am Nat. 2000;156:442-458.

60. Klinkenberg J, Deeken R. Two fatty acid desaturases, stearoyl-acyl carrier protein δ9-desaturase6 and fatty acid desaturase3, are involved in drought and hypoxia stress signaling in *Arabidopsis* crown galls. Plant Physiol. 2014;164:570-583.

61. Huang R, Huang Y, Sun Z, Huang J, Wang Z. Transcriptome analysisof genes involved in lipid biosynthesis in the developing embryo of pecan (*Carya illinoinensis*). J Agric Food Chem. 2017;65:4223-4236.

62. Porebski S, Bailey LG, Baum BR. Modification of a CTAB DNA extraction protocol for plants containing high polysaccharide and polyphenol components. Plant Mol Biol Rep. 1997;15:8-15.

63. Chaisson MJ, Glenn T. Mapping single molecule sequencing reads using basic local alignment with successive refinement (BLASR): application and theory. BMC Bioinformatics. 2012;13:238.

64. Li R, Fan W, Tian G, Zhu H, He L, Cai J, et al. The sequence and de novo assembly of the giant panda genome. Nature. 2010;463:311-317.

65. Huang Y; Xiao L; Zhang Z; Zhang R; Wang Z; Huang C; Huang R; Luan Y; Fan T; Wang J; Shen C; Zhang S; Wang X; Randall J; Zheng B; Wu J; Zhang Q; Xia G; Xu C; Chen M; Zhang L; Jiang W; Gao L; Chen Z; Leslie CA; Grauke LJ; Huang J (2019): Supporting data for "The genomes of pecan and Chinese hickory provide insights into Carya evolution and nut nutrition" GigaScience Database. http://dx.doi.org/10.5524/100571.

66. Kajitani R, Toshimoto K, Noguchi H, Toyoda A, Ogura Y, Okuno M, et al. Efficient de novo

assembly of highly heterozygous genomes from whole-genome shotgun short reads. Genome Res. 2014;24:1384-1395.

67. English AC, Richards S, Han Y, Wang M, Vee V, Qu J, Qin X, Muzny DM, Reid JG, Worley KC, Gibbs RA. Mind the gap: upgrading genomes with Pacific Biosciences RS long-read sequencing technology. PLoS One. 2012;7:e47768.

68. Luo R, Liu B, Xie Y, Li Z, Huang W, Yuan J. SOAPdenovo2: an empirically improved memory-efficient short-read de novo assembler. Gigascience. 2012;1:18.

69. Li H. Aligning sequence reads, clone sequences and assembly contigs with BWA mem. arXiv preprint arXiv. 2013;1303:3997.

70. Li H, Handsaker B, Wysoker A, Fennell T, Ruan J, Homer N, et al. The Sequence Alignment/Map(SAM) Format and SAMtools. Transplant Proc. 2009;19:1653-1654.

71. Grabherr MG, Haas BJ, Yassour M, Levin JZ, Thompson DA, Amit I, et al. Trinity: reconstructing a full-length transcriptome without a genome from rna-seq data. Nat. Biotechnol. 2011;29:644.

72. Kent WJ. BLAT--the BLAST-like alignment tool. Genome Res. 2002;12:656-664.

73. Hosseini P, Tremblay A, Matthews BF, and Alkharouf NW. An efficient annotation and gene-expression derivation tool for illumina solexa datasets. BMC Res Notes. 2010;3:1-7.

74. Andrews S. FastQC software. https://www.bioinformatics.babraham.ac.uk/projects/fastqc/.

75. RepeatMolder software. http://www.repeatmasker.org/RepeatModeler/.

76. RepeatMasker software. http://www.repeatmasker.org/

77. Benson G. Tandem repeats finder: a program to analyze DNA sequences. Nucleic Acids Res. 1999;27:573-580.

78. Ensembl Database. http://www.ensembl.org/index.html.

79. JGI Genome Portal Database. https://genome.jgi.doe.gov/portal/.

80. Yu X, Zheng H, Wang J, Wang W, Su B. Detecting lineage-specific adaptive evolution of brain-expressed genes in human using rhesus macaque as outgroup. Genomics. 2006;88:745-751.

81. Birney E, Durbin R. Using GeneWise in the Drosophila Annotation Experiment. Genome Res. 2000;10:547-548.

82. Trapnell C, Pachter L, Salzberg SL. TopHat: discovering splice junctions with RNA-Seq. Bioinformatics. 2009;25:1105-1111.

83. Trapnell C, Williams BA, Pertea G, Mortazavi A, Kwan G, van Baren MJ, Salzberg SL, Wold BJ, Pachter L. Transcript assembly and quantification by RNA-Seq reveals unannotated transcripts and isoform switching during cell differentiation. Nat Biotechnol. 2010;28:511-515.

84. Campbell MA, Hass BJ, Hamilton JP, Mount SM, Buell CR. Comprehensive analysis of alternative splicing in rice and comparative analyses with Arabidopsis. BMC Genomics. 2006;7:327.

85. Stanke M, Steinkamp R, Waack S, Morgenstern B. AUGUSTUS: a web server for gene finding in eukaryotes. Nucleic Acids Res. 2004;32:309-312.

86. Aggarwal G, Ramaswamy R. Ab initio gene identification: prokaryote genome annotation with GeneScan and GLIMMER. J Biosci. 2002;27:7-14.

87. Majoros WH, Pertea M, Salzberg SL. TigrScan and GlimmerHMM: two open source ab initio eukaryotic gene-finders. Bioinformatics. 2004;20:2878-2879.

88. Parra G, Blanco E, Guigó R. GeneID in Drosophila. Genome Res. 2000;10:511.

89. Bromberg Y, Rost B. SNAP: predict effect of non-synonymous polymorphisms on function.

Nucleic Acids Res. 2007;35:3823.

90. Haas BJ, Salzberg SL, Wei Z, Pertea M, Allen JE, Orvis J, et al. Automated eukaryotic gene structure annotation using evidencemodeler and the program to assemble spliced alignments. Genome Biol. 2008;9:R7.

91. Gish W, States DJ. Identification of protein coding regions by database similarity search. Nat Genet. 1993;3:266-272.

92. Hunter S, Apweiler R, Attwood TK, Bairoch A, Bateman A, Binns D, et al. InterPro: the integrative protein signature database. Nucleic Acids Res. 2009;37:D211-D215.

93. Finn RD, Bateman A, Clements J, Coggill P, Eberhardt RY, Eddy SR, et al. Pfam: the protein families database. Nat Genet. 2014;42:D222.

94. Quevillon E, Silventoinen V, Pillai S, Harte N, Mulder N, Apweiler R, et al. InterProScan: protein domains identifier. Nucleic Acids Res. 2005;33:116-120.

95. Finn RD, Clements J, Eddy SR. HMMER web server: interactive sequence similarity searching. Nucleic Acids Res. 2011;39:29-37.

96. Lowe TM, Eddy SR. tRNAscan-SE: a program for improved detection of transfer RNA genes in genomic sequence. Nucleic Acids Res. 1997;25:955-964.

97. Griffithsjones S, Moxon S, Marshall M, Khanna A, Eddy SR, Bateman A. Rfam: annotating non-coding RNAs in complete genomes. Nucleic Acids Res. 2005;33:D121.

98. Nawrocki EP, Kolbe DL, Eddy SR. Infernal 1.0: inference of RNA alignments. Bioinformatics. 2009;25:1335.

99. Bethesda, MD: Trivial HTTP, RFC2169. ftp://ftp.ncbi.nlm.nih.gov/genomes/all/GCF/001/411/555/GCF_001411555.1_wgs.5d/(2018).

Accessed 4 Nov 2016.

100. David DM, Shu S, Howson R, Neupane R, Hayes RD, Fazo J, Mitros T, Dirks W, Hellsten U, Putnam N, Rokhsar DS. Phytozome: a comparative platform for green plant genomics. Nucleic Acids Res. 2012;40:D1178-1186.

101. Li L, Stoeckert CJ Jr, Roos DS. OrthoMCL: identification of ortholog groups for eukaryotic genomes. Genome Res. 2003;13:2178-2189.

102. Edgar RC. MUSCLE: multiple sequence alignment with high accuracy and high throughput. Nucleic Acids Res. 2004;32:1792-1797.

103. Stamatakis A. RAxML version 8: a tool for phylogenetic analysis and post-analysis of large phylogenies. Bioinformatics. 2014;30:1312-1313.

104. Yang Z. PAML 4: phylogenetic analysis by maximum likelihood. Mol Biol Evol. 2007;24:1586-1591.

105. Hedges SB, Dudley J, Kumar S. TimeTree: a public knowledge-base of divergence times among organisms. Bioinformatics. 2006;22:2971-2972.

106. Chinese Virtual Herbarium. Institute of Botany, the Chinese Academy of Sciences. 2004. http://www.cvh.ac.cn/. Accessed 2004.

107. National Specimen information infrastructure. http://mnh.scu.edu.cn/.

108. National Resources Conservation Service. USDA. https://plants.usda.gov/java/.

109. ArcGIS software. esri. https://www.esri.com/.

110. Vilella AJ, Severin J, Ureta-Vidal A, Heng L, Durbin R, and Birney. EnsemblCompara GeneTrees: Complete, duplication-aware phylogenetic trees in vertebrates. Genome Res. 2009; 19:327-335.

111. Tang H, Bowers JE, Wang X, Ming R, Alam M, Paterson AH. Synteny and collinearity in plant genomes. Science. 2008;320:486-488.

112. The Potato Genome Sequencing Consortium. Genome sequence and analysis of the tuber crop potato. Nature.2011; 475:189-195.

113. Ellinghaus D, Kurtz S, Willhoeft U. LTRharvest, an efficient and flexible software for *de novo* detection of LTR retrotransposons. BMC Bioinformatics. 2008;9:18.

114. Steinbiss S, Willhoeft U, Gremme G, Kurtz S. Fine-grained annotation and classification of *de novo* predicted LTR retrotransposons. Nucleic Acids Res. 2009;37:7002-13.

115. Kumar S, Stecher G, Tamura K. MEGA7: Molecular Evolutionary Genetics Analysis Version 7.0 for Bigger Datasets. Mol Biol Evol. 2016;33:1870.

116. Hu B, Jin J, Guo AY, Zhang H, Luo J, Gao G. GSDS 2.0: an upgraded gene feature visualization server. Bioinformatics. 2015; 31:1296-1297.

117. Li R, Li Y, Kristiansen K, Wang J. Soap: short oligonucleotide alignment program. Bioinformatics. 2008;24:713-714.

118. Love MI, Huber W, Anders S. Moderated estimation of fold change and dispersion for RNA-seq data with DESeq2. Genome Biol. 2014;15:550.

119. Ashburner M, Ball CA, Blake JA, Botstein D, Butler H, Cherry JM, et al. Gene ontology: tool for the unification of biology. Nat Genet. 2000;25:25-29.

120. Young MD, Wakefield MJ, Smyth GK, and Oshlack A. Gene ontology analysis for RNA-seq: accounting for selection bias. Genome Biology. 2010;11:R14.

121. Kanehisa M, Goto S, Sato Y, Furumichi M, Tanabe M. KEGG for integration and interpretation of large-scale molecular data sets. Nucleic Acids Res. 2012;40:D109-114.

122. Xie C, Mao X, Huang J, Ding Y, Wu J, Dong S, Kong L, Gao G, Li CY, and Wei L. KOBAS2.0: a web server for annotation and identification of enriched pathways and diseases. Nucleic Acids Res. 2011;39:W316-322.

123. Kolde R. Pheatmap: pretty heatmaps. R package version. 2012;61.

Table 1. Assembly summary of pecan ('Pawnee') and Chinese hickory (ZAFU-1) genomes.

	ZAFU-1	'Pawnee'
Estimated genome size (Mb) *	721.33	649.75
Total assembly (Mb)	706.43	651.31
Longest scaffold (Mb)	4.95	4.92
Number of contigs **	15,789	17,542
N50 contig length (Kb)	101.58	77.23
N50 contig count	1,879	2,388
Number of scaffolds **	5,449	3,860
N50 scaffold length (Mb)	1.22	1.08
N50 scaffold count	174	188
N90 scaffold length (Kb)	137.39	210.68
N90 scaffold count	732	682
Missing bases (%)	1.61	0
Protein-coding genes	32,907	31,075
Repeat sequence (Mb/% ***)	381.01/53.67	334.55/50.43
micro RNAs	373	378

tRNAs	558	571
rRNAs	362	198

* show the revised genome size estimation; ** show the number of contig or scaffold >= 2 kb; *** show percentage of assembled genomes.

Figures and legends

Figure 1. Evolutionary analyses of the *Carya* genus and the genomes of pecan and Chinese hickory. (a) Phylogeny of pecan and Chinese hickory and 12 other genome-sequenced species in Rosids. (b) Phylogeny of 16 *Carya* species (ML tree) with two *Juglans* species as out-group. (c) Geographical distribution of both fossil and extant *Carya* species. (d) Whole genome duplication and speciation in genomes of pecan, Chinese hickories and walnut based on 4DTv. (e) Syntenic analysis of pecan, Chinese hickory and walnut. Only the scaffolds with syntenic relationship were shown (including 10 longest scaffold with syntenic blocks). (f) A Venn diagram illustrating shared and specific gene families in pecan, Chinese hickory, *Juglans regia* and other representative species in Fagales, Fabales and Rosales.

Figure 2. Selected stress-associated genes in pecan and Chinese hickory. (a) Statistics of important drought-associated genes in 6 sequenced grass genomes. (b) ML tree of PYLs in pecan, Chinese hickory and *Arabidopsis*. (c) ML tree of SnRK2 genes in pecan, Chinese hickory and *Arabidopsis*. (d) ML tree of LEA protein genes in pecan, Chinese hickory and *Arabidopsis*.

Figure 3. Fruits and seeds, oil metabolism overview and the detailed analyses of expanded key gene families. (a) The fruits and seeds of pecan and Chinese hickory. (b) Oil biosynthesis pathway, combined with the gene copy number and transcription abundance shown by boxes in pecan (upper boxes) and Chinese hickory (lower boxes). (c-d) Comparative analyses on gene structure and evolution of the rating-limit enzyme ACCases and key enzyme SADs in pecan and Chinese hickory against those in *Arabidopsis*.

Figure 4. Key genes involved in polyphenol metabolism in pecan and Chinese hickory. (a) Polyphenol biosynthesis pathway shows the gene copy number encoding key enzymes and transcription factors by solid dots or number in pecan (red) and Chinese hickory (purple). (b) Heatmap of gene expression profiles of key genes during embryo development in pecan and Chinese hickory. Gray blocks indicate the missing data. (c-d) Diagrams showing the key steps in the biosynthesis of arginine (c) and thiamine (d). (e) Gene copy number of enzymes involved in the biosynthesis of arginine and vitamin B1 in *Arabidopsis* and three *Juglandaceae* species.

Figure 1

Click here to access/download;Figure;Fig. 1_2019.pdf

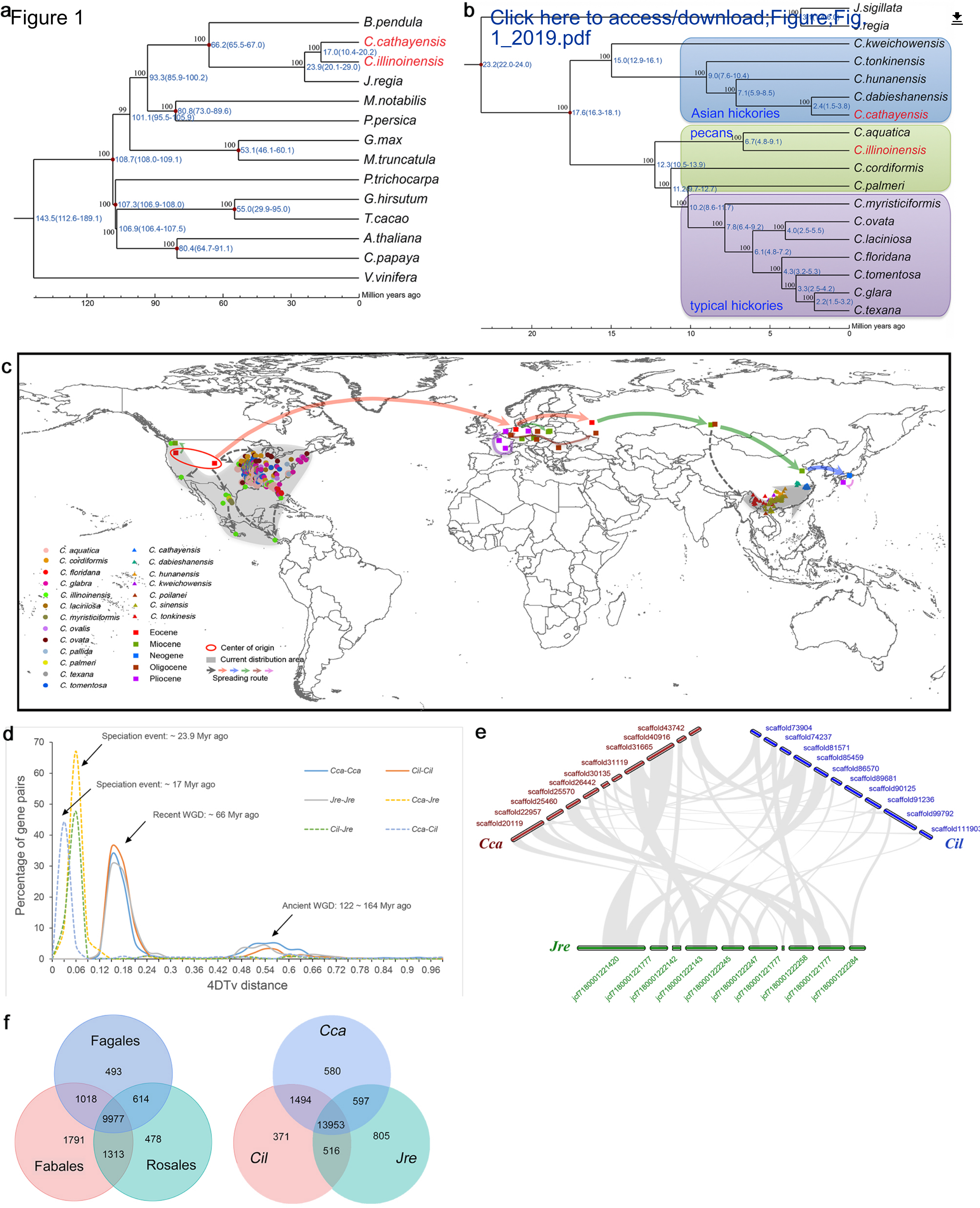

Figure 2

Click here to access/download;Figure;Fig. 2.pdf

a

Gene	Enzyme	*Ath*	*Cca*	*Cil*
ABA1 (ZEP)	1.14.13.90	1	2	2
VDE1	1.10.99.3	1	1	1
ABA2 (SDR)	1.1.1.288	1 (5)	2 (14)	1 (17)
ABA3 (MOSC)	2.8.1.9	1 (8)	1 (5)	1 (4)
ABA4/SDR1	5.3.99.9	1	1	1
NCED/VP14	1.13.11.51	**7**	**7**	**8**
AAO (AAO3)	1.2.3.7 (1.2.3.14)	4 (1)	2 (1)	2 (1)
CYP707A	1.14.13.93	4	**6**	**6**
PYLs		**14**	**14**	**14**
PP2CA	3.1.3.16	**9**	**12**	**10**
SnRK2	2.7.11.1	**10**	**13**	**12**
SOD	1.15.1.1	**9**	**11**	**11**
POD	1.11.1.7	**76**	**81**	**81**
CAT	1.11.1.21	3	2	3
GST	2.5.1.18	53	31	22
PLD	3.1.4.4	12	9	7
LEA		**51**	**82**	**72**
WRKY		72	69	71
NAC		101	99	95
	CC-NBS	**4**	**22**	**15**
	CC-NBS-LRR	**35**	**55**	**52**
	CC-TIR-NBS-LRR	0	0	1
	LRR-NBS-LRR	0	2	2
	LRR-TIR-NBS-LRR	0	0	2
	NBS	**3**	**102**	**92**
	NBS-LRR	**22**	**171**	**159**
	NBS-LRR-NBS	0	0	2
	NBS-LRR-TIR	1	1	0
	NBS-LRR-NBS-LRR	0	0	3
	NBS-LRR-TIR-NBS	0	0	1
R gene	NBS-LRR-TIR-NBS-LRR	0	0	2
	NBS-TIR-NBS-LRR	0	0	1
	TIR-CC-NBS-LRR	2	1	1
	TIR-NBS	**16**	**30**	**28**
	TIR-NBS-LRR	80	99	91
	TIR-NBS-LRR-NBS-LRR	0	2	1
	TIR-NBS-LRR-NBS-TIR-NBS-LRR	0	1	0
	TIR-NBS-LRR-TIR	1	2	1
	TIR-NBS-LRR-TIR-NBS-LRR	1	2	0
	TIR-NBS-TIR-NBS-LRR	2	0	0
	TIR-NBS-TIR-NBS	0	1	0
	total	**167**	**491**	**454**

b

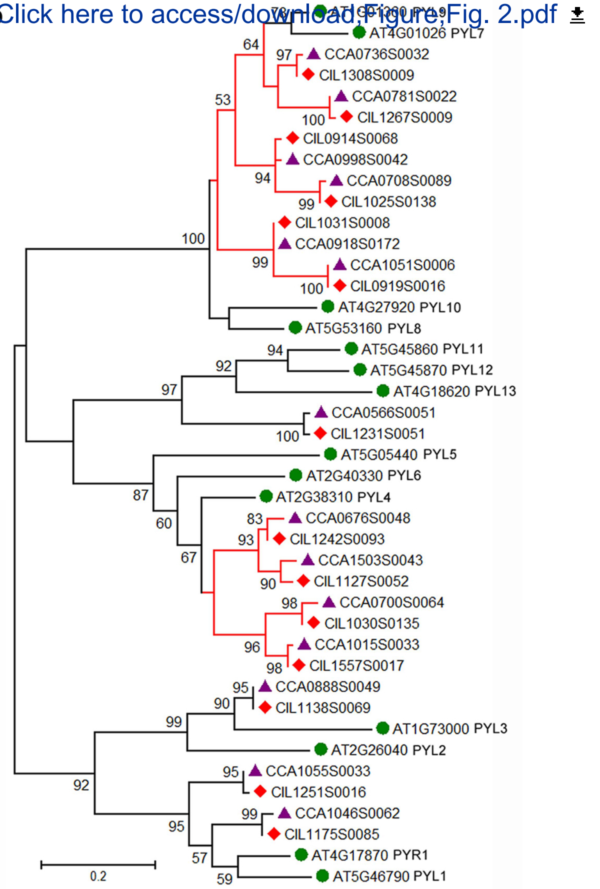

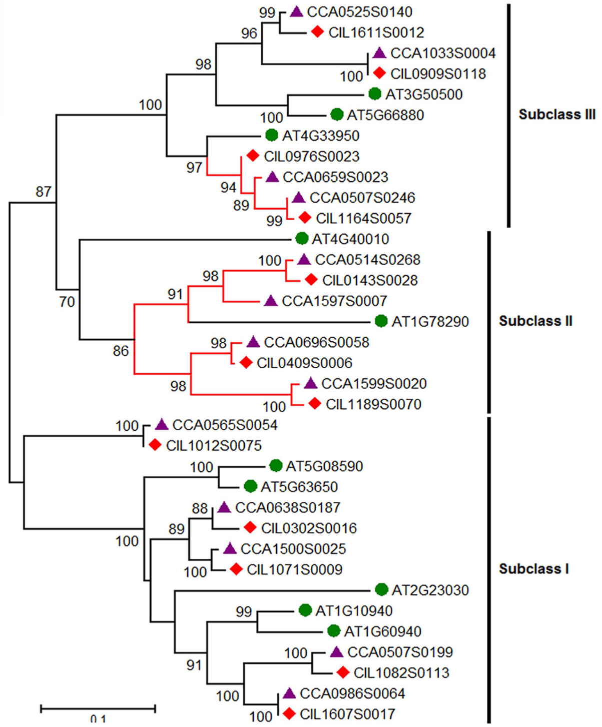

d

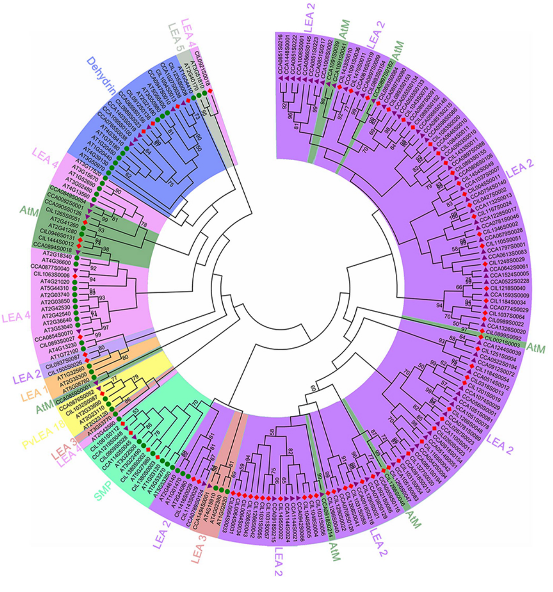

Figure 3 Click here to access/download;Figure;Figure 3.png

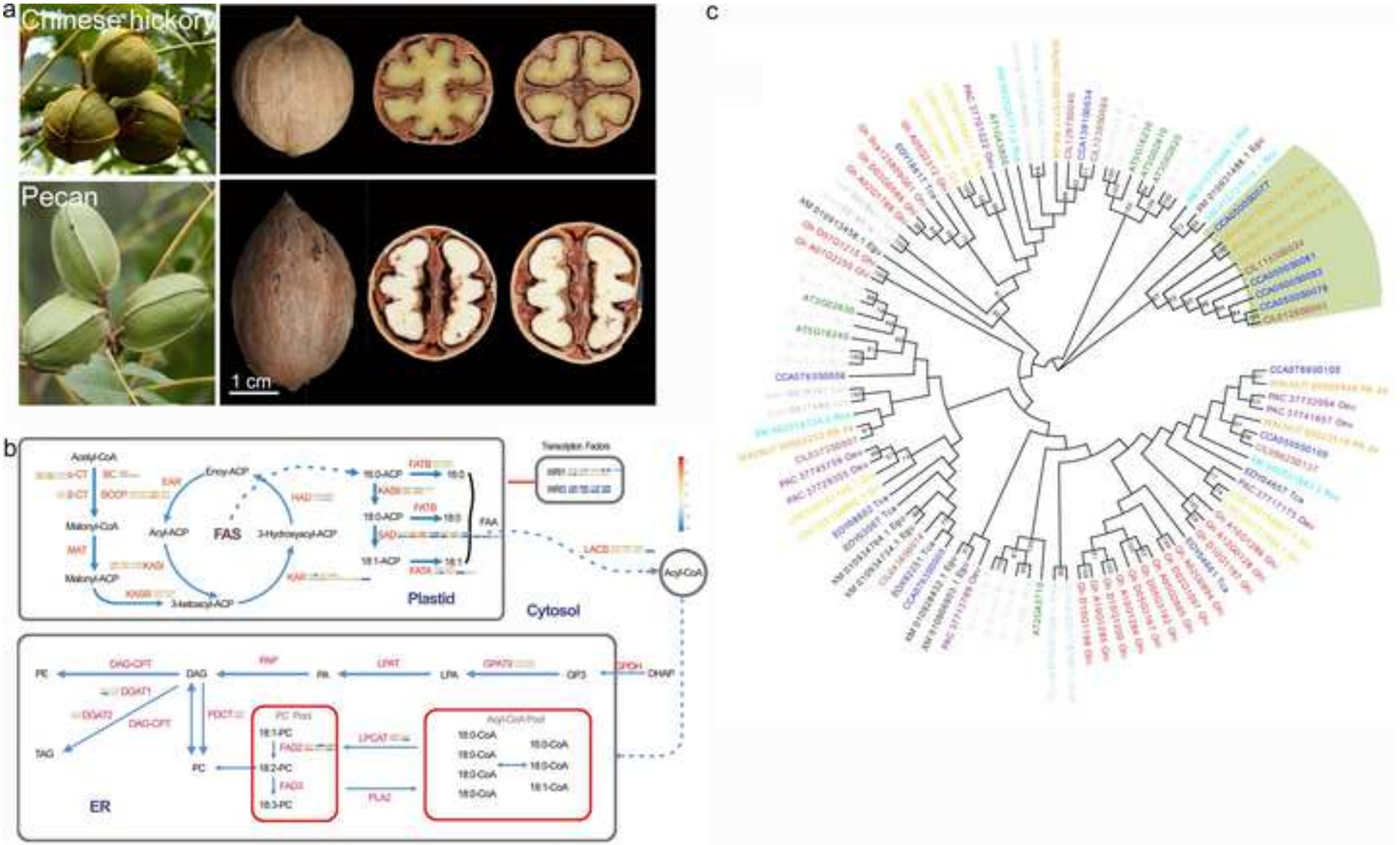

Figure 4

Click here to access/download;Figure;Figure 4.png

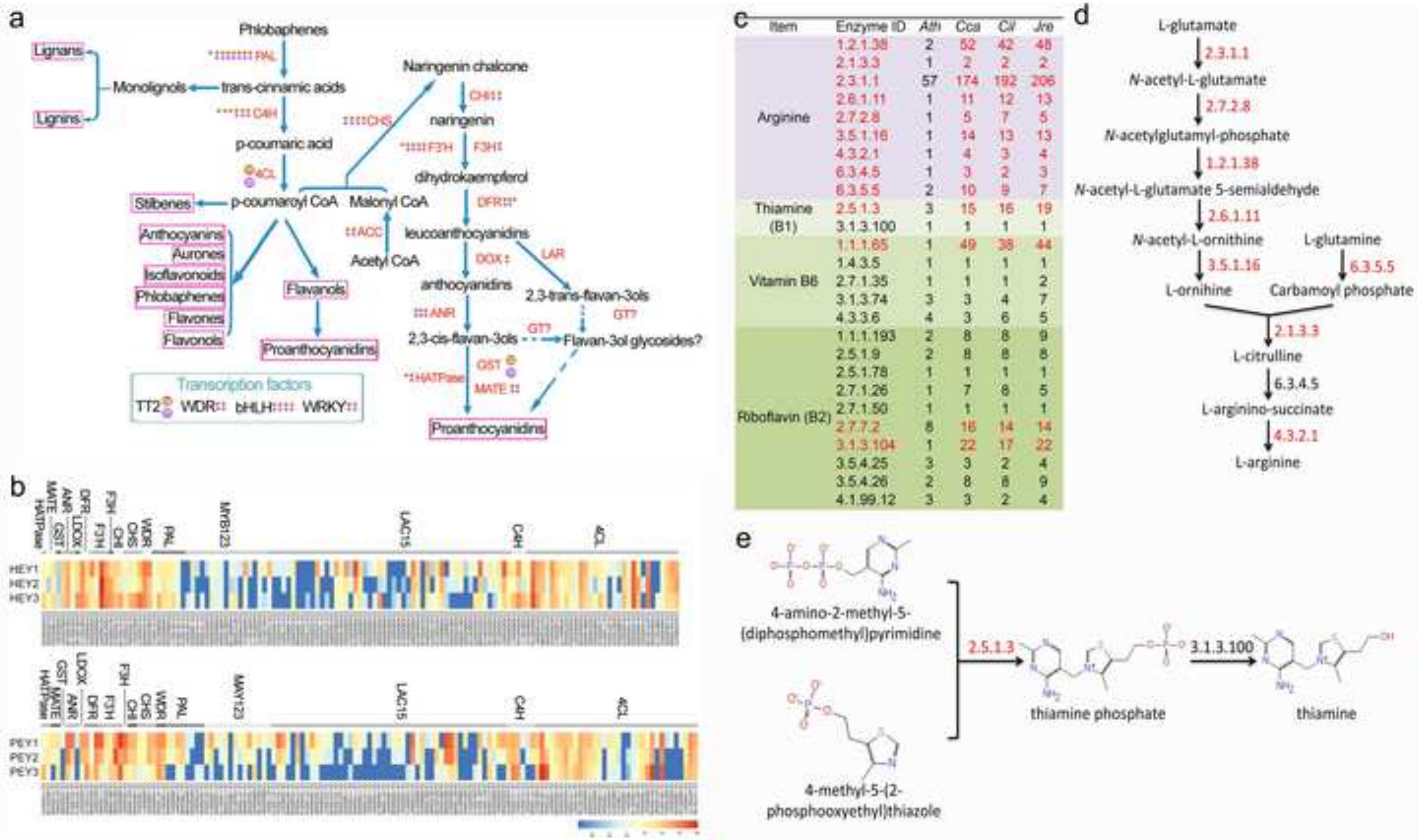

Additional file 1-Tables

Click here to access/download
Supplementary Material
Additional file 2.docx

Additional file 2-Figures

Click here to access/download
Supplementary Material
Additional file 1.docx

Additional file 3-DEG list

Click here to access/download
Supplementary Material
Additional file 3.xlsx
