# Supplementary material for: The genomes of pecan and Chinese hickory provide insights into Carya evolution and nut nutrition
Source: Gigascience. 2019 May 2;8(5):giz036. doi: 10.1093/gigascience/giz036 (PMC6497033; doi:10.1093/gigascience/giz036)
Supplement: Response_to_Reviewer_Comments_Original_Submission.pdf [file giz036_response_to_reviewer_comments_original_submission.pdf]

Author's Response To Reviewer Comments

Close

Dear Professor, Dr. Laurie Goodman, Editor-in-Chief of GigaScience:
We thank for accepting our manuscript (GIGA-D-18-00185) for publication in GigaScience, contingent on approval of major revisions. We also thank the reviewers for their valuable comments and suggestions for improving our manuscript. We have revised the fully based on reviewers' comments and suggestions (shown as red text in the main document and in additional supplementary files). In the cover letter, we provide responses to the reviewers' comments and suggestions point by point.
To publish our research results in the prestigious Journal GigaScience would be a great honor. We hope the revisions incorporated here allow the manuscript to be accepted for publication in GigaScience.

With Best Regards

Yours Sincerely

Corresponding authors
Drs. Jianqin Huang, Lihong Xiao and L.J. Grauke

The following pages are the responses to the reviewers' comments and suggestions.

Responses to the reviewers' comments and suggestions

Responses to Reviewer #1
We are grateful to reviewer #1's for his valuable comments and kind suggestions for our manuscript. We have revised our manuscript on the full consideration of referee #1's comments and suggestions.
The following are our responses (following the symbol • and marked with blue letters) to referee #1's comments and suggestions (following each number).

1. While not a huge problem to readability, a few English grammar issues remain. The manuscript would benefit from a final editing round by a native English speaker.
• We are grateful to reviewer #1 for raising this question. Dr. L. J. Grauke, one of our corresponding authors, have checked and revised the language carefully in the revised manuscript.

2. Brief descriptions for supplementary tables and figures would help with clarity. Additional File 1, S6 and S7 titles are missing word "of". Figure S2 is missing a label for the green line (presumably C.il).
• Thanks for reviewer #1's suggestions. We have corrected the errors of Tables S6 and S7 (numbered as Table S7 and S8, now) titles in additional file 1 (please see page 5 of Additional file 1). As for Figure

S2, the missed label for the green line is indeed C. illinoiensis and we have added (please see page 1 of Additional file 2).

3. 5S rRNAs are reported as having a larger number in ZAFU than Pawnee - how was this determined? As 5S rRNAs are usually present in large tandem arrays with little to no variation, I wouldn't expect an assembly to yield a very accurate count of rRNAs by itself.

• Thanks to reviewer #1 for paying attention to this question. As the reviewer #1 said that "5S rRNAs are usually present in large tandem arrays with little to no variation, I wouldn't expect an assembly to yield a very accurate count of rRNAs by itself". We also note the difference of 5S rRNAs between the two species. To check the correctness of the annotation, we re-predict non-coding RNAs in the assemblies of pecan and Chinese hickory as followings:
i. tRNA, predicted by searching the assemblies using tRNAscan-SE software and setting E-value as 1e-10 ; ii. miRNA and snRNA, aligning the assembled genome sequences to Rfam database (http://rfam.xfam.org) using INFERNAL software and setting E-value as 0.0001; iii. rRNA, predicted by aligning the rRNAs from Arabidopsis and rice against our assembled genomes using blastN and setting E-value as1e-10.
From the newly prediction, we found that some of the numbers of all predicted categories changed slightly but not significant. We think it probably results from the update of database that was used as references, because the previous prediction has been done for nearly two years. Both previous and newly predictions were shown below and the new Table S15 can also been found in the revised Additional file 1.
Table S14 Statistics of non-coding RNA in pecan and Chinese hickory genomes. (Previous)
Type Copy (w*) Average length (bp) Total length (bp) % of genome
Cil Cca Cil Cca Cil Cca Cil Cca
miRNA 378 373 120.95 128.28 45719 47847 0.006891 0.006740
tRNA 571 558 74.80 74.92 42709 41804 0.006438 0.005888
rRNA rRNA 198 362 192.94 145.23 38202 52575 0.005758 0.007406
18S 61 46 374.61 366.37 22851 16853 0.003444 0.002374
28S 29 34 141.90 125.62 4115 4271 0.00062 0.000602
5.8S 11 12 151 141.58 1661 1699 0.00025 0.000239
5S 97 270 98.71 110.19 9575 29752 0.001443 0.004191
snRNA snRNA 886 1191 111.54 109.13 98826 129973 0.014896 0.018308
CD-box 633 961 104.01 103.61 65840 99572 0.009924 0.014026
HACA-box 83 75 125.71 126.12 10434 9459 0.001573 0.001332
splicing 169 154 132.67 135.14 22422 20812 0.00338 0.002932
*w indicates annotation at genome-wide level.

Table S15 Statistics of non-coding RNA in pecan and Chinese hickory genomes. (New)
Type Copy (w*) Average length (bp) Total length (bp) % of genome
Cil Cca Cil Cca Cil Cca Cil Cca
miRNA 378 373 120.95 128.28 45719 47847 0.006891 0.006740
tRNA 571 558 74.80 74.92 42709 41804 0.006438 0.005888
rRNA rRNA 193 229 196.77 176.34 37976 40381 0.005724 0.005688
18S 59 29 385.88 533.17 22767 15462 0.003432 0.002178
28S 29 18 141.90 171.11 4115 3080 0.000620 0.000434
5.8S 11 11 151 149.91 1661 1649 0.000250 0.000232
5S 94 171 100.35 118.07 9433 20190 0.001422 0.002844
snRNA snRNA 884 956 111.69 114.56 98735 109524 0.014883 0.015427
CD-box 632 767 104.11 108.31 65796 83072 0.009918 0.011701
HACA-box 83 66 125.71 129.94 10434 8576 0.001573 0.001208
splicing 168 122 133.18 145.46 22375 17746 0.003373 0.002500
*w indicates annotation at genome-wide level.

4. Did the authors look for or extract the mitochondria or chloroplast genomes? These would likely have been captured in scaffolds during assembly.

• We thank to reviewer #1 for raising this question. As the reviewer #1 mentioned that the raw sequencing data do include reads from mitochondria or chloroplast genomes. We have filtered out the reads and obtained the chloroplast genome sequences and annotations before scaffolding.
But unfortunately, these assemblies and annotations showed significant difference with our assemblies from other two varieties of pecan (unpublished data). To confirm if or not the differences are true will need extensive experiments and more time. Therefore, we didn't include the chloroplast genomes in this manuscript.

As for the mitochondria genomes, assembling is still in process because of the complicity of the genomes themselves.

5. Although this is background material and not the point of the paper, I'm confused by the apomixis part of the introduction. The statement "nucellar embryony that demonstrates remarkable resistance to fungal diseases" - do you mean that the trait of disease resistance can be passed down easily through nucellar embryony or that nucellar embryony actual functions in preventing disease directy? Apomixis is mentioned again as very good for breeding, but I would argue it might be good for passing down traits in production but terrible for breeding (no recombination, right?). Also, apoximis is misspelled on Page 8 Line 45.

● We thank to reviewer #1 for pointed out this question. We checked the statement in our manuscript and found that it should be an editing mistake during we prepared the manuscript, which might lead reader misunderstanding. According to the context, we wanted to express two points in the paragraph: i. Compared with pecans, Chinese hickory has remarkable resistance to fungal diseases such as pecan scab; ii. Except for sexual reproduction, Chinese hickory has nucellar embryony (apomixis), one of the main ways of reproduction in this species.

We also agree the reviewer #1's argument that "apomixes would be very valuable for for passing down the disease-resistant trait in production". In the revised manuscript, we have addressed the two points. Please see Page 6, Line 17-21.

6. Data Description - As this is a required section by GigaScience, I don't think it's appropriate to ignore it (currently it says only to refer to the methods). Here's what it's supposed to contain: "A statement providing background and purpose for collection of these data should be presented for readers without specialist knowledge in that area. A brief description of the protocol for data collection, data curation and quality control, as well as potential uses should be included, as well as outlining how the data can be accessed if it is not deposited in our repository."

● So sorry for our neglect on this point that might lead reader misunderstanding. We must express our great gratitude to reviewer #1 for his reminding us on this point. In the revised manuscript, a brief description of the protocol for data collection, data curation and quality control, as well as potential uses and outlining how the data can be accessed were included. Please see the revised Materials and Methods section on Page 25 and Data Description on Page 8-9 as well as "Avaiabliity of data and metarials" in the revised manuscript.

Data description: To obtain the whole genome sequences of pecan and Chinese hickory genomes, genomic DNA was extracted from the leaf tissues of 'Pawnee' and ZAFU-1 using the cetyltrimethylammonium bromide (CTAB) method. Paired-end libraries with insert sizes ranging from 250 bp to 500 bp and mate pair libraries with insert sizes of 2 kb and 20 kb were constructed according to the manufacturer's instructions (Illumina, San Diego, CA). All constructed libraries were sequenced on Illumina Hiseq X-ten. In addition, Single-molecule real-time (SMRT) sequencing of long reads on a PacBio RS II platform (Pacific Biosciences, USA) was used to assist the subsequent de novo genome assembly process, a 20-kb insert size SMRTbell library was prepared following the manufacturer's protocol (PacBio, CA, USA). Then, these libraries were sequenced on PacBio RS II platform (Pacific Biosciences, USA) using the P6 polymerase/C4 chemistry combination, based on the manufacturer's procedure (Pacific Biosciences, CA, USA). Sequencing statistics for all libraries are outlined in Table S1. In total, about 157 Gb and 161 Gb reads were generated on Illumina platforms, and 22 Gb and 26 Gb reads were generated on PacBio platforms of Carya cathayensis and Carya illinoinensis. Quality control involved the following steps: (1) removing reads with >= 10% unidentified nucleotides (N); (2) removing reads with > 20% bases having Phred quality < 5; (3) removing reads with >10 nt aligned to the adapter, allowing <= 10% mismatches; (4) removing putative PCR duplicates generated by PCR amplification during the library construction process (i.e. read 1 and 2 of two paired-end reads that were completely identical). Finally, about 123 Gb and 135 Gb of Illumina clean data and 21.7 Gb and 25.8 Gb PacBio clean data were obtained for the de novo assembly of the ZAFU-1 and 'Pawnee' genome, respectively. (Please see Page 9, the first line)

7. Boea hygrometrica pops up as a comparator in a few places, particularly in the transposable elements. But that is an asterid, so it's really not a particularly compelling comparator for a rosid. The comparison to other species either needs to span a wide range of plants from all clades or focus on reference genomes from more closely related rosids (walnut, peach, poplar, etc)

● We agree with the reviewer #1s' suggestion that conducting comparative genome analysis using genomes from more closely related rosids, such as walnut, peach and poplar instead of Boea hygrometrica. We fully considered the reviewer #1's suggestions and removed comparison using B. hygrometrica, instead, we included the genomes of grape and birch as references in the revised manuscript for analyses of phylogeny (Figure 1a in the main text, Fig. S6 in Additional file 2), gene

family expansion and contraction, and divergence time estimation. As for the transposable elements, we cited the data from genomes of walnut, grape and poplar (Martinez-Garcia et al 2016 and reference therein). For the details, please see Page 10, Line 14-17.

8. The walnut genome is not mentioned or cited (Martinez-Garcia et al 2016), despite being used extensively as a comparator. That paper put the Juglandaceae WGD at 60MYA, considerably older than this estimate. These things are not exact, but a brief mention that the WGD was previously found in walnut and that the estimates diverge a bit would be good.
● Sorry for missed the citation of walnut genome in the last submitted manuscript. We should express our grateful to reviewer #1 for raising this question. In the original manuscript, we do include this citation but deleted by mistake before submission. We have added the reference in the revised manuscript.
As for the WGD time, we do found the WGD of Juglans lineage in the walnut genome paper that published in the Plant Journal in 2016 (Martinez-Garcia et al 2016). But should be mentioned that Juglans is only a genus of Juglandaceae family but cannot represent whole family. Obviously, our WGD estimate by 4DTv (~38.9 Myr ago in Fig. 1d) is considerable younger than the previous one. However, this WGD time estimate (The Potato Genome Sequencing Consortium, 2011) is well aligned with the divergence time of Juglandaceae in the phylogenetic tree (Fig. 1a), where we corrected the time based on the divergence between the following 6-pair species from TimeTree (http://www.timetree.org/):
Aabidopsis thaliana and Carica papaya 54-90 Myr ago
Gossypium hirsutum and Theobroma cacao 32-99 Myr ago
Arabidopsis thaliana and Populus trichocarpa 107-109 Myr ago
Glycine max and Medicago truncatula 46-60 Myr ago
Morus notabilis and Prunus persica 73-90 Myr ago
Arabidopsis thaliana and Glycine max 107-111 Myr ago
We have added the missed parts in the text, please see Page 31, Line 8-10.
According to the reviewer #1's suggestion, we have changed the statement as "the WGD time of walnut was previously estimate as 60 Myr ago (Martinez-Garcia et al 2016) and that the estimates diverge a bit" in the revised manuscript (see Page 12, Line 14-15).

9. The expression analysis is reported in the methods but never given proper explanation in the results. It is sporadically mentioned throughout the section on different gene families, but the full set of DEGs isn't discussed or provided. This needs to be either given its own brief results section and accompanied by the full DEG list or left out of the manuscript.
● We thank to reviewer #1 for raising the question. Following the reviewer #1's suggestion, we have added DEG sets and relevant description in the Results section. Please see them on Page 15 in the revised main text and the DEG list were assigned as Additional file 3.

10. Page 25 "In house scripts" - this need to be posted publicly somewhere, through Gigascience or Github for example. Page 25, lines 48-57 - was this read processing done with software or with scripts? Need to specify.
● We should express our grateful to reviewer #1 for reminding this point. We have posted our scripts for PCR duplicate processing to Github (https://github.com/frankzzr/duplication_rm) and added the link in the revised manuscript (Page 25, Line 7-8). Illumina raw read processing was also done with our scripts as description in the revised manuscript, see Page 25, Line 10-12.

****
Responses to Reviewer #2
We are grateful to reviewer #2's positive comments regarding the following — "In this manuscript, the authors carry out the assembly of two important nut bearing trees, Chinese hickory and pecan. The trees have high commercial value and genome information would be important for future breeding efforts." We should express our gratitude to referee 2 for pointing out the inadequacies of methods for our manuscript. We have revised our manuscript on the full consideration of reviewer #2's comments and suggestions.
The following are our responses (following the symbol ● and marked with blue letters) to reviewer #2's comments and suggestions (following each number).

1) Are the methods appropriate to the aims of the study, are they well described, and are necessary controls included?
● We thank to reviewer #2 for raising this question and we checked the methods that we used in this study. We revised the Methods section on the full consideration of the reviewers. Now, we considered that the methods do appropriate to the aims of the study and they should be well described, and

necessary controls were included. For the details that Revierer #2 concerned, please see the below responses and the relevant description in the Methods section of the revised manuscript.

Overall, the methods used appear to be appropriate for the study. However, the Methods part lacks descriptions of many of the methods and analysis steps, namely:
1. How was the species tree in Fig. 1b estimated? How was the dating of the splits carried out?
● Sorry for missed this part in the Methods section and we should express our grateful to reviewer #2 for reminding us to fill in the missing part. The species tree in Fig. 1b was estimated as the following description:
The dating of the splits between species in Carya were carried out using MCMtree software, which is same as the estimate for divergence time between species in Fig. 1a, as described below:
To estimate the phylogenic relationship between species in Carya, the 125-bp paired-end reads in pecan and Chinese hickory, as well as the re-sequencing data of other 14 Carya species and two Juglans out-group species were re-sequenced using Illumina NextSeq 500. The raw data were processed for base calling, quality evaluation, removing the adaptor sequence, and low-quality sequence using CASAVA (v1.82) and FastQC software, with the following steps: (1) removing reads with >= 10% unidentified nucleotides (N); (2) removing reads with > 20% bases having Phred quality < 5; (3) removing reads with >10 nt aligned to the adapter, allowing <= 10% mismatches; (4) removing putative PCR duplicates generated by PCR amplification during the library construction process (i.e. read 1 and 2 of two paired-end reads that were completely identical).
The remaining high quality reads paired-end reads were mapped to the ZAFU-1 genome using BWA (Burrows-Wheeler Aligner) (Version 0.7.8) with the command 'mem -t 4 -k 32 –M'. After alignment, we performed SNP calling on a population scale using a Bayesian approach as implemented in the package SAMtools (Version 1.4). We then calculated genotype likelihoods from reads for each individual at each genomic location, and the allele frequencies in the sample with a Bayesian approach. To exclude SNP calling errors caused by incorrect mapping, only high quality SNPs (coverage depth ≥3 , RMS mapping quality ≥20, maf ≥0.05, miss≤0.1) were used for further analysis.
To clarify the phylogenetic relationship from a genome-wide perspective, an individual-based neighbor-joining (NJ) tree was constructed with 1000 bootstraps using the software TreeBestv1.9.2 (Vilella et al., 2009). The MCMCtree program implemented in the Phylogenetic Analysis by Maximum Likelihood (PAML) was applied to infer the divergence time based on the phylogenetic tree. The MCMCtree running parameters were: burn-in: model:JC69,burnin:10,000, nsample: 100,000, sampfreq:2.
Please find this part from "5.3 Distribution of hickories and phylogenetic reconstruction of Carya" in the Oneline Methods section in the revised manuscript (Page 32, Line 8 to Page 33, Line 7).

2. How were the time estimates obtained for 4DTv analysis? It is also a bit unclear whether the timing of the speciation and whole genome duplication event is obtained from this data or from some external information (in this case the reference is missing).
● We thank to reviewer #2 for paying attention to the question. In this study, 4DTv analysis and WGD time were estimated as described by The Genome Sequencing Consortium (2011). And we have filled in the missed reference in the revised manuscript. Please see them on Page 33, Line 15-16 and References section in the revised manuscript.
Reference: The Genome Sequencing Consortium (2011) Genome sequence and analysis of the tuber crop potato. Nature 475: 189-195.

3. There is no description on how the insertion time of the LTRs was estimated.
● Thanks to Reviewrer #2 for pointing out this. The insertion time of the LTRs were estimated as following:
Firstly, LTRharvest (Ellinghaus et al., 2008) and LTRfinder were used to predict LTR-RTs with the parameters: LTR length of 100-5000bp, LTRs interspace length of 1000-20000bp. Then, tRNAscan-SE was used for predicting tRNA sequences, LTRdigest (Steinbiss et al., 2009) was used for structure annotation (e.g., PBS, PPT, protein, etc.) of LTR-RTs, and the optimal annotation was achieved. In addition, LTR-RTs were clustered by usearch software with the similarity parameter of 70%. The LTR-RTs with copy number more than two or single copy containing protein domains were recruited. After that, the nucleotide variations (λ) in 5' and 3' terminals of intact LTR-RTs were estimated by MUSCLE. If λ was greater than 0.75, the intact LTR-RT would be considered invalid. For those valid intact LTR-RTs, the genetic distances (K) were calculated by $K=-0.75\ln(1-4\lambda/3)$. Finally, the insertion time of LTR-RTs was calculated based on the formula: $T=K/2r$ ($r=1.3\times10-8$ per site and per year), and distributions were further plotted.
Additional references:
Ellinghaus D, Kurtz S, Willhoeft U. LTRharvest, an efficient and flexible software for de novo detection of LTR retrotransposons. BMC Bioinformatics. 2008, 9:18.

Steinbiss S, Willhoeft U, Gremme G, Kurtz S. Fine-grained annotation and classification of de novo predicted LTR retrotransposons. Nucleic Acids Res. 2009, 37:7002-13.
The description has been integrated into the Methods section (Page 33, Line 17 to Page 34, Line 5) in the revised manuscript.

4. There is talk about GO and KEGG enrichments, but there is no description on how these were obtained. What was the statistical test for enrichment and method of multiple test correction? I would assume that the GO/KEGG assignments from InterproScan were used for this but that too needs to be stated.
Because of this the work is not reproducible and it is difficult to estimate whether the methods have been used correctly.
• Thanks to reviewer #2 for paying attention to this question. But we partially agree with the statements of reviewer #2 pointed our with regard to detailed description of GO and KEGG enrichments.
First, Gene Ontology (GO) enrichment analysis of differentially expressed genes was implemented by the GOseq R package (Young et al., 2010), in which gene length bias was corrected. GO terms with adjusted P-value less than 0.05 were considered significantly enriched by differential expressed genes, which labeled as asterisks (*). As for the reviewer #2's concern on "What was the statistical test for enrichment and method of multiple test correction?", we have descripted in our previous manuscript as "The significantly enriched GO terms were selected using a hyper-geometric test to develop hierarchical clusters of a sample tree by Euclidean Distance." (Page 35, Line 16-19).
KEGG is a database resource for understanding high-level functions and utilities of the biological system, such as the cell, the organism and the ecosystem, from molecular-level information, especially large-scale molecular datasets generated by genome sequencing and other high-through put experimental technologies (http://www.genome.jp/kegg/). We used KOBAS software to test the statistical enrichment of differential expression genes in KEGG Pathways (Mao et al., 2005). Moreover, we confirmed that our analysis were all repeatable using the same software for a specialist on bioinformatics analysis, so our results should be reliable. We have filled this description in the Materials and Methods section of the revised manuscript (Page 35, Line 22 to Page 36, Line 1).
References:
Young, M. D., Wakefield, M. J., Smyth, G. K., and Oshlack, A. (2010). Gene ontology analysis for RNA-seq: accounting for selection bias. Genome Biology. 11:R14.
Mao, X., Cai, T., Olyarchuk, J.G., Wei, L. (2005). Automated genome annotation and pathway identification using the KEGG Orthology (KO) as a controlled vocabulary. Bioinformatics. 21 (19): 3787-3793.

2) Are the conclusions adequately supported by the data shown?
Because of poor description of the methods it is difficult to estimate the soundness of the results. However, even if these are correct the results are also reported poorly:
1. GO enrichments are reported only in Figures S5 and S7. Are all of these significant enrichments? What are then the asterisks (*) in the plots? In any case, the statement "p15, line 6-9: We found that the significantly enriched genes were involved in GO terms of ion transport (pecan) and defense response (Chinese hickory)" certainly doesn't correspond with the categories reported in Figure S7. They are there but there is quite a lot more. Why raise only these two categories?
• Should be confirmed that only the GO terms with asterisks are significant enriched In Figures S5 and S7 in our previous manuscript. We are very grateful to reference #2 for his pointing out the errors related to the statement of the Figures. Based on the suggestions of both reviewers, we re-did the gene family expansion/contraction analysis that included silver birch and grape genome information (Fig. S6). As the reviewer #2 mentioned that GO terms didn't correspond with the categories reported in Figure S7. We found that we mistakenly described the results from GO enrichments and KEGG pathway enrichment when we prepared the previous manuscript, which led to errors finally. As for the reason that we highlighted these two categories is to explain their abiotic (for pecan) or biotic (for Chinese hickory) resistant traits at the beginning of preparing the manuscript, as we introduced in the background for the species. And we have all corrected these in the revised manuscript. (Please see Page 35, Line 16-19).

2. Also: p15, line 17-20: "GO enrichment of the significantly expanded and species-specific gene families in both species highlighted gene function in stress response". I don't see this in the GO figures. There's a small gene set associated with ROS metabolism in pecan (S5) and defence response in Chinese hickory (S7) but they certainly don't rise out as the major pattern. Especially defence response is a general category, it might be good to check which genes were expanded.
• We are very grateful to Reviewrer #2 for pointing out the error. And as the reviewer #2 pointed out that genes involved in ROS metabolism and defence response did represent a small set but not the

major pattern. Therefore, to avoid misunderstanding, we deleted the statement and changed our description about this part in the revised manuscript. Please see Page 14, Line 6-8.

R genes	Arabidopsis thaliana	Annotated copy number		Filtered copy number	
		'Pawnee'	ZAFU-1	'Pawnee'	ZAFU-1
CC-NBS	4	15	22	13	18
CC-NBS-LRR	35	52	55	51	54
CC-TIR-NBS-LRR	0	1	0	1	0
LRR-NBS-LRR	0	2	2	2	2
LRR-TIR-NBS-LRR	0	2	0	2	0
NBS	3	92	102	83	97
NBS-LRR	22	159	171	147	155
NBS-LRR-NBS	0	2	0	2	0
NBS-LRR-TIR	1	0	1	0	1
NBS-LRR-NBS-LRR	0	3	0	3	0
NBS-LRR-TIR-NBS	0	1	0	1	0
NBS-LRR-TIR-NBS-LRR	0	2	0	2	0
NBS-TIR-NBS-LRR	0	1	0	1	0
TIR-CC-NBS-LRR	2	1	1	1	1
TIR-NBS	16	28	30	27	27
TIR-NBS-LRR	80	91	99	91	90
TIR-NBS-LRR-NBS-LRR	0	1	2	1	2
TIR-NBS-LRR-NBS-TIR-NBS-LRR	0	0	1	0	1
TIR-NBS-LRR-TIR	1	1	2	1	1
TIR-NBS-LRR-TIR-NBS-LRR	1	0	2	0	2
TIR-NBS-TIR-NBS-LRR	2	0	0	0	0
TIR-NBS-TIR-NBS	0	0	1	0	1
total	167	454	491	429	452

Regarding the gene expansions, it would be good to check whether they are real or artifacts of the over-assembled genome (heterozygosity). For example it would be good to check whether the coverage of the R genes (known to be highly heterozygous) is OK, or if there are drops to 50% coverage indicating separate assembly of heterozygous alleles.

• We agree with the reviewer #2's suggestion to check whether they are real or artifacts of the over-assembled genome. Because of the high heterozygosity of the genomes, it's possible that the protein-coding gene set existed heterozygous alleles.

In comparing with 'Pawnee', we found more R gene copies in ZAFU-1 genome, although 'Pawnee' with higher heterozygosity then ZAFU-1. This result indicates that the larger R-gene family in ZAFU-1 should be the feature of species but not because of hetrozygosity in the genome. Following the reviewer #2's suggestion, we randomly selected 90X Illumina Hiseq X-ten data and aligned the reads to all the putative R genes and the coverage of each gene were calculated. After that we filtered out the genes with coverage less than 40X (see the below table). We found that the copy number dropped in both species but the copies in ZAFU-1 is still larger that in 'Pawnee'. These analyses suggest that our conclusion is reliable.

We were also trying to get the real number for gene family that we are interested in. However, even if so, the real gene number of R gene family should not drop to existing number. Moreover, to confirm the real number needs a long period that would be a separate project.

About conclusions on gene family expansions in the different genera, all Fagales representatives are from family Juglandaceae. At least one representative from some other family should be included, for example silver birch. Otherwise the conclusions will be specific to the family, not the order. This would make a good contrast, since birch has not undergone whole genome duplications. Then vitis should be also used to provide rooting for the tree.

• We are very grateful to reviewer #2's for the suggestion on gene family expansions in the different genera and using vitis as root for the phylogeny tree. In the revised manuscript, we have reconstructed the phylogenic relationship and re-estimated the gene family expansion including silver birch and grape. We also changed the description and discussion accordingly; please see the last paragraph on Page 11 (Line 17) and the last paragraph on Page 13 (Line 15-16) in the main text and Fig. S6 in Additional file 2.

Close
