# Supplementary material for: The genomes of pecan and Chinese hickory provide insights into Carya evolution and nut nutrition
Source: Gigascience. 2019 May 2;8(5):giz036. doi: 10.1093/gigascience/giz036 (PMC6497033; doi:10.1093/gigascience/giz036)
Supplement: Response_to_Reviewer_Comments_Revision_1.pdf [file giz036_response_to_reviewer_comments_revision_1.pdf]

Author's Response To Reviewer Comments

Close

Dear Professor, Dr. Laurie Goodman, Editor-in-Chief of GigaScience:
Thank you for accepting our manuscript (GIGA-D-18-00185) for publication in GigaScience, contingent on approval of minor revisions. We also thank the reviewers for their valuable comments and suggestions for improving our manuscript. We have revised the document fully based on reviewers' comments and suggestions (shown as red text in the main document and in additional supplementary files). In the cover letter, we provide responses to the reviewers' comments and suggestions point by point.
In response to reviewer #1's comments, we also checked the manuscripts and additional files carefully and made additional revisions on some typos. The language has been polished by collaborators, who are native English speakers.
We hope the revisions incorporated here allow the manuscript to be accepted for publication in GigaScience.

With Best Regards

Yours Sincerely

Corresponding authors
Drs. Jianqin Huang, Lihong Xiao and L.J. Grauke

The following pages are the responses to the reviewers' comments and suggestions.

Responses to the reviewers' comments and suggestions

Responses to Reviewer #1
We are grateful to reviewer #1 for valuable comments and suggestions and have given full consideration to all of them.
Our responses to the reviewer's numbered comments are in blue text following the symbol ●.

#3. The authors did not correctly interpret my original comment for the rRNA gene counts. I am not questioning their method of identifying the genes, I am questioning if the assembly is correct and able to represent the true number of rRNA genes. Large tandem arrays, similar to other types of repetitive elements, are difficult to assemble. Likely many copies of the genes are collapsed into a single consensus sequence, which you can detect by looking at sequence coverage in the region. This makes it difficult or impossible to compare rRNA gene copy number between the two species. However, the text in the results highlighting this difference is gone, so I think it is now fine. I do not request a revision, just want to clarify my original comments.

• We thank reviewer #1 for raising the issue of accuracy in rRNA gene counts and are pleased that our last response was satisfactory.

#4. The response to reviewers indicates "mitochondria and chloroplast genomes… we have filtered out the reads and obtained the chloroplast genome sequences and annotations before scaffolding". This filtering needs to be included in the methods. The authors also state that "we didn't include the chloroplast genomes in this manuscript" but the manuscript additional file list includes "Additional file 4: An excel file with chloroplast sequences of pecan and Chinese hickory". However, I can't download that file, so I'm not sure it was submitted. This is an important a decision - if the chloroplast is a part of this paper, it needs to be included in the methods and the results and it needs to be submitted to a public repo, preferably with the assembly in NCBI. If you do not want to include it in this manuscript, then it needs to be removed from the supplementary materials, but the methods should still mention these reads were filtered out.
• As mentioned in our last responses: 'We have filtered out the reads and obtained the chloroplast genome sequences and annotations before scaffolding. But unfortunately, these assemblies and annotations showed significant difference with our assemblies from other two varieties of pecan (unpublished data). To confirm if or not the differences are true will need extensive experiments and more time. Therefore, we didn't include the chloroplast genomes in this manuscript.' Therefore, we don't intend to include the organelle genomes in the current manuscript and they have been removed from supplementary materials.
Chloroplast reads were filtered out before we assembled the genome sequences of the two Carya species using two steps as follows: (1) Illumina reads were mapped to Juglans regia chloroplast reference genome (GenBank: MF167464.1) using BWA (v0.5.9-r16) with default parameters; (2) PacBio reads were mapped to J. regia chloroplast reference genome using BLASR with default parameters. The methods were added in Section '1.2 DNA extraction and whole genome sequencing' (Please see Page 25, Line 6-10).

#9. Expression analysis. The reviewers did add this to the results section, which is great. However, I still find it unclear. The text refers to up and down regulated genes, but I'm not clear on if they were up regulated in various individual time points in comparison to other time points or in one species versus the other or both. Scripts that were used to run DESeq2 or at least the statistical model used would be nice to include in the methods. For example, if the model was ~time within each species, that would report any transcript responding to time. With three time points, that is a bit more complicated than up and down (ie, could very high in time point 1, low in time point 2, moderate in time point 3). Or if contrasts were performed between individual time points (upregulated in time point 2 in comparison to time point 1). This doesn't need to be a focus point of the paper, but I do think its imporant to be clear about what was done and how it was done.
• We thank reviewer #1 for raising this question. We conducted transcriptomic analysis during embryo development and identified differentially expressed genes (DEGs) in pecan and Chinese hickory, respectively. Levels of DEGs significantly vary among three developmental stages (i.e., the early and fully extended stages of cotyledon development, and the fully matured stage of the embryos, designated as PEY1-3 in pecan and HEY1-3 in Chinese hickory). In detail, the DEGs were identified from PEY2 vs PEY1, and PEY3 vs PEY2 in pecan and from HEY2 vs HEY1, and HEY3 vs HEY2 in Chinese hickory. The number and the result of GO enrichment of these DEGs were added in this revised manuscript (see Page 15, Line 3 to Line 22) and in additional file 2 (see Fig. S9-S12).

#8 page 12 lines 14-15. "The WGD time of walnut was previously estimate at 60Mya and that the estimates diverge a bit" needs to be reworded. Yes, I put that latter phrase in my original review, but it was a comment that I didn't expect the authors to use in the paper. I do not believe it makes sense as used in the paper or has enough precision to appear in the results of a scientific paper. This sentence also has some grammatical errors.
• We apologized for misunderstood your question. We carefully read your original review and the article of walnut genome and related references therein (Martinez-Garcia et al., 2016). We found that the WGD of Juglans lineage (~66 Myr ago) in the walnut genome paper that published in the Plant Journal (Martinez-Garcia et al 2016) were estimated based on fossils record. Moreover, Zhang et al. (2013) used fossils of Carya that are clearly distinguished from those of Juglans based on morphology, that were recovered from particular geological strata that are dated using internationally recognized chronology, to estimate dates of origin for the genus. Their time estimate is based on fossils of the two genera that are distinct and found in strata that are dated with internationally recognized chronology. The 4DTv distance in our manuscript (~38.9 Myr ago) is calibrated using methods reported for potato. These results lead us to consider that estimate of 66 Ma for separation of Carya from Juglans is more reliable. Therefore, we re-calibrated the WGD events in Carya species (Fig. 1d) by using 66 Myr as a comparator, and corrected

the date in the phylogenetic trees of species in Fig. 1a and Fig. 1b. Meanwhile, we also revised the description in the manuscript accordingly, see Page 11, Line 17-18; Page 12, Line 16 and Line 18.
Additional revisions:
● To present our results more accurately, we revised several typos in the last version of our manuscript, which were remarked in the main text and several were listed as below:
1. In additional file 1: Table S1, “insertsieze” corrected as “Insert size”
2. In additional file 1: Table S2, “sampling ate” corrected as “Sampling date”
3. In additional file 1: Table S3, we added two rows “Total clean data (Gb)” and “Sequence coverage of clean data (X) ”. Correspondingly, we revised the number in abstract of page 4, lines 16-17 and line 22 in page 8, lines 1 in page 9, line 17 in page 25, line 6 in page 26 and line 3-4 in page 28.
4. We deleted a redundant Bioproect ID PRJNA427736 in “Availability of data and materials” in Page 36.
5. We corrected a typo “clariefy” to “clarify” in page 12.
6. We added a grant in “Funding” section in page 37, which we missed in the original manuscript.

Close
