# Supplementary material for: The genomes of pecan and Chinese hickory provide insights into Carya evolution and nut nutrition
Source: Gigascience. 2019 May 2;8(5):giz036. doi: 10.1093/gigascience/giz036 (PMC6497033; doi:10.1093/gigascience/giz036)
Supplement: Reviewer_1_Report_Original_Submission -- Meg Staton — 6/19/2018 Reviewed [file giz036_reviewer_1_report_original_submission.pdf]

Reviewer Report

Title: The genomes of pecan and Chinese hickory provide insights into Carya evolution and nut nutrition

Version: Original Submission **Date:** 6/19/2018

Reviewer name: Meg Staton

Reviewer Comments to Author:

While not a huge problem to readability, a few English grammar issues remain. The manuscript would benefit from a final editing round by a native english speaker.
Brief descriptions for supplementary tables and figures would help with clarity. Additional File 1, S6 and S7 titles are missing word "of". Figure S2 is missing a label for the green line (presumably C. il).
5S rRNAs are reported as having a larger number in ZAFU than Pawnee - how was this determined? As 5S rRNAs are usually present in large tandem arrays with little to no variation, I wouldn't expect an assembly to yield a very accurate count of rRNAs by itself
Did the authors look for or extract the mitochondria or chloroplast genomes? These would likely have been captured in scaffolds during assembly
Although this is background material and not the point of the paper, I'm confused by the apomixis part of the introduction. The statement "nucellar embryony that demonstrates remarkable resistance to fungal diseases" - do you mean that the trait of disease resistance can be passed down easily through nucellar embryony or that nucellar embryony actual functions in preventing disease directy? Apomixis is mentioned again as very good for breeding, but I would argue it might be good for passing down traits in production but terrible for breeding (no recombination, right?). Also, apoximis is misspelled on Page 8 Line 45.
Data Description - As this is a required section by GigaScience, I don't think it's appropriate to ignore it (currently it says only to refer to the methods). Here's what it's supposed to contain: "A statement providing background and purpose for collection of these data should be presented for readers without specialist knowledge in that area. A brief description of the protocol for data collection, data curation and quality control, as well as potential uses should be included, as well as outlining how the data can be accessed if it is not deposited in our repository."
Boea hygrometrica pops up as a comparator in a few places, particularly in the transposable elements. But that is an asterid, so it's really not a particularly compelling comparator for a rosid. The comparison to other species either needs to span a wide range of plants from all clades or focus on reference genomes from more closely related rosids (walnut, peach, poplar, etc)
The walnut genome is not mentioned or cited (Martinez-Garcia et al 2016), despite being used extensively as a comparator. That paper put the Juglandaceae WGD at 60MYA, considerably older than this estimate. These things are not exact, but a brief mention that the WGD was previously found in walnut and that the estimates diverge a bit would be good.
The expression analysis is reported in the methods but never given proper explanation in the results. It is sporadically mentioned throughout the section on different gene families, but the full set of DEGs isn't

discussed or provided. This needs to be either given its own brief results section and accompanied by the full DEG list or left out of the manuscript.
Page 25 "In house scripts" - These need to be posted publicly somewhere, through Gigascience or Github for example
Page 25, lines 48-57 - was this read processing done with software or with scripts? Need to specify

Methods

Are the methods appropriate to the aims of the study, are they well described, and are necessary controls included? Choose an item.

Conclusions

Are the conclusions adequately supported by the data shown? Choose an item.

Reporting Standards

Does the manuscript adhere to the journal's guidelines on [minimum standards of reporting?]() Choose an item.

Choose an item.

Statistics

Are you able to assess all statistics in the manuscript, including the appropriateness of statistical tests used? Choose an item.

Quality of Written English

Please indicate the quality of language in the manuscript: Choose an item.

Declaration of Competing Interests

Please complete a declaration of competing interests, considering the following questions:

- Have you in the past five years received reimbursements, fees, funding, or salary from an organisation that may in any way gain or lose financially from the publication of this manuscript, either now or in the future?
- Do you hold any stocks or shares in an organisation that may in any way gain or lose financially from the publication of this manuscript, either now or in the future?
- Do you hold or are you currently applying for any patents relating to the content of the manuscript?
- Have you received reimbursements, fees, funding, or salary from an organization that holds or has applied for patents relating to the content of the manuscript?
- Do you have any other financial competing interests?

- Do you have any non-financial competing interests in relation to this paper?

If you can answer no to all of the above, write 'I declare that I have no competing interests' below. If your reply is yes to any, please give details below.

I declare that I have no competing interests

I agree to the open peer review policy of the journal. I understand that my name will be included on my report to the authors and, if the manuscript is accepted for publication, my named report including any attachments I upload will be posted on the website along with the authors' responses. I agree for my report to be made available under an Open Access Creative Commons CC-BY license (http://creativecommons.org/licenses/by/4.0/). I understand that any comments which I do not wish to be included in my named report can be included as confidential comments to the editors, which will not be published.

I agree to the open peer review policy of the journal

To further support our reviewers, we have joined with Publons, where you can gain additional credit to further highlight your hard work (see: https://publons.com/journal/530/gigascience). On publication of this paper, your review will be automatically added to Publons, you can then choose whether or not to claim your Publons credit. I understand this statement.

Yes Choose an item.
