# Supplementary material for: The genomes of pecan and Chinese hickory provide insights into Carya evolution and nut nutrition
Source: Gigascience. 2019 May 2;8(5):giz036. doi: 10.1093/gigascience/giz036 (PMC6497033; doi:10.1093/gigascience/giz036)
Supplement: Reviewer_1_Report_Revision_1 -- Meg Staton — 11/21/2018 Reviewed [file giz036_reviewer_1_report_revision_1.pdf]

Reviewer Report

Title: The genomes of pecan and Chinese hickory provide insights into Carya evolution and nut nutrition

Version: Revision 1 **Date:** 11/21/2018

Reviewer name: Meg Staton

Reviewer Comments to Author:

The authors have responded to the majority of my concerns, but I still have a few small revisions to request.
#3. The authors did not correctly interpret my original comment for the rRNA gene counts. I am not questioning their method of identifying the genes, I am questioning if the assembly is correct and able to represent the true number of rRNA genes. Large tandem arrays, similar to other types of repetitive elements, are difficult to assemble. Likely many copies of the genes are collapsed into a single consensus sequence, which you can detect by looking at sequence coverage in the region. This makes it difficult or impossible to compare rRNA gene copy number between the two species. However, the text in the results highlighting this difference is gone, so I think it is now fine. I do not request a revision, just want to clarify my original comments.
#4. The response to reviewers indicates "mitochondria and chloroplast genomes… we have filtered out the reads and obtained the chloroplast genome sequences and annotations before scaffolding". This filtering needs to be included in the methods.
The authors also state that "we didn't include the chloroplast genomes in this manuscript" but the manuscript additional file list includes "Additional file 4: An excel file with chloroplast sequences of pecan and Chinese hickory". However, i can't download that file, so I'm not sure it was submitted. This is an important a decision - if the chloroplast is a part of this paper, it needs to be included in the methods and the results and it needs to be submitted to a public repo, preferably with the assembly in NCBI. If you do not want to include it in this manuscript, then it needs to be removed from the supplementary materials, but the methods should still mention these reads were filtered out.
#9. Expression analysis. The reviewers did add this to the results section, which is great. However, I still find it unclear. The text refers to up and down regulated genes, but I'm not clear on if they were up regulated in various individual time points in comparison to other time points or in one species versus the other or both. Scripts that were used to run DESeq2 or at least the statistical model used would be nice to include in the methods. For example, if the model was ~time within each species, that would report any transcript responding to time. With three time points, that is a bit more complicated than up and down (ie, could very high in time point 1, low in time point 2, moderate in time point 3). Or if contrasts were performed between individual time points (up regulated in time point 2 in comparison to time point 1). This doesn't need to be a focus point of the paper, but I do think its imporant to be clear about what was done and how it was done.
page 12 lines 14-15. "The WGD time of walnut was previously estimate at 60Mya and and that the estimates diverge a bit" needs to be reworded. Yes, I put that latter phrase in my original review, but it

was a comment that I didn't expect the authors to use in the paper. I do not believe it makes sense as used in the paper or has enough precision to appear in the results of a scientific paper. This sentence also has some grammatical errors.

Methods

Are the methods appropriate to the aims of the study, are they well described, and are necessary controls included? Choose an item.

Conclusions

Are the conclusions adequately supported by the data shown? Choose an item.

Reporting Standards

Does the manuscript adhere to the journal's guidelines on minimum standards of reporting? Choose an item.

Choose an item.

Statistics

Are you able to assess all statistics in the manuscript, including the appropriateness of statistical tests used? Choose an item.

Quality of Written English

Please indicate the quality of language in the manuscript: Choose an item.

Declaration of Competing Interests

Please complete a declaration of competing interests, considering the following questions:

- Have you in the past five years received reimbursements, fees, funding, or salary from an organisation that may in any way gain or lose financially from the publication of this manuscript, either now or in the future?
- Do you hold any stocks or shares in an organisation that may in any way gain or lose financially from the publication of this manuscript, either now or in the future?
- Do you hold or are you currently applying for any patents relating to the content of the manuscript?
- Have you received reimbursements, fees, funding, or salary from an organization that holds or has applied for patents relating to the content of the manuscript?
- Do you have any other financial competing interests?
- Do you have any non-financial competing interests in relation to this paper?

If you can answer no to all of the above, write 'I declare that I have no competing interests' below. If your reply is yes to any, please give details below.

I declare that I have no competing interests

I agree to the open peer review policy of the journal. I understand that my name will be included on my report to the authors and, if the manuscript is accepted for publication, my named report including any attachments I upload will be posted on the website along with the authors' responses. I agree for my report to be made available under an Open Access Creative Commons CC-BY license (http://creativecommons.org/licenses/by/4.0/). I understand that any comments which I do not wish to be included in my named report can be included as confidential comments to the editors, which will not be published.

I agree to the open peer review policy of the journal

To further support our reviewers, we have joined with Publons, where you can gain additional credit to further highlight your hard work (see: https://publons.com/journal/530/gigascience). On publication of this paper, your review will be automatically added to Publons, you can then choose whether or not to claim your Publons credit. I understand this statement.

Yes Choose an item.
