# Supplementary material for: The genomes of pecan and Chinese hickory provide insights into Carya evolution and nut nutrition
Source: Gigascience. 2019 May 2;8(5):giz036. doi: 10.1093/gigascience/giz036 (PMC6497033; doi:10.1093/gigascience/giz036)
Supplement: Reviewer_2_Report_Original_Submission -- Jarkko Salojarvi, DSc (tech) — 6/23/2018 Reviewed [file giz036_reviewer_2_report_original_submission.pdf]

**Reviewer Report**

**Title: The genomes of pecan and Chinese hickory provide insights into Carya evolution and nut nutrition**

**Version: Original Submission** **Date:** 6/23/2018

**Reviewer name: Jarkko Salojarvi, DSc (tech)**

**Reviewer Comments to Author:**

In this manuscript, the authors carry out the assembly of two important nut bearing trees, Chinese hickory and pecan. The trees have high commercial value and genome information would be important for future breeding efforts. However, the manuscript is lacking detail and it is difficult to follow whether the results lead in to the conclusions suggested in the paper. More specifically:

1) Are the methods appropriate to the aims of the study, are they well described, and are necessary controls included?

Overall, the methods used appear to be appropriate for the study. However, the Methods part lacks descriptions of many of the methods and analysis steps, namely:

1. How was the species tree in Fig 1b estimated? How was the dating of the splits carried out?
2. How were the time estimates obtained for 4DTv analysis? It is also a bit unclear whether the timing of the speciation and whole genome duplication event is obtained from this data or from some external information (in this case the reference is missing).
3. There is no description on how the insertion time of the LTRs was estimated.
4. There is talk about GO and KEGG enrichments, but there is no description on how these were obtained. What was the statistical test for enrichment and method of multiple test correction? I would assume that the GO/KEGG assignments from interproscan were used for this but that too needs to be stated.

Because of this the work is not reproducible and it is difficult to estimate whether the methods have been used correctly.

2) Are the conclusions adequately supported by the data shown?

Because of poor description of the methods it is difficult to estimate the soundness of the results. However, even if these are correct the results are also reported poorly:

1. GO enrichments are reported only in Figures S5 and S7. Are all of these significant enrichments? What are then the asterisks (*) in the plots? In any case, the statement "p15, line 6-9: We found that the significantly enriched genes were involved in GO terms of ion transport (pecan) and defense response (Chinese hickory)" certainly doesn't correspond with the categories reporten in Figure S7. They are there but there is quite a lot more. Why raise only these two categories?
2. Also: p15, line 17-20: "GO enrichment of the significantly expanded and species-specific gene families in both species highlighted gene function in stress response". I don't see this in the GO figures. There's a small gene set associated with ROS metabolism in pecan (S5) and defence response in Chinese hickory (S7) but they certainly don't rise out as the major pattern. Especially defence response is a general category, it might be good to check which genes were expanded.

Regarding the gene expansions, it would be good to check whether they are real or artefacts of the overassembled genome (heterozygosity). For example it would be good to check whether the coverage of the R genes (known to be highly heterozygous) is OK, or if there are drops to 50% coverage indicating separate assembly of heterozygous alleles.
About conclusions on gene family expansions in the different genera, all Fagales representatives are from family Juglandaceae. At least one representative from some other family should be included, for example silver birch. Otherwise the conclusions will be specific to the family, not the order. This would make a good contrast, since birch has not undergone whole genome duplications. Then vitis should be also used to provide rooting for the tree.
Re comparison of TE abundances, why compare against B. hygrometrica (Lamiales) and Poales? They are phylogenetically much more distant than for example birch or even vitis.
3) Please indicate the quality of language in the manuscript. Does it require a heavy editing for language and clarity?
Language is OK. There are some mistakes which could be corrected, though.
4) Are you able to assess all statistics in the manuscript, including the appropriateness of statistical tests used?
No, due to the lacks described above.

**Methods**

Are the methods appropriate to the aims of the study, are they well described, and are necessary controls included? Choose an item.

**Conclusions**

Are the conclusions adequately supported by the data shown? Choose an item.

**Reporting Standards**

Does the manuscript adhere to the journal's guidelines on minimum standards of reporting? Choose an item.

Choose an item.

**Statistics**

Are you able to assess all statistics in the manuscript, including the appropriateness of statistical tests used? Choose an item.

**Quality of Written English**

Please indicate the quality of language in the manuscript: Choose an item.

**Declaration of Competing Interests**

Please complete a declaration of competing interests, considering the following questions:

- Have you in the past five years received reimbursements, fees, funding, or salary from an organisation that may in any way gain or lose financially from the publication of this manuscript, either now or in the future?
- Do you hold any stocks or shares in an organisation that may in any way gain or lose financially from the publication of this manuscript, either now or in the future?
- Do you hold or are you currently applying for any patents relating to the content of the manuscript?
- Have you received reimbursements, fees, funding, or salary from an organization that holds or has applied for patents relating to the content of the manuscript?
- Do you have any other financial competing interests?
- Do you have any non-financial competing interests in relation to this paper?

If you can answer no to all of the above, write 'I declare that I have no competing interests' below. If your reply is yes to any, please give details below.

Click here to enter text.

I agree to the open peer review policy of the journal. I understand that my name will be included on my report to the authors and, if the manuscript is accepted for publication, my named report including any attachments I upload will be posted on the website along with the authors' responses. I agree for my report to be made available under an Open Access Creative Commons CC-BY license (http://creativecommons.org/licenses/by/4.0/). I understand that any comments which I do not wish to be included in my named report can be included as confidential comments to the editors, which will not be published.

Choose an item.

To further support our reviewers, we have joined with Publons, where you can gain additional credit to further highlight your hard work (see: https://publons.com/journal/530/gigascience). On publication of this paper, your review will be automatically added to Publons, you can then choose whether or not to claim your Publons credit. I understand this statement.

Yes Choose an item.
